# SHIELD: A weakly supervised graph attention neural network for decoding disease-relevant cell-cell interactions

**DOI:** 10.1016/j.patter.2026.101562

**Published:** 2026-05-22

**Authors:** Vivek Sehra, Benjamin Ruf, Gabriel Duval, Sepideh Babaei, Manfred Claassen

**Affiliations:** 1 Department of Internal Medicine I, University Hospital Tübingen, Eberhard Karls University of Tübingen, 72076 Tübingen, Germany; 2 M3 Research Center, University Hospital Tübingen, Eberhard Karls University of Tübingen, 72076 Tübingen, Germany; 3 Institute for Bioinformatics and Medical Informatics, Eberhard Karls University of Tübingen, 72076 Tübingen, Germany; 4 Department of Computer Science, Eberhard Karls University of Tübingen, 72076 Tübingen, Germany; 5 Cluster of Excellence iFIT (EXC 2180) “Image-Guided and Functionally Instructed Tumor Therapies,” University of Tübingen, 72076 Tübingen, Germany

**Keywords:** graph attention network, spatial biology, single-cell biology, highly multiplexed tissue imaging, cancer, diabetes

## Abstract

Multiplexed tissue imaging enables detailed study of cell-cell interactions in disease, yet systematic, interpretable, and supervised computational methods for inferring such interactions remain scarce. We present SHIELD (spatially enhanced immune landscape decoding), a graph attention network framework that quantifies disease-relevant cell-cell interactions through learned attention scores, without relying on prior biological assumptions such as ligand-receptor databases. Validated across three multiplexed tissue imaging datasets—hepatocellular carcinoma (HCC), colorectal cancer (CRC), and type 1 diabetes (T1D)—SHIELD identifies rare mucosal-associated invariant T (MAIT) cell-macrophage interactions in HCC, suppressive CD8^+^ T cell-macrophage interactions enriched in CRC non-responders, and β cell interactions with cytotoxic and helper T cells across T1D disease stages. In all contexts, SHIELD reconstructs known and biologically meaningful interactions, offering a robust, interpretable tool for spatial tissue analysis and data-driven mechanistic discovery.

## Highlights

• SHIELD is a graph attention network for interpretable inference of cell interactions
• SHIELD learns cell interactions without biological priors
• SHIELD discovers rare, disease-associated immune cell communication
• Validated across multiple disease entities and different imaging platforms

## The bigger picture

Multiplexed tissue imaging has enabled the mapping out of the cellular composition and spatial architecture of tissues and organs. However, translating these massive spatial datasets into actionable biological insights—specifically, identifying which cell-to-cell interactions are associated and possibly drive a disease—remains a challenge. Current deep learning models often act as opaque “black boxes,” while traditional spatial analyses usually require rigid prior biological assumptions or merely detect frequent cellular co-occurrence, missing interactions that are rare but functionally critical.

To overcome this bottleneck, we introduce SHIELD (spatially enhanced immune landscape decoding), an interpretable computational framework built on graph attention networks, a type of AI that focuses on how nearby cells relate to one another. Rather than just mapping where cells are, SHIELD helps us discover how their interactions drive disease. By utilizing a weakly supervised learning approach, the model learns to assign “attention scores” to cellular neighborhoods, systematically quantifying the relevance of pairwise cell interactions to a specific disease state. Crucially, SHIELD operates entirely without the need for predefined anchor cells or existing lists of cell-cell signals, allowing for unbiased, systematic discovery. The broader impact of this framework is its ability to extract subtle, rare, and highly directional immune communication from complex imaging data. SHIELD bridges the gap between high-dimensional spatial omics and actionable biological hypotheses, ultimately enabling researchers to decode the spatial rules governing immunity, therapy resistance, and disease progression across diverse clinical landscapes.

## Teaser

How do cells communicate in health and disease? Sehra et al. introduce SHIELD, a machine learning model decoding intercellular communication directly from multiplexed tissue images. Unlike traditional “black-box” models, SHIELD is inherently interpretable and operates without biological priors. Validated across multiple cancer entities and type 1 diabetes using different imaging technologies, this tool enables researchers to map the hidden cell communication rules of tissue function in health and disease, ultimately turning complex tissue imaging data into potentially actionable medical insights.

## Introduction

Multiplexed tissue imaging technologies^1^^,^^2^^,^^3^ have revolutionized our ability to profile cellular composition and spatial organization at single-cell resolution.^4^ These methods enable precise cell mapping, cell-type annotation, and visualization of tissue microenvironments in both healthy and diseased states. Multiplexed tissue imaging data are becoming increasingly important for understanding how tissue architecture and cell-cell interactions confer disease mechanisms. These insights are not obvious from the primary imaging data and require additional computational analysis to this end. There remains a lack of computational approaches that enable systematic and interpretable inference of cell-cell interactions, potentially including disease associations.

Deep learning approaches have been proposed for the interpretation of multiplexed tissue imaging data, leveraging convolutional neural networks (CNNs), transformers, and adversarial learning frameworks to extract complex spatial patterns.^5^^,^^6^^,^^7^^,^^8^^,^^9^ These methods have enabled more automated feature extraction from spatially resolved omics data. DeepST,^5^ for example, integrates morphological images, gene expression profiles, and spatial locations using adversarial learning, improving multimodal integration. Similarly, transformer-based models^7^^,^^8^ have enhanced spatial transcriptomics classification, capturing long-range spatial dependencies. A persisting limitation across these deep learning methods is interpretability, particularly with respect to cell-cell interaction inference. While these models capture complex spatial dependencies, they function as “black-box” architectures, making it difficult to determine which specific cellular interactions contribute to disease phenotypes.

Graph-based approaches have been employed to model spatial cell-cell interactions, as they allow cells to be represented as nodes and spatial proximities as edges. Early methods, such as direct neighborhood analysis,^10^^,^^11^^,^^12^ assessed interactions based on predefined spatial neighborhoods to identify significant cell-cell relationships. More advanced techniques, including graph neural networks (GNNs), have been developed to integrate spatial and molecular information, enabling tissue classification, niche identification, and disease characterization.^13^^,^^14^^,^^15^^,^^16^^,^^17^ scNiche^18^ advanced niche identification by integrating multi-view graph representations and leveraging graph autoencoders for spatial feature fusion, improving tissue microenvironment characterization. BANYAN^19^ employed Bayesian network modeling to analyze community connectivity, quantifying spatially distinct cellular interactions in transcriptomic datasets. While these methods have enhanced niche detection and community-based analysis, they primarily focus on spatial domain segmentation rather than interpretable cell type-cell type interaction inference at a fine-grained level and lack association with external phenotypes such as disease states.

More recently, multi-omics integration approaches have leveraged graph-based models to improve tissue architecture resolution. SpatialGlue^20^ introduced a dual-attention mechanism to integrate transcriptomic, proteomic, and epigenomic data, outperforming traditional multimodal alignment methods by preserving spatial structures. scHolography^21^ reconstructed single-cell spatial neighborhoods in 3D using deep learning, enabling high-resolution visualization of tissue organization. While these approaches offer significant improvements in multi-omics integration and tissue-level mapping, they do not directly quantify cell-cell interactions.

Moreover, existing frameworks that do quantify interactions—such as those based on ligand-receptor expression^22^^,^^23^—require prior knowledge and integration of curated signaling databases, which must be embedded in the experimental design and are therefore not agnostic to the underlying data. This limits their flexibility, especially on imaging-based platforms where such transcript-level information is typically not available. In contrast, models capable of inferring interactions directly from spatial context without relying on predefined molecular priors remain scarce.

The above approaches typically rely on unsupervised analysis of multiplexed tissue imaging data, which limits their ability to directly learn disease associations. Consequently, disease-associated tissue architectures are often inferred post hoc through differential analysis between conditions. Recently, S^3^-CIMA was introduced, a supervised approach for directly learning cell-type-specific cell-cell interactions in a supervised learning setup.^24^ However, this approach requires the definition of predefined anchor cells, restricting its ability to systematically infer interactions. As a result, it investigates only one anchor cell type at a time, requiring model reconfiguration for each cell-cell interaction of interest.

In summary, computational methods for systematic, supervised, and interpretable inference of cell type-cell type communication are lacking. To address this, we developed SHIELD (spatially enhanced immune landscape decoding), a model that systematically infers cell type-cell type interactions using graph attention networks (GATs). Unlike previous models, SHIELD assigns weighted edge values through attention mechanisms, quantifying the relevance of each cell type-cell type interaction in a data-driven manner. This approach ensures that all interactions are considered simultaneously without requiring predefined anchor cells or other prior biological assumptions, enabling the discovery of immune interactions critical for disease progression. We validate SHIELD across three distinct disease contexts, i.e., hepatocellular carcinoma (HCC), type 1 diabetes (T1D), and colorectal cancer (CRC), demonstrating its ability to reconstruct known immune cell type-cell type interactions.

## Results

First, we provide an overview of SHIELD, a GAT model designed to infer biologically meaningful cell-cell interactions, and second, demonstrate its capability in two applications, i.e., by identifying weak and rare interactions between mucosal-associated invariant T (MAIT) cells and macrophages within the context of HCC and by uncovering interactions associated with β cell depletion mediated by T cytotoxic and T helper cells in T1D.

### Cell-cell interaction inference with SHIELD

This section offers a high-level overview of the model; a full description of the architecture and training procedure is provided in the methods section.

**Figure 1:**
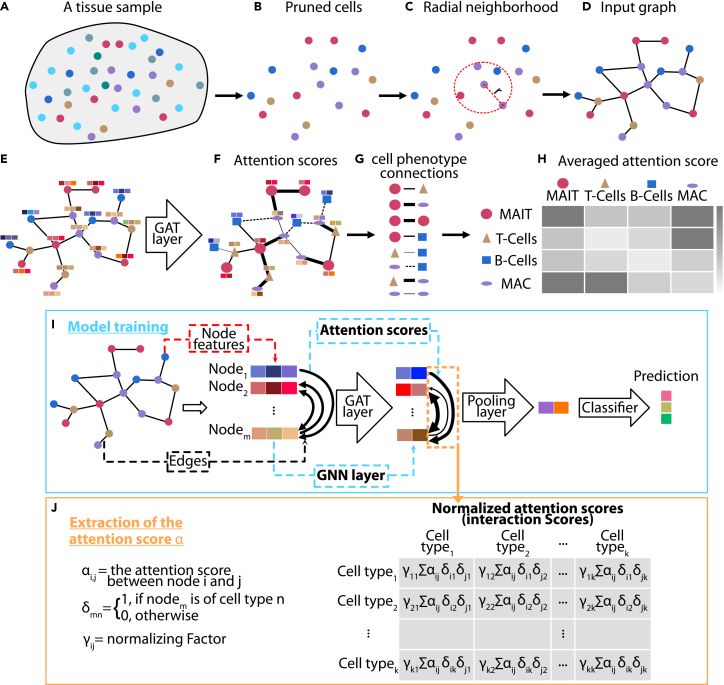
Overview of SHIELD: From high-dimensional spatial proteomic data to graph-based input and interpretable attention scores (A) Cartoon of a segmented tissue sample. Each circle represents a cell with unique node features, such as an expression profile. The color of the circles represents cell type (node labels). (B) For specific classification tasks, only a subset of cells of interest is considered (e.g., non-immune cells). (C and D) A graph is constructed by connecting cells within a defined radial neighborhood (*r*) of each other. (E) Each node is annotated by its features (boxes above the nodes). Color represents the cell type (node label). The resulting graph is the input for the graph attention network. (F) GAT layer updates the node features (dimensionality and value) and calculates attention scores for each edge. Changes in the feature dimensionality and values are reflected in the transformed shapes and colors of the node features. The attention scores modify the edge weights, illustrated by varying edge thickness and dotted patterns. (G and H) SHIELD reports interpretable interaction scores for cell-type pairs by summarizing attention scores. (I) Comprehensive depiction of the GAT architecture: GAT layer uses graph-based message passing to transform node features and compute attention scores, weighing feature relevance for downstream tasks. A mean pooling layer integrates node-level information to predict graph-level labels, which are used to optimize the model via loss functions. (J) To calculate the interaction score, the attention scores are aggregated for all edges connecting nodes of specific cell types. These scores are normalized by *γ*_*k*,*l*_ on the total number of connections to each cell type (Equation 7), yielding cell-type-specific interaction metrics, as described in Equation 8.

SHIELD systematically infers interpretable cell-cell interaction from multiplexed tissue imaging data. SHIELD quantifies interaction strengths and extracts biologically meaningful cell-cell interaction relationships in a data-driven fashion by considering a supervised prediction task, such as association with disease state. SHIELD is a graph attention model that implements a self-attention mechanism encoded by attention scores. The model is trained on spatial graphs, where nodes represent individual cells, annotated with their expression profiles, and edges encode spatial proximity. Once trained, the extracted attention scores are correlated with respect to their cell type. This result provides a hierarchy of putative cell type-cell type interactions, which in turn offer mechanistic hypotheses into tissue organization conferring the disease phenotype of interest (Figure 1).

For graph construction, we designed two sampling schemes for the selection of cells. The first is the “bucket” scheme, where we fix the number of buckets (*b*) and randomly distribute all cells within the region of interest (ROI) into these buckets. This defines how many samples are generated per tissue rather than specifying a fixed number of cells per sample. This approach ensures that each patient contributes an equal number of samples, avoiding potential bias toward patients with higher cell counts. Each cell is assigned to only one bucket and sampled only once. The second strategy is based on Voronoi-based compartmentalization, which preserves spatial organization while ensuring sufficiently large and diverse training and testing datasets. To mitigate edge effects at compartment borders, we introduced a fuzzy border parameter, *f*_*border*_, allowing cells near compartment boundaries to be assigned to multiple compartments. The degree of overlap was treated as a tunable hyperparameter and optimized during hyperparameter search. Depending on model performance, either the bucket-based or the Voronoi-based strategy was selected for final evaluation.

SHIELD is trained in a weakly supervised learning manner, where the model is optimized on a classification task (e.g., tissue region or patient label) and does not access node labels during the training. While classification accuracy is an important goal, extracting specific, and thereby interpretable, cell-cell interactions is the primary objective. After training, attention scores are correlated with node labels (Figure 1G) and averaged across all samples of the same graph label to reveal cell-type-specific interactions (Figure 1H) (interaction scores). To that end, the attention scores of the trained model are summarized post hoc and presented as interaction scores to reveal which pairwise cell-type interactions are most predictive for the classification label. This scoring accomplishes the goal of achieving specific cell type-cell type interactions related to the disease phenotype of interest.

### SHIELD identifies rare liver cancer-associated interactions of MAIT cells

MAIT cells have recently been identified as outcome-associated immune cells in HCC and have been experimentally validated within a comprehensive mouse model by Ruf et al.^25^ Here, we utilize SHIELD to recover and identify cell-cell interactions.

**Figure 2:**
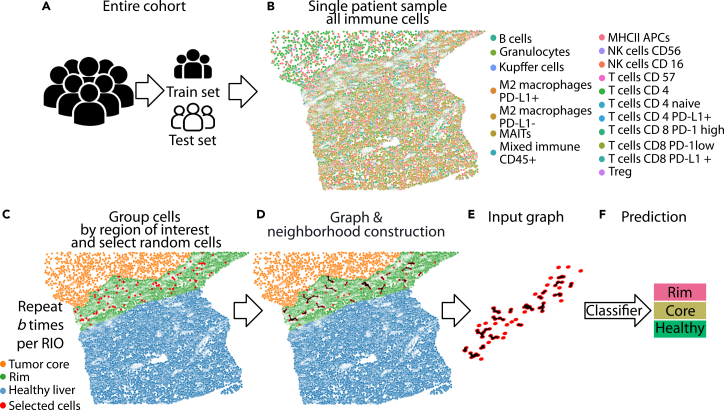
Construction of spatial graphs for multiplexed tissue images of HCC (A) The dataset includes liver tissue samples from 15 patients diagnosed with hepatocellular carcinoma (HCC). Patients were randomly split into a training cohort (10 patients) and a test cohort (5 patients). (B) Representative CODEX image showing all CD45^+^ immune cell types, with corresponding cell-type legend. (C) Cells colored by region of interest (ROI): healthy liver, rim, or tumor core. Cells highlighted in red represent one randomly sampled “bucket” used in the bucket-based sampling scheme. (D) Spatial graph showing radial connectivity between cells (black edges), constructed using a fixed neighborhood radius of *r* = 200 μm. (E) An input graph used for the classification task. (F) The resulting graph is passed to a graph attention network (GAT) with one hidden layer, which outputs a prediction corresponding to the ROI class (healthy, rim, or tumor core).

The dataset, originally processed by Ruf et al.,^25^ consists of liver tissue samples from 15 male patients diagnosed with HCC. Experts annotated each sample into three distinct regions: healthy liver, rim, and tumor core. The dataset comprises approximately 1.5 million segmented cells, each characterized by 27 protein markers indicative of immune and stromal cell types (Figures 2A and 2B). We ensured a robust split into training and test cohorts by assigning 10 patients to the training cohort and 5 patients to the test cohort.

We applied the bucket-based sampling scheme for spatial graph construction in the HCC dataset. For each ROI within the multiplexed tissue images, cells were randomly distributed into 500 buckets, creating spatially constrained compartments without relying on predefined anchor points. This sampling procedure was repeated five times to increase cohort size and enhance sample diversity, resulting in a total of 24,900 samples.

We aimed to study the relationship between local immune cell composition and HCC by classifying tissue regions into healthy liver, rim, and tumor core. To this end, we restricted our analysis to CD45^+^ immune cells. Edges were constructed by connecting each cell to its nearest neighbors within a radial distance of *r* = 200 μm (approximately 530 pixels).

The number of buckets (*b*) and the neighborhood radius (*r*) were determined via hyperparameter optimization using a 3-fold cross-validation scheme. The results of this search are shown in Figure S3. For the HCC dataset, the Voronoi tessellation strategy resulted in noticeably lower overall accuracy and showed pronounced difficulties in distinguishing between healthy and core regions (Figures S20–S23).

We applied SHIELD to the HCC dataset to systematically analyze immune cell interactions without relying on predefined cell-type pairs. Although the underlying spatial graph is undirected, the attention-based interaction score is inherently directional: each score reflects how strongly a source cell type (i.e., the node being classified) attends to its neighbors. This allows asymmetric interaction modeling, where—for example—macrophages may be highly informative for MAIT cells but not necessarily vice versa. Such asymmetries are biologically plausible, as cell-cell communication often follows unidirectional or context-dependent pathways.

**Figure 3:**
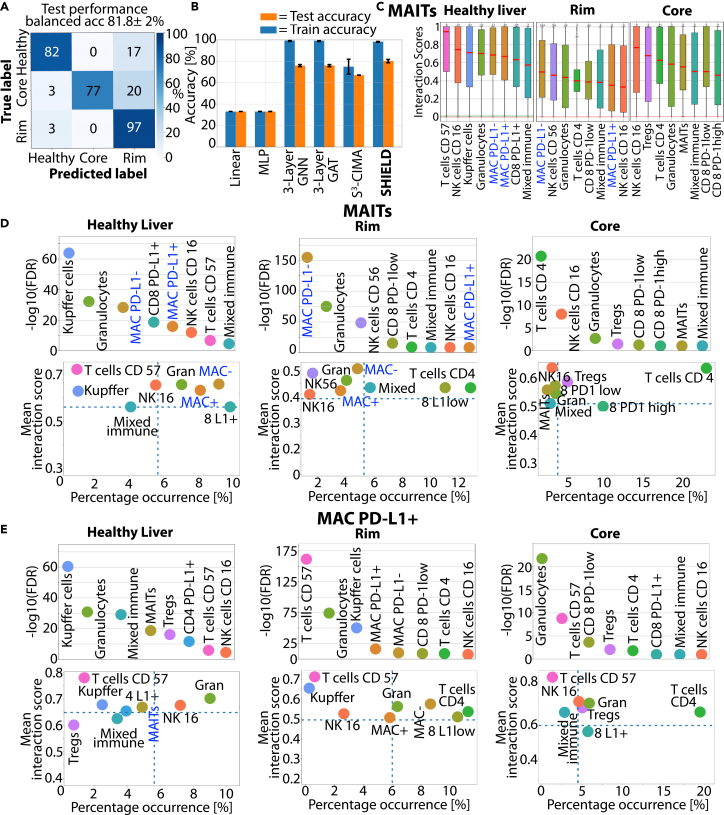
Classification performance and directional interaction analysis in hepatocellular carcinoma (A) Confusion matrix showing SHIELD’s test performance in classifying tissue compartments as healthy liver, rim, or tumor core. Most confusion occurs at biological transition zones, particularly between the rim and core. (B) Comparison of classification accuracy between SHIELD and several baseline models, including S^3^-CIMA, multi-layer GNNs, MLPs, and linear classifiers. SHIELD outperforms the larger models while maintaining interpretability through a single-layer GAT architecture. The error bars denote the standard error of the mean across runs. (C) Boxplots showing the distribution of the top 8 interaction scores for MAIT cells, stratified by region of interest. The red dashed line denotes the median score across all interactions, and green bands mark the interquartile range. Notably, interactions with both PD-L1^+^ and PD-L1^–^ macrophages in the healthy and rim regions are highlighted in blue, indicating cell-type-relevant interactions despite their low spatial abundance. (D) Top eight interaction partners for MAIT cells, ranked by −*log*_10_(FDR)-corrected Mann-Whitney U test values. Below, the nearest-neighbor (NN) correlation plots show the relationship between the interaction score and spatial co-occurrence. MACs appear in the upper-left quadrant—statistically enriched interactions that are spatially rare, underscoring SHIELD’s ability to capture non-obvious, immune-relevant relationships. (E) Same analysis as in (D) but using PD-L1^+^ macrophages (MACs) as the source cell type. MAITs emerge as a top-ranked target interaction in the healthy region, providing evidence for reciprocal, directional immune communication that would be missed using proximity-based metrics alone.

To quantify cell-type-specific interactions, we evaluated the interaction score (Equation 8) alongside −*log*_10_(FDR)-corrected significance testing and a nearest-neighbor (NN) control analysis (Figure 3). The NN analysis plotted the mean interaction score against average spatial proximity rank, helping identify rare but biologically relevant signals. Focusing on MAIT cells as the source population (Figures 3C–3E), we confirmed and expanded upon the findings of Ruf et al.^25^ In both the healthy liver and rim regions, SHIELD highlighted significant interactions between MAIT cells and PD-L1^+^ and PD-L1^–^ macrophages (MACs^+^ and MACs^–^). These interactions were highly significant according to false discovery rate (FDR)-corrected Mann-Whitney U tests and occurred in the top-left quadrant of the NN control plot—indicating for the rim ROI that although these interactions are spatially rare, SHIELD assigns them high biological relevance. This supports the hypothesis that MAIT cells are downregulated by macrophages as part of the immune response, particularly in the rim, where immune surveillance is active but tumor invasion is ongoing. Conversely, when considering MAC^+^ cells as the source node (Figures 3F–3H), SHIELD recovered strong interactions with MAIT cells predominantly in the healthy region. This reciprocal yet spatially constrained signal reinforces the biological relevance of MAIT-macrophage crosstalk in immune homeostasis and tumor-edge containment. These interactions, again found in the upper-left quadrant of the NN analysis, are rare but consistent with prior knowledge of macrophage-mediated immunomodulation. Together, these results demonstrate SHIELD’s ability to uncover both known and directed interactions, even when they are not prevalent in the spatial neighborhood—underscoring its utility for interpretable, context-aware immune interaction discovery. SHIELD also identified a previously unrecognized strong interaction of MAIT cells with granulocytes. Additionally, while the original study suggested further investigation into CD8 T cell interactions, SHIELD independently revealed these relationships without additional analysis (Figure 3E), uncovering putative interactions between regulatory T cells (T_reg_ cells), granulocytes, and CD8 T cells.

We compared SHIELD against multiple baseline models, including a three-layer GAT, a GNN, a multi-layer perceptron (MLP), and linear classifiers. Typically, larger graph models are expected to achieve better performance on the training set; thus, it is notable that our compact model outperformed these baselines. A possible explanation for this unexpected result could be that exhaustive hyperparameter evaluation is practically difficult for the larger models. SHIELD demonstrated superior accuracy, particularly in distinguishing the well-defined tumor core. Misclassifications between the rim and healthy liver regions were expected, as the expert annotations in these transition zones lack quantitative definitions. In contrast, deeper GNN and GAT models showed weaker generalization, while non-graph-based models (MLP and linear) failed to capture spatial relationships, reinforcing the necessity of graph-based methods.

Importantly, SHIELD also provides advantages over simple NN analysis, which evaluates co-localization frequency or average proximity across cell types. While NN-based metrics can highlight frequent spatial co-occurrence, they are agnostic to phenotype labels and fail to quantify whether a given interaction is relevant to a specific tissue state or classification objective. SHIELD overcomes this limitation by learning attention weights in a supervised fashion—assigning high interaction scores only when a neighboring cell type contributes meaningfully to phenotype prediction. This makes the interaction score both directional and context aware, capturing cell-type-coupled interaction relevance rather than mere spatial proximity. As demonstrated in the MAIT-macrophage analysis (Figure 3), several significant interactions lie in the upper-left quadrant of the NN control plot—indicating that SHIELD detects rare but highly informative spatial relationships that would be overlooked by frequency-based approaches. Thus, SHIELD provides a principled, interpretable mechanism to prioritize biologically meaningful cell-cell communication beyond co-localization. All interaction scores, FDR-corrected significance values, and NN correlation plots are provided in Figures S13–S19.

To validate the biological relevance of SHIELD’s predictions, we introduced a NN control analysis that compares the learned interaction scores against spatial co-occurrence frequencies. Across all disease contexts, SHIELD consistently identified significant interactions that are rare in the tissue—with average co-occurrence rates below 8% for many key pairs. This demonstrates that SHIELD goes beyond co-localization, uncovering sparse but functionally important interactions that would be missed by traditional frequency- or proximity-based analyses. The directionality and supervised nature of the attention mechanism allow SHIELD to model cell-type-specific relationships, distinguishing it from unsupervised neighborhood enrichment approaches.

To further assess SHIELD’s robustness, we conducted ablation experiments (Figure S2): introducing spatial perturbations through edge shuffling (both degree-preserving and random), probabilistic and rule-based label noise (Figures S10–S12 and S5), and selective downsampling of key immune populations such as PD-L1^+^ macrophages (Figure S7–S9). In all scenarios, SHIELD maintained high classification performance and recovered meaningful interactions, supporting its stability against noise and bias in both input features and graph structure.

In addition to the above application of SHIELD, we include an analysis of CRC tissues, also measured by CODEX (Figure S1). Here, SHIELD recovered a notable interaction between CD8^+^ T cells and CD11b^+^CD68^+^ macrophages, enriched in poor responders (Figure S2). This pair was not explicitly reported in the original analysis by Schürch et al.^10^ but aligns with their identification of coupled cellular neighborhoods (CN-1 and CN-4), which were enriched in CD8^+^ T cells and macrophages, respectively. The suppressive nature of this interaction was further supported by canonical correlation analyses in that study, where proliferating CD8^+^ T cells were negatively correlated with Treg cells and CD163^+^ macrophages. SHIELD not only recovered this suppressive axis in a fully supervised setting but also made it interpretable as a direct, quantifiable cell-cell interaction ranked among the top contributors to response classification. Compared to S^3^-CIMA, which identified indirect local enrichment of PD-1^+^CD4^+^ T cells within granulocyte neighborhoods, SHIELD’s graph-based framework directly surfaces pairwise immune interactions, highlighting a macrophage-mediated immunosuppressive circuit that may underlie therapy resistance in non-responders. This finding exemplifies SHIELD’s capacity to reconstruct and refine biological mechanisms described in unsupervised frameworks while offering greater interpretability and specificity. All interaction scores, FDR-corrected significance values, and NN correlation plots are provided in Figures S31–S35. We also evaluated bucket sampling, which showed significantly worse test performance on the CRC dataset; all results can be found in Figures S36–S39.

In conclusion, SHIELD detected tissue-specific immune cell interactions, distinguishing differential interactions from frequent ones in a data-driven manner. Importantly, SHIELD replicated key findings from previous studies^24^ without explicit cell-type selection, highlighting its capability for systematic, unbiased discovery.

### Detection of β cell depletion by T helper and cytotoxic T cells

T1D is characterized by immune-mediated β cell depletion, yet the precise dynamics of ultimately deleterious interactions in these cells remain only partially understood. While previous studies have investigated T cell-driven β cell depletion using neighborhood analyses, these approaches required predefined selection criteria and expert-driven feature extraction.^12^ Here, we applied SHIELD to systematically infer immune interactions from imaging mass cytometry data of this study, allowing for unbiased detection of β cell interactions across the considered disease states.

The data comprised pancreatic tissue samples from three patient cohorts representing the disease states onset T1D (<0.5 years, *N* = 4), long-duration T1D (≤8 years, *N* = 4), and non-diabetic controls (*N* = 4). Each patient’s data included multiple imaging regions (tail, body, and head) of the pancreas, providing a larger dataset than previous studies. This enabled a robust analysis of immune-mediated β cell depletion across different disease stages.

**Figure 4:**
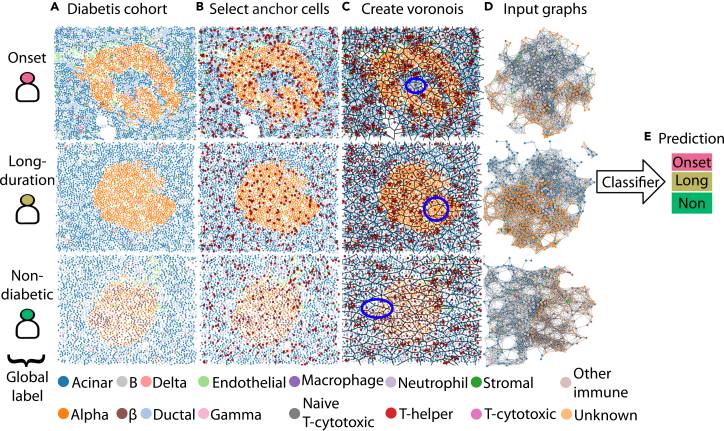
Construction of spatial graphs for pancreas imaging mass cytometry data for diabetes cohorts (A) Representative images of patients for each disease label (onset, long duration, and non-diabetic). (B) Random selection (20%) of Voronoi center cells (red) as seed points for the Voronoi compartments. (C) Voronoi tessellation center cells with a fuzzy border parameter of *f*_*border*_ = 0.8. (D) Spatial graphs created from the Voronoi partitions. Nodes represent cell types (the legend is provided underneath), and edges capture spatial relationships with a radial radius of *r* = 27 pixels. (E) One-layer graph attention network, mean pooling layer, and linear classifier trained to predict global labels (onset, long duration, and non-diabetic).

To generate spatial graphs, we considered Voronoi compartments based on 20% of all cells per image (see methods section). As immune and structural cells were considered potentially relevant for T1D progression, all cells in the dataset were considered for further analysis (Figures 4A–E).

The dataset was split into four subsets, where different patients were included within the train or validation set, with three used for hyperparameter optimization and validation and the fourth held out as an independent test set. The hyperparameter search, a 3-fold cross-validation, revealed that choosing a large number of Voronoi center cells, i.e., 20% of all cells contained in an image, a fuzzy border of *f*_*border*_ = 0.8, and a radial radius of *r* = 27 pixels, yielded optimal results while mitigating overfitting (Figure S2B). The final dataset comprised 3,249 training and 1,082 test graphs, balanced across all three disease labels.

We also evaluated the bucket sampling strategy but found that the model did not generalize as well, particularly struggling to differentiate between non-diabetic and long-term diabetic patients (Figures S27–S30).

**Figure 5:**
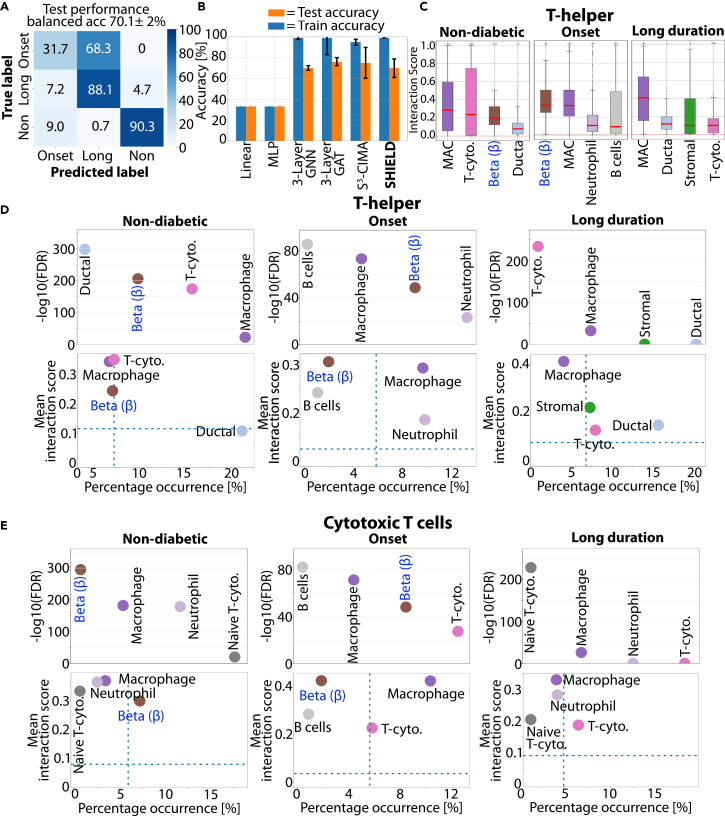
SHIELD identifies cell-type-relevant immune interactions in type 1 diabetes (A) Confusion matrix showing SHIELD’s test performance in classifying tissue regions across non-diabetic, onset, and long-duration type 1 diabetes (T1D) stages. Misclassifications primarily occur between non-diabetic and long-duration tissues, consistent with the gradual, heterogeneous nature of immune infiltration. (B) Comparison of classification accuracy between SHIELD and several baseline models, including S^3^-CIMA, multi-layer GNNs, MLPs, and linear classifiers. SHIELD achieves competitive accuracy while maintaining interpretability through a single-layer graph attention architecture. The error bars denote the standard error of the mean across runs. (C) Boxplots showing interaction scores for T helper cells with their top four interacting partners across all three disease stages. Red dashed lines indicate the median interaction score across all partners; green bands denote the interquartile range. (D) Significance and spatial context of T helper cell interactions. The top eight interaction partners are ranked by −*log*_10_(FDR)-corrected Mann-Whitney U test. Below, the nearest-neighbor (NN) correlation plots compare the interaction score (*y* axis) with spatial co-occurrence (*x* axis). A key finding is highlighted in blue: β cells form a statistically significant but spatially rare interaction with T helper cells, revealed by SHIELD’s directed attention mechanism. (E) Same analysis as in (D), repeated for cytotoxic T cells (T-Cyto). SHIELD identifies β cell*-*targeting interactions that are both stage specific and rare in spatial proximity—illustrating the model’s ability to uncover early autoimmune signatures not detectable through neighborhood analysis alone.

SHIELD effectively uncovers stage-specific immune interactions that drive β cell depletion in T1D (Figures 5C–5E). By leveraging attention-based interaction scores, SHIELD pinpoints the cellular crosstalk between β cells and T cell subtypes across disease stages, insights that elude conventional neighborhood-based analyses such as those used as a baseline in Babaei et al.^24^

In non-diabetic samples (Figure 5C), SHIELD reveals robust interactions between β cells and multiple T cell subtypes, including naive cytotoxic T cells (CD8^+^), mature cytotoxic T cells, and helper T cells (CD4^+^), reflecting the immune surveillance landscape in healthy islets. These findings suggest that even in non-pathological conditions, there is substantial immune-β cell crosstalk. Importantly, these interactions were not only highly significant based on the interaction scores but also occurred at very low spatial co-occurrence frequencies, as confirmed by the NN control analysis. Specifically, the average spatial proximity of these interacting cell types was below 8%, indicating that SHIELD uncovered functionally relevant interactions that would have been missed by conventional proximity- or frequency-based analyses. This highlights SHIELD’s capacity to detect relevant cell type-cell type interactions that are sparse but predictive—revealing biologically meaningful relationships beyond what is measurable from spatial proximity alone.

In the onset T1D cohort (Figure 5D), SHIELD detects a marked amplification of interaction scores between β cells and both cytotoxic and helper T cells. This elevated immune-β cell interaction signal corresponds with the known early immune assault on pancreatic islets—a hallmark of T1D pathogenesis and a key observation previously reported in Damond et al.^12^ Beyond statistical significance, these interactions were also rare in terms of spatial proximity: the NN control analysis revealed that the average co-occurrence of these T cell types near β cells was below 2%. This indicates that SHIELD uncovered highly cell-type-relevant immune interactions that are not detectable through frequency-based or spatially naive methods. Together, these results demonstrate that SHIELD captures the onset-stage immune activation not only more sensitively but also more specifically, identifying sparse but predictive interactions that characterize early T1D.

In contrast, the long-duration T1D cohort (Figure 5E) exhibits a complete absence of β cell interactions, reflecting the near-total depletion of β cells at advanced disease stages. This transition, from high-scoring, specific immune interactions at onset to their total absence in late-stage disease, is quantitatively captured by SHIELD and aligns with expected pathological progression.

In summary, SHIELD dissects the contribution of each immune interaction in a stage-resolved manner. In both non-diabetic and onset cohorts, SHIELD highlights the importance of early interactions between β cells and naive CD8^+^ and CD4^+^ T cells, suggesting their priming role in initiating autoimmune responses. This level of mechanistic resolution is not evident in static cell proximity analyses and demonstrates SHIELD’s potential to uncover subtle yet critical cellular interactions shaping disease evolution. The model exhibited high classification performance. We observed confusion between the onset and long-duration groups, which is biologically plausible given the continuum of disease progression across individuals.

We compared it to multiple baseline approaches, and graph-based baselines exhibited significant overfitting, achieving near-perfect training accuracy while showing significantly worse test accuracy (Table S4). Non-graph-based models (MLP and linear) performed worse on both training and test data, highlighting the necessity of incorporating spatial relationships in immune cell interaction modeling. SHIELD matched or exceeded baseline accuracy while allowing the emphasis-specific cell-cell interaction pairs via our interaction scores. The lack of interpretability in multi-layer GNNs and GATs emphasizes the advantage of SHIELD’s simpler yet more transparent architecture in resolving immune-driven β cell depletion.

Our approach enables both the validation of established biological mechanisms and the discovery of previously unrecognized cellular interactions (Figures 5B–5D). Unlike prior methods that rely on predefined neighborhood analyses or target-specific cell types, such as S^3^-CIMA, SHIELD infers interactions directly from the data, eliminating selection biases that may arise from focusing on specific cellular relationships, such as the β-T helper cell interaction. By considering all possible cell-cell interactions equally, our method ensures that overlooked drivers of β cell depletion can be identified. Notably, S^3^-CIMA was unable to resolve interactions between naive T cytotoxic and T cytotoxic cells with β cells or detect significant T helper-β cell interactions, both of which have been implicated as key contributors to β cell depletion in T1D.^12^ SHIELD is a data-driven approach overcoming these limitations, providing a more comprehensive view of the immune landscape driving disease progression. By systematically analyzing the interaction scores, SHIELD provides a scalable and interpretable approach for studying spatially resolved immune responses in T1D, in particular, detecting cell types whose proximity to β cell*s* is associated with T1D progression. All interaction scores, FDR-corrected significance values, and NN correlation plots are provided in Figures S24–S26.

## Discussion

We present SHIELD, a model designed to associate multiplexed tissue imaging data and macroscopic cell types. By leveraging the attention mechanism of the model, SHIELD allows for the definition of putative cell type-cell type interactions that confer the phenotype of interest. The SHIELD model is deliberately designed for simplicity, enabling interpretability while maintaining strong predictive capabilities.

We compared our results primarily with S^3^-CIMA^24^ because, to our knowledge, there are no other models that capture cell-cell interactions in such a fashion. Unlike SpatialGlue,^20^ which uses graph encoders to align multi-omics data with spatial measurements without explicitly quantifying cell-cell interactions, SHIELD directly assesses these interactions, capturing biologically relevant mechanisms. SpatialGlue^20^ reconstructs spatial graphs; it does not identify interactions that drive a biological response or are necessary for predictions. Similarly, scHolography^21^ reconstructs 2D spatial omics into 3D grids to map tissue-level patterns but provides no insights into how specific cell interactions influence immune responses. CytoCommunity,^26^ the closest candidate for a baseline, identifies cellular communities through localized neighborhood analysis in spatial graphs, potentially highlighting immune cells involved in responses. While one could train classifiers on these communities to interpret marker-level roles, this approach only identifies groups of immune cells, not the specific hierarchical cell-cell interactions critical for the immune response. Due to these fundamental differences, we explicitly chose not to use these methods as baselines. SHIELD uniquely addresses the need to investigate immune responses depending on specific interactions among individual cells.

Notably, unlike ligand-receptor-based models,^22^^,^^23^ SHIELD does not rely on curated molecular priors or signaling databases and is therefore not limited by the experimental design. This makes it applicable across a wide range of spatial omics platforms, including those that lack genome-wide resolution, such as imaging mass cytometry or CODEX.

SHIELD is designed for flexibility, allowing user-defined parameters such as the radii for graph sampling. These parameters can be tailored based on expert-derived expectations or determined empirically from the data, making SHIELD adaptable to diverse applications.

A key innovation of SHIELD lies in the interpretation of its attention mechanisms. Interestingly, the attention scores yielded non-symmetric relationships across all datasets. This asymmetry arises from two factors: (1) the attention function, defined in Equation 3, uses a feedforward neural network *a* that processes node features asymmetrically. While *a*(*n*_1_,*n*_2_) could enforce commutative properties, we deliberately chose not to, allowing the model to capture the natural directionality of cell interactions. (2) The softmax normalization in Equation 5 creates directed attention scores by normalizing each score relative to the source node’s connections. Biologically, this asymmetry reflects one-sided interactions, such as T_reg_ cells suppressing immune responses in HCC or cytotoxic T cells targeting β cells in T1D.

SHIELD has limitations—by aggregating attention scores post hoc across all edges between cell-type pairs, we derive interaction scores that quantify which cell type-cell type co-localizations are predictive of the phenotype. We note, however, that these scores do not imply functional or causal relationships and, in particular, do not imply mechanistic cell-cell communication. Rather, the resulting interaction scores should be interpreted as hypothesis-generating signals—highlighting spatially enriched, cell-type-associated interactions that warrant further experimental or orthogonal validation. While our model effectively identifies key interaction scores, it is constrained by the resolution of the input data; interactions occurring at subcellular scales or across extended tissue regions may not be fully captured. One potential mitigation strategy is the incorporation of hierarchical graph representations that integrate multi-scale spatial information. While our single-layer GAT prioritizes interpretability, more complex multi-layer architectures could further improve performance at the cost of increased computational efficiency and interpretability. To further characterize SHIELD’s sensitivity limits, we performed an extreme population ablation analysis on the HCC dataset, progressively reducing the MAC population to the absolute frequencies of 1.66% (relative frequency 16%), 0.83% (8%), and 0.2% (2%). At 0.83% and 0.2% frequencies, the tumor region no longer exhibited detectable MAC^−^ interactions, indicating the lower boundary at which reliable detection is lost. In contrast, the healthy region consistently identified MAIT cells and both MAC^+^/MAC^−^ populations as key interacting partners, with interaction strengths even increasing, most likely due to a distribution shift emphasizing differences between the tumor core and healthy liver. However, at a 0.2% absolute MAC frequency, the rim region no longer showed significant interactions between the MAITs and MACs^+^/MACs^−^, marking the effective detection limit of SHIELD for this dataset (Table S5; Figure S6). Future iterations of SHIELD could explore hybrid models that balance interpretability with predictive power, ensuring broad applicability in spatial biology research.

As SHIELD is ultimately a classification model from which interaction scores are derived post hoc, the reliability of these scores inherently depends on the model’s classification performance. Interaction scores are expected to become less reliable, particularly those used to discriminate across the classification labels as predictive accuracy decreases, e.g., due to increased task complexity or data noise. To provide a practical guideline, we define a minimum reliability threshold of $1-\frac{1}{\text{number}\;\text{of}\;\text{classes}}$; for the HCC dataset with three classes (healthy, rim, and tumor), this corresponds to an approximate range of ≈64%–66%. Below this level, class discrimination becomes unreliable, and the resulting interaction scores should be interpreted with caution. We emphasize that this threshold is a heuristic guideline rather than a formal theoretical bound; it is intended to provide users with a conservative baseline for assessing when interaction scores are likely to be informative. In our experiments, SHIELD achieved a balanced accuracy of about 80%, and even under the strongest perturbations, performance did not drop below 65%, which we consider the lower limit of interpretability.

At this stage, our analysis primarily focuses on attention scores as the key mechanism for interpretability. However, SHIELD’s model structure includes two additional sources of trained weights: the graph convolution layer and the classification layer, both of which encode valuable information about the learned representations of cellular interactions. A systematic investigation of these layers could provide more insights into how different cell types contribute to classification decisions and whether additional biologically meaningful patterns are embedded in these weight matrices. Future work will aim at incorporating these elements into the interpretable machine learning approach.

In conclusion, SHIELD provides an effective approach to associating multiplexed tissue imaging data with macroscopic cell types. By enabling interpretable, comprehensive, and supervised identification of cell-cell interactions, without relying on molecular priors or local spatial proximity, it offers a principled framework for decoding immune responses in health and disease. We expect this model to contribute and catalyze future biological and translational studies utilizing multiplexed tissue imaging data.

## Methods

### Graph creation

For each sample, we constructed spatial graphs using one of two sampling schemes designed to preserve tissue structure while supporting model interpretability and statistical robustness: a bucket-based random sampling strategy and a Voronoi-based compartmentalization approach. The choice of sampling method was dataset specific and determined through performance-based model selection.

The first strategy is the bucket scheme, where we fix the number of buckets (*b*) and randomly distribute all cells within the ROI or image-based label into these buckets. This determines how many graph samples are generated per label rather than fixing the number of cells per graph. The number of cells per bucket is kept approximately constant, with minor variation in a few buckets to ensure complete coverage and consistency. Each cell is assigned to exactly one bucket and sampled only once. This approach ensures that each patient contributes an equal number of graphs per label, thereby avoiding bias toward patients with higher cell counts and supporting better generalization across the cohort. Notably, while the number of graphs per label is fixed, the number of nodes (cells) per graph is not—due to natural variation in cell abundance across patients and tissue regions, individual buckets will contain different numbers of cells depending on the label and patient.

The second strategy is based on Voronoi tessellation. A set of randomly selected Voronoi center cells is chosen to define Voronoi compartments (Figure 1B). Each cell in the image is then assigned to the nearest Voronoi center cell, forming distinct spatial compartments. To prevent biologically relevant clusters near compartment boundaries from being artificially split across graphs, we introduced a fuzzy border parameter *f*_*border*_, which allowed cells near the boundary to belong to multiple compartments. A cell initially assigned to Voronoi compartment *v*_*i*_ is also assigned to *v*_*j*_ if the following condition is met:

(Equation 1) $$\frac{d_{i}}{d_{j}}>f_{border}\text{,}$$

where *d*_*i*_ and *d*_*j*_ represent the distances between the cell and the Voronoi center cells of compartments *v*_*i*_ and *v*_*j*_, respectively (Figure 1C). This mechanism allows for partial membership across compartment boundaries and preserves important spatial context. The fuzzy border parameter was treated as a tunable hyperparameter and optimized during model selection.

For both strategies, each graph node corresponds to a single cell and is annotated with that cell’s expression profile, forming the node’s feature vector (Figure 1B). These features capture biologically meaningful molecular signals, such as gene or protein expression. Edges were defined based on spatial proximity: two cells were connected if their Euclidean distance was below a predefined radial threshold *r*. This threshold, a key hyperparameter, determines the spatial scale of interaction encoded in the graph and ensures that only biologically plausible local neighborhoods are connected (Figure 1C).

Several hyperparameters controlled graph construction, including the fuzzy border parameter (*f*_*border*_), the edge radius (*r*), the number of Voronoi compartments or buckets (*b*), and the minimum number of cells per compartment to avoid underpopulated graphs. These were optimized through a k-fold cross-validation hyperparameter search, ensuring biologically meaningful spatial context and sufficient predictive power. Depending on performance, either the bucket-based or Voronoi-based strategy was used for final model training and evaluation.

### Model

SHIELD allows for classification and the desired interpretability by evaluating attention scores. We propose an edge attention score summarization that enables us to identify cell-type interactions in the tissue that confer the model’s classification capability.

We used a one-layer GAT^27^^,^^28^ model that received the cell expression profile and position data matrix derived from cell imaging data represented as a graph.

The GAT model uses these graphs as input to define and learn interaction-associated features from the node representations, i.e., attention scores, an element used for later classification (Equation 4). This focus on attention scores distinguishes this model from conventional graph convolution^29^ classification models that base classification only on individual node features and message parsing by treating every edge as equally important.

The node features were transformed through a learnable weight matrix *W,* which was optimized by the node features ${\vec{h}}_{i}$ interaction-associated features as $W{\vec{h}}_{i}$, thus transforming the features from $R^{F}$ to $R^{F^{\prime }}$ (Figure 1F). Subsequently, attention coefficients were calculated from these as follows:

(Equation 2) $$\alpha_{ij}=\text{softmax}\left(e_{ij}\right)\text{,}$$

(Equation 3) $$=\frac{\;\exp\;\left(e\left(h_{i},h_{j}\right)\right)}{\sum_{j^{\prime }\in N_{i}}\;\exp\;\left(e\left(h_{i},h_{j^{\prime }}\right)\right)}\text{,}$$

where $e_{ij}=a\left(W{\vec{h}}_{i},W{\vec{h}}_{j}\right)$ denotes the attention value for a connection between nodes *h*_*i*_ and *h*_*j*_ and $N_{i}$ represents the set of all nodes connected to node *h*_*i*_ (Figure 1G). For a given node *h*_*i*_, all attention scores were aggregated and passed through a nonlinear activation function *σ*:

(Equation 4) $${\vec{h}}_{i}^{\prime }=\sigma \left(\sum_{j\in N_{i}}\alpha_{ij}W{\vec{h}}_{j}\right)\text{.}$$

The adjacency matrix is utilized for masking, thereby preserving the graph structure and message passing.^28^

In our study, we designed a graph prediction task using the previously mentioned input graphs (Figure 1E). Our network architecture comprised a single GAT layer, a global mean pooling layer, and a linear classifier layer (Figure 1I). The classification output was passed through a softmax function, categorizing it into discrete labels. The pooling layer served a dual purpose: it facilitated graph-based prediction by aggregating information across nodes and accommodated graphs of variable sizes. This flexibility was important for our approach, as it avoided bias related to the cell count in different subregions of the images.

Given the task of differentiating distinct classes, we used the cross-entropy loss function for our model.

Additionally, we chose to omit self-attention mechanisms in our analysis. This decision was made after observing that the attention scores disproportionately favored self-connections, which diminished the network’s interpretative value by overshadowing other potentially informative interactions.

All models were trained utilizing a GeForce GTX 2080 Ti graphics card. All hyperparameters were optimized using a validation set, thus slicing the training dataset into multiple validation training sets. No hyperparameter optimization was done on the test set.

### Attention evaluation

Post-training, we reconfigured the graph structure to transform the attention scores into an interpretable model by grouping the nodes into their respective cell type. i.e., the node label. This allowed us to map the cell-to-cell attention scores onto a more meaningful cell type-to-cell type attention matrix. It is crucial to highlight that the model does not receive cell-type information (node label) during any part of training or testing, nor are the hyperparameters tuned based on this correlation. Instead, all training-related features were optimized solely on the graphs, where node features represent protein or gene expression profiles and edges define spatial neighborhoods. Cell-type correlations were explored only during post-training interpretation by extracting attention scores to identify which cell interactions were the most significant. This approach enabled the creation of an interpretable model that provided insights into the decision-making process of the data-driven deep-learning approach. This paper’s key contribution is unraveling these interactions and offering an interpretable explanation of the model’s predictions.

The newly defined attention score $A\left(c_{k},c_{l}\right)$ represents the sum of all attention scores *α*_*ij*_, where node *h*_*i*_ is of cell type *c*_*k*_ and node *h*_*j*_ is of cell type *c*_*l*_:

(Equation 5) $$A\left(c_{k},c_{l}\right)=\sum_{i,j}\alpha_{ij}\delta_{ik}\delta_{jl},\text{with}$$

(Equation 6) $$\delta_{mn}=\left\{ \begin{array}{cc} 1, & \text{if}\;{\text{node}}_{m}\;\text{is}\;\text{of}\;\text{cell}\;\text{type}\;n,\\ 0, & \text{otherwise}. \end{array}\right.$$

Equation 6 ensures that we sum the attention values specific to an interaction between designated cell types (Figure 1H). To adjust for patient-specific and region-specific effects, we normalized Equation 5 by *γ*_*k*,*l*_, the discrete number of edges between the observed cell types within the graph. The normalized value is represented by

(Equation 7) $$\gamma_{k,l}=\sum_{i,j}\delta_{ik}\delta_{jl}\text{,}$$

(Equation 8) $$\bar{A\left(c_{k},c_{l}\right)}=\frac{\sum_{i,j}\alpha_{ij}\delta_{ik}\delta_{jl}}{\gamma_{k,l}}\text{,}$$

which we call the interaction score. This enabled us to transform the network’s localized computations into a global observation, providing insight into the immune response (Figure 1J). Since all attention scores were passed through a softmax layer (Equation 3), we can ensure that the value of a connection is between [0,1]. Thus, Equation 8 can be interpreted as a percentile of how many of the original connections were deemed important. The attention scores are averaged depending on the global label.

It is also critical to differentiate between “NaN” values, which signify unobserved connections, and zeros, which indicate the model has deemed a connection inconsequential with an interaction score of zero. To enhance the robustness of the attention evaluation, we pruned edges between specific cell types if they appeared in less than 1% of all connections for that cell type across all test graphs. This approach does not remove rare cell types but instead filters out infrequent connections that are unlikely to contribute meaningful biological insights. By applying this threshold independently to each cell type, even rare cell types retain their most relevant interactions while minimizing noise introduced by sporadic or biologically insignificant edges.

Note that the model only comprises one GAT layer for this interpretation. The attention scores calculated from Equation 3 are directly correlated to a specific cell type. Introducing additional GAT or GNN layers would have incorporated higher-order neighborhood information in the message passing, complicating the attention score derivation. While additional layers might enhance model performance on the classification task, they would obfuscate the interpretability of the attention mechanism in this context. Our method maintained a direct correlation between attention scores and cells, preserving the interpretability essential for our analysis.

## Resource availability

### Lead contact

Requests for further information and resources should be directed to and will be fulfilled by the lead contact, Manfred Claassen (manfred.claassen@med.uni-tuebingen.de).

### Materials availability

No new materials were created within this study.

### Data and code availability

• The code is publicly available on GitHub: https://github.com/V-Sehra/SHIELD and archived at DOI: https://doi.org/10.5281/zenodo.19471074.^30^
• The HCC dataset is available under DOI: https://doi.org/10.1016/j.cell.2023.07.026 and the diabetes dataset under DOI: https://doi.org/10.1016/j.cmet.2018.11.014.

## Acknowledgments

The authors thank the 10.13039/501100001711Schweizerischer Nationalfonds (SNF; project CRSII5 183478), the 10.13039/501100005846Friedrich-Ebert-Stiftung (FES) for funding, and the International Max Planck Research School for Intelligent Systems (IMPRS-IS) for supporting V.S. B.R. is funded by the 10.13039/501100001659Deutsche Forschungsgemeinschaft (DFG; German Research Foundation) under Germany’s Excellence Strategy - EXC 2180-390900677, the DFG – Clinician Scientist (MINT-CS) program (493665037), the Else Kroner-Fresenius Foundation (EKFS) Clinician Scientist Program, and the 10.13039/501100002347German Federal Ministry of Education and Research (BMBF; grant no. BMBF 01KD2421A). We extend our gratitude to Sebastian Bischoff and Dr. Christoph Hoffmann for not only assisting with conceptual ideas but also proofreading our manuscript.

## Author contributions

V.S. conceptualized the study, developed the methodology, implemented the model, performed formal analysis and validation, created visualizations, developed the software, and wrote all drafts. B.R. curated the data and contributed to methodology development. G.D. contributed to software development. S.B. contributed to methodology development. S.B. and M.C. supervised the research and contributed to the study’s conceptualization. All authors reviewed and edited the manuscript.

## Declaration of interests

M.C. is co-founder of the company Vicinity Bio GmbH, a company offering multiplexed tissue imaging as a service.

## Declaration of generative AI and AI-assisted technologies in the writing process

During the preparation of this work, the author V.S. used ChatGPT to optimize wording and correct spelling. After using this tool, the author reviewed and edited the content as needed and takes full responsibility for the content of the publication.

## Supplemental information

Document S1. Figures S1–S39, Tables S1–S5, supplemental methods, and supplemental notes

Document S2. Article plus supplemental information
