# Supplementary material for: SHIELD: A weakly supervised graph attention neural network for decoding disease-relevant cell-cell interactions
Source: Patterns (N Y). 2026 May 22;7(7):101562. doi: 10.1016/j.patter.2026.101562 (PMC13366524; doi:10.1016/j.patter.2026.101562)
Supplement: Document S1. Figures S1–S39, Tables S1–S5, supplemental methods, and supplemental notes [file mmc1.pdf]

**Patterns, Volume *7***

## Supplemental information

# SHIELD: A weakly supervised graph attention neural network for decoding disease-relevant cell-cell interactions

**Vivek Sehra, Benjamin Ruf, Gabriel Duval, Sepideh Babaei, and Manfred Claassen**

# 1 Supplementary Methods

**Uniform-Attention GNN Baseline:**
We trained a single-layer GAT in where all edge attention weights were fixed to 1, i.e. a GNN, effectively removing the learning signal from the edges while keeping the same node features and topology. This isolates the predictive contribution of the trainable attention mechanism.

## 1.1 Robustness experiments

To investigate the robostness of SHIELDS interactions Scores (IS) we introduced multiple noising shemes and reevaluated the performance and IS of SHIELD.

## 1.2 Randomized Edges

To fully interrogate the role of spatial structure, we designed a controlled edge-randomization strategy with increasing levels of spatial ablation, applied globally across the tissue. The following text describes this ablation experiment and has been added to the Supplementary Methods section:

**Spatial ablation experiment**:
We constructed three benchmark graph variants per patient, representing increasing removal of spatial information: (1) SHIELD's default setting (**Radial graph**) using spatially constrained radial neighborhoods (e.g., r = 200$\mu$m), preserves spatial structure. (2) Same degree shuffled (**sameCon**), where each node retains its degree (i.e., average number of connections), but connections are randomly reassigned across the entire tissue, disrupts spatial proximity while preserving global graph topology. (3) 10% of all possible connections (**percent**) constructs sparse graphs by randomly selecting 10% of all possible $n \times n$ cell pairs.

**Population ablation experiment**:
To empirically evaluate SHIELD's robustness under strong cell-type frequency imbalances, we performed experiments in which both PD-L1$^{+}$ and PD-L1$^{-}$ M2 macrophage populations were artificially downsampled within each patient's tissue to 70 % and 30 % of their original counts (Fig. S.7,S.9,S.8). This per-patient downsampling design prevents the introduction of global, patient-level biases. To further determine the point at which SHIELD no longer detects the MAIT–macrophage connection (i.e., when the interaction signal breaks down), we additionally downsampled the macrophage populations to 16 %, 8 %, and 2 % of their original frequencies (Fig. S.6).

**Noising the labels experiment**:
To evaluate the effects of inconsistent ROI labeling and other sources of boundary-related noise, we artificially augmented the region labels. Specifically, we systematically perturbed the ROI labels to simulate both realistic and extreme annotation errors. All noise was introduced at the *single-cell* level, based on the tissue-type composition within a 500,$\mu$m radial neighborhood. The resulting changes in tissue composition under different noise conditions.

1. Probability noise— A cell's new label was assigned probabilistically according to the proportion of tissue types in its neighborhood. For example, if a cell had 20 Healthy and 10 Rim neighbors, it was relabeled as Healthy with probability $2/3$ and Rim with $1/3$. This mimics the variability expected if multiple experts independently annotated the same region and a consensus label was taken.

2. Dominance noise (Healthy-, Rim-, Core-dominant) — If at least one neighbor belonged to the dominant tissue type, the cell's label was forcibly changed to that type, regardless of the rest of the neighborhood composition. This simulates more extreme and systematic misclassification, such as consistently overextending one region's boundary. Based on domain expertise, the Healthy- and Rim-dominant schemes are more biologically plausible (due to gradual tissue transitions), whereas Core-dominant mislabeling is largely unrealistic, as Core regions typically have sharp histological boundaries.

# 2 Supplementary Notes

**Uniform-Attention GNN Baseline:**
The one layer GNN shows a notable drop in accuracy (Fig. S.2f)-h))($\sim 20\%$ in Normal Liver and Core) compared to the full SHIELD model. Nevertheless, performance remains above random guessing, indicating that node features alone retain some discriminative power. The gap between the fixed-attention and trainable-attention GNNs thus reflects

the added value of edge-level learning for generalization.

**Spatial ablation experiment**:
We evaluated the performance of the classifier for these alternative input graphs for the HCC dataset and found that the radial graph model, i.e. the graph with spatial proximity information performed best with its default input graphs, with average (balanced) test accuracies of around 82% (Fig.S.2 a)-c)). With **random** input graphs, the accuracy decreased notably, in particular for biologically more diffuse and difficult to distinguish classes like the Rim region in HCC. Both the percent and sameCon input graphs showed slight performance decrease. We conclude that while node-level features are strong predictors default input graphs encoding spatial structure of cell arrangements improves class separability and biological coherence in the learned representations.

**Population ablation experiment**:
Across both perturbation levels, SHIELD continued to identify MAIT–M2 macrophage interactions as significantly enriched in the Core and Rim regions, as confirmed by Mann–Whitney U tests with FDR correction (Figs. S.8, S.9 and S.7). Interestingly, reducing the MAC population led to a small shift in classification behavior: the models classified the Healthy region slightly better, but the Rim region slightly worse. Importantly, the overall classification accuracy remained stable within $\pm$1–2% of the original performance, indicating that SHIELD's key biological findings are robust to substantial perturbations in cell type abundance.
The extreme population reduction experiments further illustrate the sensitivity limits of SHIELD. When the macrophage (MAC) population was reduced to 2 % and 8 % of its original frequency, the Tumor region no longer exhibited detectable $MAC^-$ interactions, indicating that below this threshold the signal is lost. In contrast, across all three conditions, the Healthy region consistently identified MAIT cells and both $MAC^+$/$MAC^-$ populations as important interacting partners, with the interaction even becoming more pronounced. This strengthening likely reflects a distribution shift that increases the emphasis on distinguishing between Tumor Core and Healthy Liver (Table S5). However, at 2 % relative (0.2 % absolute) MAC frequency, the model could no longer reliably determine the interaction's importance within the Rim region, Fig. S.6.

**Noising the labels experiment**:
The classification accuracies under these noise scenarios are reported in Fig. S.2 i–k. As expected, Core-dominant noise caused the largest drop in performance, while Probability and Rim-dominant noise had only minimal impact, reflecting SHIELD's resilience to realistic annotation uncertainty. All models showed a shift in classification bias towards the dominant tissue type, consistent with the altered ROI definitions.

Critically, we examined the stability of SHIELD's top 8 interaction scores under these noisy conditions (Figs. S.12 S.11 S.10). Even under severe label corruption, the majority of high-importance interactions from the original model were preserved:
For Probability and Rim-dominant noise, $\sim$ 82% original top interactions were recovered across ROIs. Even under the unrealistic Core-dominant scheme,$\sim$ 73% of the original top interactions remained, with MAIT–$MAC^+$ still retained in Rim. This robustness is consistent with our earlier nearest-neighbor (NN) vs. attention-score analysis (Fig. 3, manuscript), where we showed that SHIELD prioritizes rare but biologically validated interactions — such as MAIT–$MAC^+$ crosstalk — even when these are underrepresented in the raw neighborhood structure. Such stability under noise suggests that SHIELD captures biologically meaningful patterns rather than overfitting to precise ROI boundaries or specific phenotype calls.

## 2.1 Compute Time

The runtime for graph construction, model training, and evaluation is shown below for the two main datasets. Times are reported as wall-clock hours:minutes:seconds on our computational setup (NVIDIA GeForce GTX 1080 Ti)

Table. S1: **Wall-clock times for the main computational stages in SHIELD.**

| Dataset | Graph Creation | Training | Evaluation |
|---|---|---|---|
| HCC | 1:33:00 | 02:09:04 | 00:31:34 |
| CRC | 00:58:07 | 03:43:57 | 00:22:16 |
| Diabetes | 01:10:07 | 02:40:45 | 00:35:08 |

# 3 Additional Supplementary Tables

Table. S2: **Performance of SHIELD for the train and test Patients individually for the hcc data set.**

| **Patient ID** | **Total accuracy ± STD** [%] | **Healthy Liver** [%] | **Tumor Core** [%] | **Rim** [%] |
|---|---|---|---|---|
| | | **Test Patients** | | |
| LHCC45 | 95.02 ± 21.75 | 98.92 ± 10.36 | 86.51 ± 34.17 | 99.64 ± 6.00 |
| LHCC51 | 36.14 ± 48.04 | 12.89 ± 33.51 | 5.06 ± 21.92 | 90.48 ± 29.35 |
| LHCC53 | 100.00 ± 0.00 | 100.00 ± 0.00 | 100.00 ± 0.00 | 100.00 ± 0.00 |
| Pat52 | 98.92 ± 10.36 | 100.00 ± 0.00 | 100.00 ± 0.00 | 96.75 ± 17.74 |
| Pat53 | 99.16 ± 9.14 | 100.00 ± 0.00 | 97.47 ± 15.70 | 100.00 ± 0.00 |
| | | **Train Patients** | | |
| LHCC35 | 98.67 ± 11.44 | 96.87 ± 17.42 | 99.28 ± 8.47 | 99.88 ± 3.47 |
| LHCC36 | 99.76 ± 4.90 | 99.28 ± 8.47 | 100.00 ± 0.00 | 100.00 ± 0.00 |
| LHCC44 | 99.72 ± 5.29 | 99.28 ± 8.47 | 99.88 ± 3.47 | 100.00 ± 0.00 |
| LHCC46 | 99.80 ± 4.48 | 99.40 ± 7.74 | 100.00 ± 0.00 | 100.00 ± 0.00 |
| LHCC47 | 100.00 ± 0.00 | 100.00 ± 0.00 | 100.00 ± 0.00 | 100.00 ± 0.00 |
| LHCC48 | 99.88 ± 3.47 | 99.64 ± 6.00 | 100.00 ± 0.00 | 100.00 ± 0.00 |
| LHCC49 | 67.03 ± 47.01 | 96.02 ± 19.54 | 100.00 ± 0.00 | 5.06 ± 21.92 |
| LHCC52 | 99.64 ± 6.00 | 98.92 ± 10.36 | 100.00 ± 0.00 | 100.00 ± 0.00 |
| LHCC54 | 65.74 ± 47.46 | 97.59 ± 15.33 | 0.00 ± 0.00 | 99.64 ± 6.00 |
| LHCC68 | 96.59 ± 18.16 | 90.12 ± 29.84 | 100.00 ± 0.00 | 99.64 ± 6.00 |

Table. S3: **Performance of SHIELD for the train and test Patients individually for the diabetes data set.**

| | **Test Patients** | | |
|---|---|---|---|
| **Patient ID** | **Spleen section** | **Stage** | **Total accuracy ± STD** |
| 6380 | Tail | Onset | 32.0 % ± 24.0 % |
| 6380 | Body | Onset | 32.0 % ± 24.0 % |
| 6380 | Head | Onset | 32.0 % ± 24.0 % |
| 6264 | Tail | Long-duration | 88.0 % ± 12.0 % |
| 6264 | Body | Long-duration | 88.0 % ± 12.0 % |
| 6264 | Head | Long-duration | 88.0 % ± 12.0 % |
| 6278 | Tail | Non-diabetic | 90.0 % ± 7.0 % |
| 6278 | Body | Non-diabetic | 90.0 % ± 7.0 % |
| 6278 | Head | Non-diabetic | 90.0 % ± 7.0 % |
| | **Train Patients** | | |
| **Patient ID** | **Spleen section** | **Stage** | **Total accuracy ± STD** |
| 6362 | Tail | Onset | 99.0 % ± 1.0 % |
| 6362 | Body | Onset | 99.0 % ± 1.0 % |
| 6418 | Tail | Long-duration | 100.0 % ± 0.0 % |
| 6418 | Body | Long-duration | 100.0 % ± 0.0 % |
| 6126 | Tail | Non-diabetic | 100.0 % ± 0.0 % |
| 6126 | Body | Non-diabetic | 100.0 % ± 0.0 % |
| 6414 | Tail | Onset | 98.0 % ± 0.0 % |
| 6414 | Body | Onset | 98.0 % ± 0.0 % |
| 6180 | Tail | Long-duration | 98.0 % ± 2.0 % |
| 6180 | Body | Long-duration | 98.0 % ± 2.0 % |
| 6134 | Tail | Non-diabetic | 100.0 % ± 0.0 % |
| 6134 | Body | Non-diabetic | 100.0 % ± 0.0 % |
| 6228 | Tail | Onset | 100.0 % ± 0.0 % |
| 6228 | Body | Onset | 100.0 % ± 0.0 % |
| 6089 | Tail | Long-duration | 100.0 % ± 0.0 % |
| 6089 | Body | Long-duration | 100.0 % ± 0.0 % |
| 6386 | Tail | Non-diabetic | 100.0 % ± 0.0 % |
| 6386 | Body | Non-diabetic | 100.0 % ± 0.0 % |

Table. S4: **The baseline Performance of three significantly larger models in comparison to SHIELD.**

| Model description | Train accuracy ± STD [%] | Test accuracy ± STD[%] |
|---|---|---|
| **HCC** | | |
| Linear | 33 ±0 | 33±0 |
| MLP | 33 ±0 | 33±0 |
| Three Layer GNN | 70 ±1 | 61±1 |
| Three Layer GAT | 71 ±6 | 57±2 |
| $S^3$-CIMA | 75 ±7 | 67 |
| **SHIELD** | 83±2 | 68±3 |
| **T1D** | | |
| Linear | 33 ±0 | 33±0 |
| MLP | 33 ±0 | 33±0 |
| Three Layer GNN | 99 ±1 | 70±2 |
| Three Layer GAT | 99 ±1 | 76±4 |
| $S^3$-CIMA | 95 ±3 | 75±15 |
| **SHIELD** | 99±0 | 70±9 |

Table. S5: **Reduction of the macrophage population to test when SHIELD no longer detects MAIT–macrophage interactions as significant.** For each patient, macrophages were reduced to the indicated percentile independent of tissue origin. The total number of immune cells is 1,416,440.

| **Relative reduced frequency** | **absolute count** | **absolute new frequency** | **Healthy** | **Rim** | **Core** |
|---|---|---|---|---|---|
| Original | 134,813 | 9.52% | 90,874 | 34,545 | 9,394 |
| 70% | 94,369 | 6.85% | 63,765 | 24,057 | 6,547 |
| 30% | 40,443 | 3.05% | 27,437 | 10,244 | 2,762 |
| 16% | 21,573 | 1.66% | 14,641 | 5,466 | 1,466 |
| 8% | 10,785 | 0.83% | 7,293 | 2,774 | 718 |
| 2% | 2,697 | 0.2% | 1,797 | 698 | 202 |

## 4 Additional Supplementary Figures

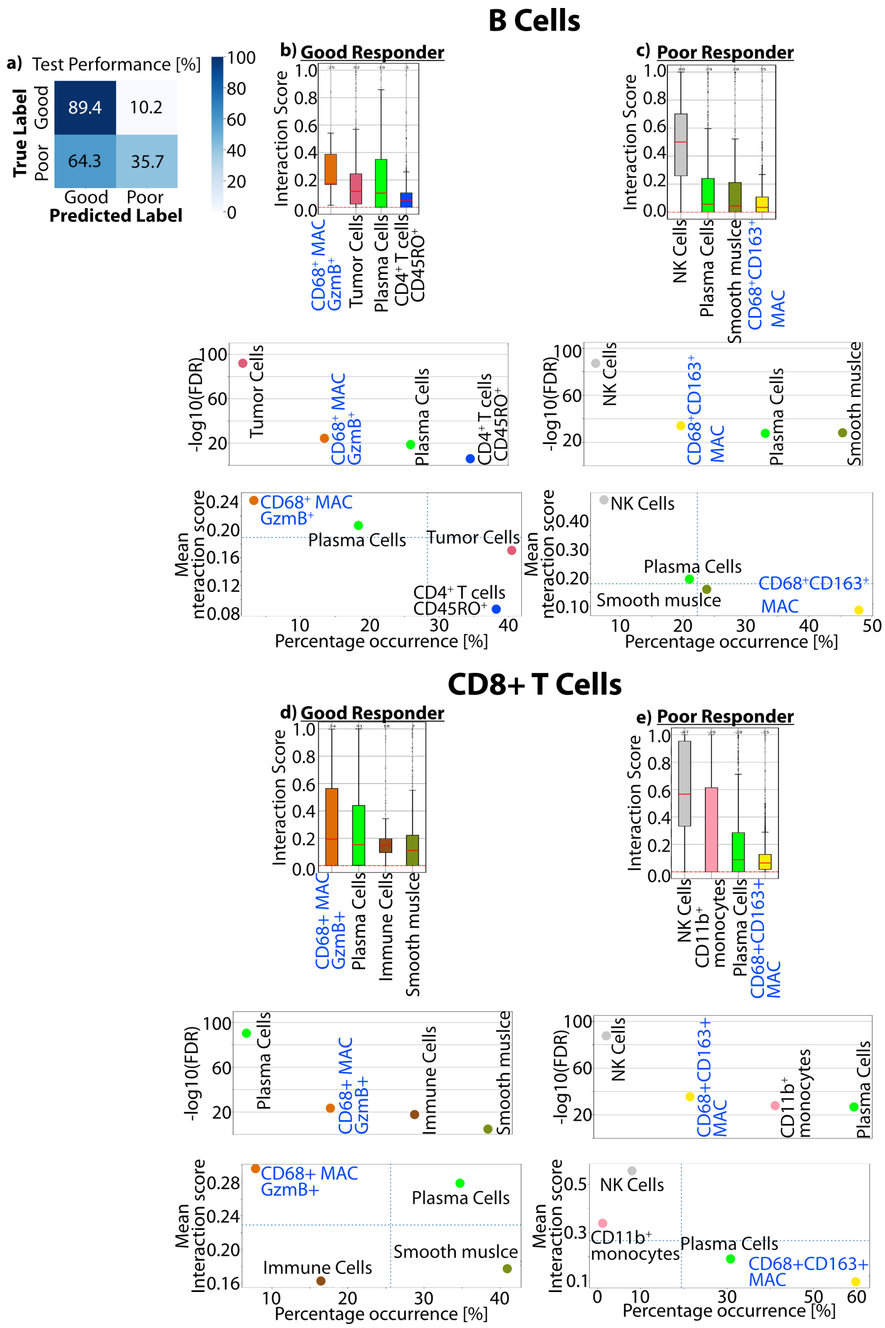


Figure S.1: **Classification performance and cell-cell interaction analysis for colorectal cancer (CRC). a)** Confusion matrix showing SHIELD's test performance using Voronoi-based compartment sampling to classify treatment response (Good vs. Poor responders). Balanced accuracy was 64.3%.

The follwoing panels show the *Interaction Scores* between the source cell type and their four highest partners, sorted by median. Red dashed lines indicate the overall median *Interaction Score* across all cell types. Green bands denote the interquartile range (25th to 75th percentile), representing the background distribution. Above each boxplot, the $-\log_{10}$(FDR)-corrected Mann–Whitney U test value quantifies statistical significance. Below, nearest-neighbor (NN) control plots display the relationship between mean interaction score (y-axis) and spatial co-occurrence (x-axis, average NN percentile), highlighting rare but biologically meaningful interactions that SHIELD prioritizes.

**b–c)** Interaction analysis with B cells. SHIELD highlights rare but significant interactions between B cells and CD68$^+$CD163$^+$ macrophages (MAC), enriched in Poor responders. This macrophage–B cell interaction was previously reported in the original publication and is successfully recovered by SHIELD, despite its low nearest-neighbor co-occurrence. **d–e)** Interaction analysis with CD8$^+$ T cells. SHIELD identifies strong and stage-specific interactions with CD68$^+$GzmB$^+$ macrophages, particularly in Poor responders. These interactions suggest suppressive crosstalk between cytotoxic T cells and macrophages and match prior biological findings in CRC.

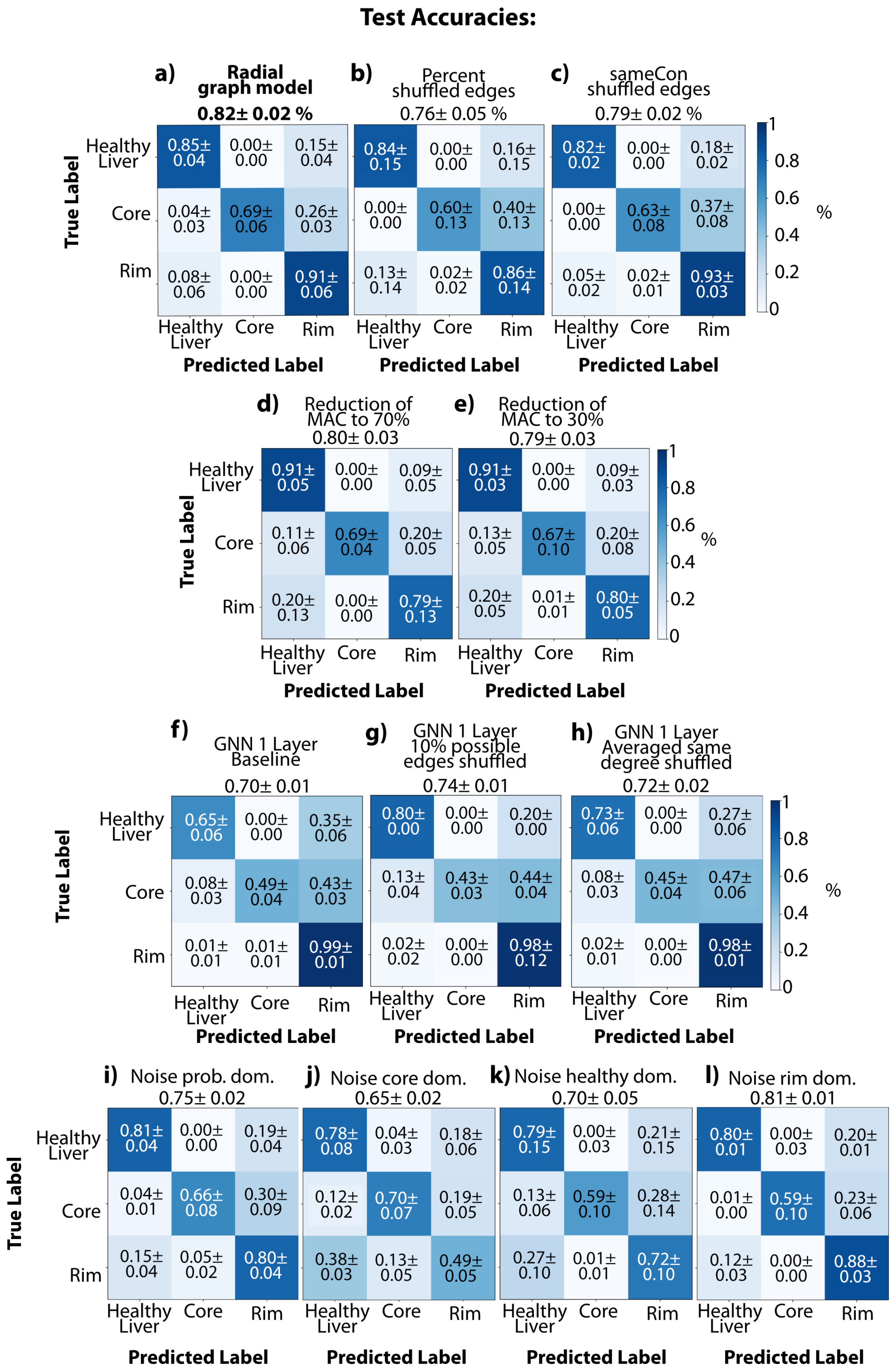


Figure S.2: **All performances for the noising of labels and edge shuffling.** Panels: (a) Original radial graph model on the bucket sampling; (b) shuffled edges with each node connected to 10% of all possible nodes (dense random graph, **percent**); (c) shuffled edges with original degree preserved (**sameCon**); (d) PD-L1$^+$ and PD-L1$^+$ macrophages population reduced to 70% of the original; (e) PD-L1$^+$ and PD-L1$^+$ macrophages population reduced to 30% of the original; (f) one-layer GNN baseline; (g) one-layer GNN baseline with shuffled edges **percent**; (h) one-layer GNN baseline with shuffled edges **sameCon**; (i) probabilistic noisy labels based on neighbourhood composition (e.g., 20 healthy and 10 tumor neighbours $\Rightarrow \frac{2}{3}$ healthy, $\frac{1}{3}$ tumor); (j) core-dominant noisy labels (if any tumor neighbour exists, set label to core); (k) healthy-dominant noisy labels; (l) rim-dominant noisy labels.

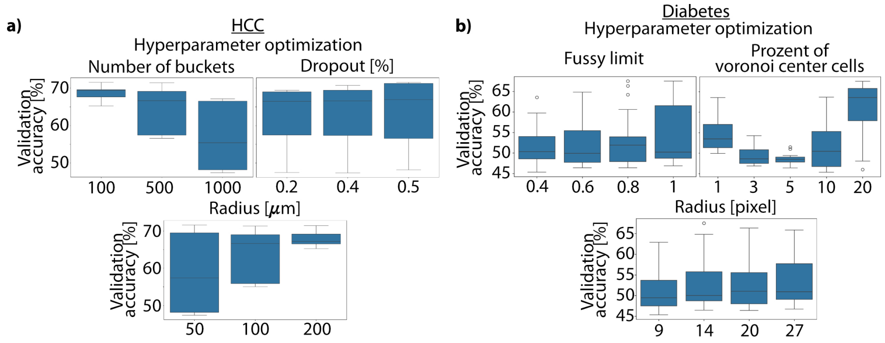


Figure S.3: **Hyperparameter search results for the validation splits in the HCC and diabetes datasets.** The training set was split into three validation datasets, and each configuration was trained five times, with performance averaged across runs. Boxplots display the distribution of validation accuracy for all tested configurations. **a)** HCC hyperparameter optimization results, showing validation accuracy across variations in radius, dropout rate, number of buckets, and fuzzy limit. **b)** Diabetes hyperparameter optimization results, reporting validation accuracy across the percentage of voronoi center cells, radius, and fuzzy limit.

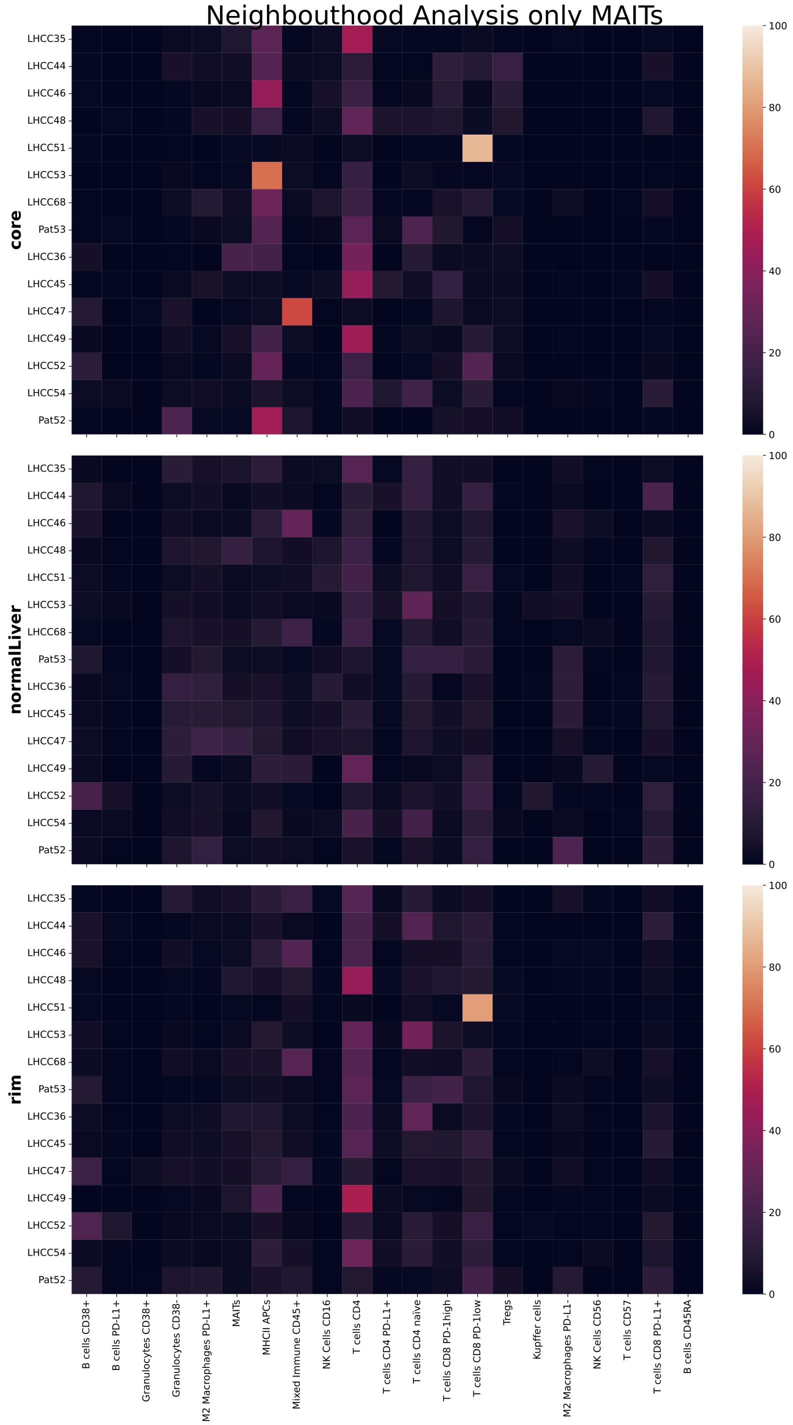


Figure S.4: **Nearest-neighbor analysis of MAIT cells across ROIs.** Heatmaps show the composition of nearest neighbors for MAIT cells across all patients, stratified by region of interest (Core, Normal Liver, and Rim). Each row corresponds to an individual patient, and each column represents a neighboring cell type. Color intensity reflects the frequency of each cell type appearing as a spatial neighbor to MAIT cells, scaled from 0 to 100%. This analysis illustrates the diversity and distribution of local immune context for MAITs across different tissue compartments.

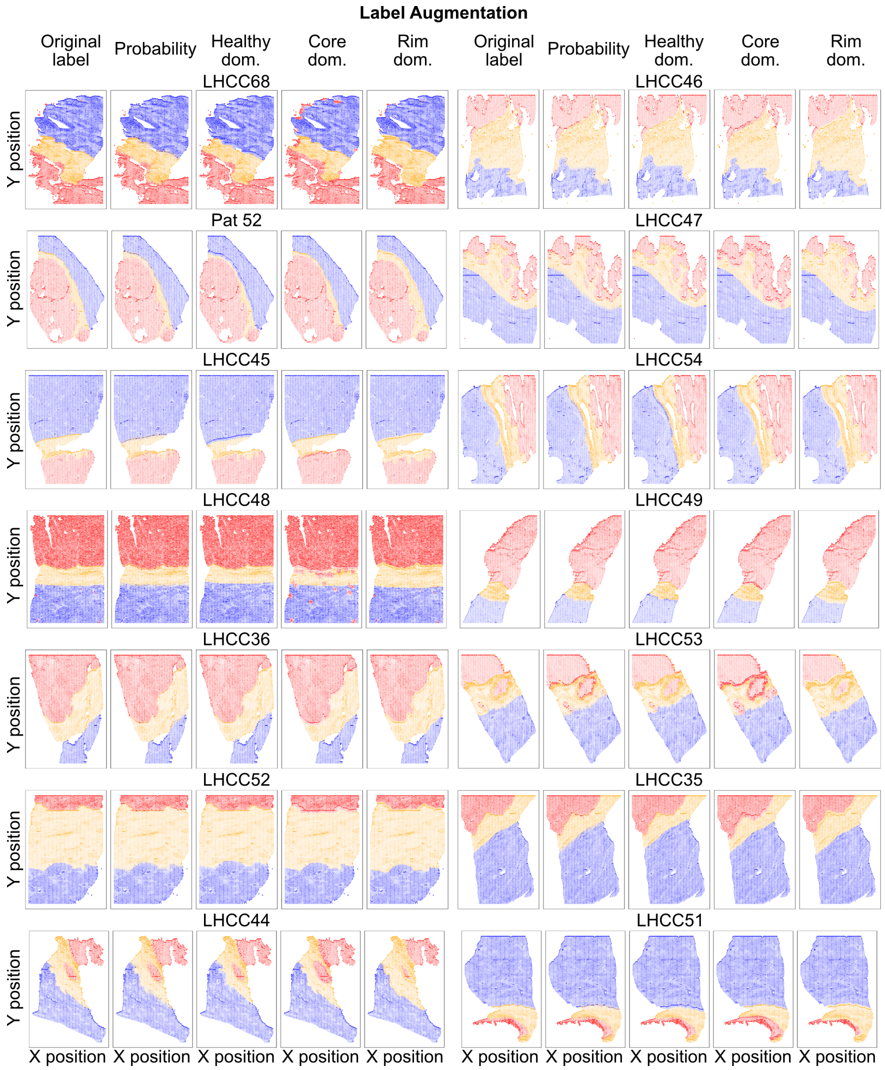


Figure S.5: **Label noise augmentation strategies in the HCC dataset.** Visualization of region-of-interest (ROI) label perturbations for selected HCC samples. Each row corresponds to one patient tissue section. The first column shows the original expert-annotated ROI labels (Healthy in blue, Rim in yellow, Core in red). The second column displays probabilistic label augmentation based on local neighborhood composition (e.g., labels re-assigned by majority vote within a 500 µm radius). The remaining columns illustrate three deterministic label noise schemes: **Healthy-dominant**, **Core-dominant**, and **Rim-dominant**—where any neighboring presence of a respective class results in its propagation into surrounding cells. These augmentations simulate realistic and extreme annotation errors, enabling assessment of SHIELD's robustness to noisy or ambiguous spatial boundaries.

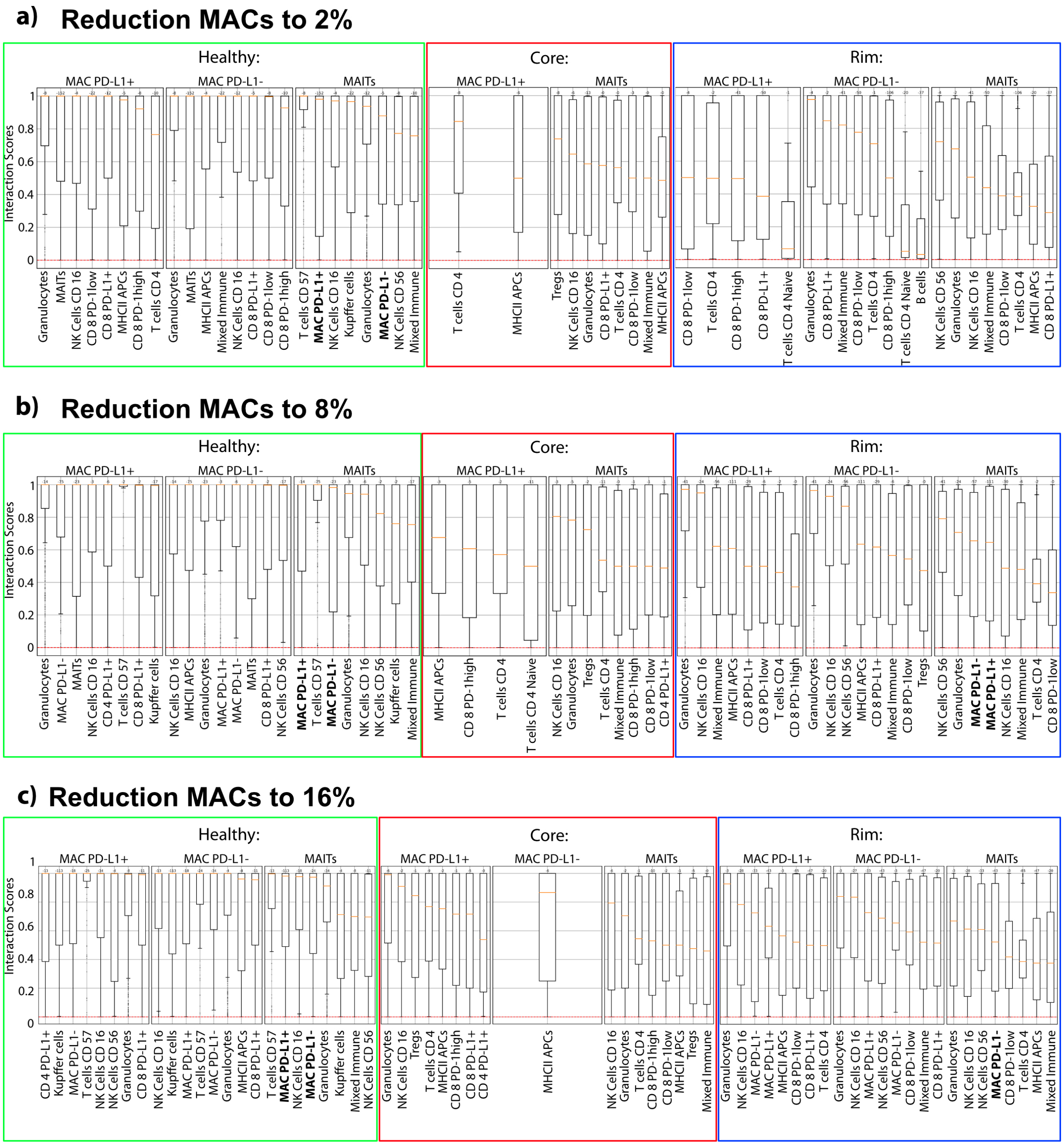


Figure S.6: **Reduction of the macrophage (MAC) population to evaluate the performance limits of SHIELD and identify the threshold at which Interaction Scores lose reliability.** For each reduction level, Interaction Scores are shown for MAC⁺ and MAC⁻ MAIT cells per ROI. Missing entries indicate that no Interaction Scores were detected under the respective condition. When fewer than eight interactions are displayed, this reflects the total number of significant interactions identified. The original findings are highlighted in bold text. Additional details are provided in Table S5. Colored boxes serve solely as visual aids to distinguish between different ROIs and their corresponding Interaction Scores at each reduction level. (**a**) Relative frequency reduced to 2% (absolute 0.2%). (**b**) Relative frequency reduced to 8% (absolute 0.83%). (**c**) Relative frequency reduced to 16% (absolute 1.66%).

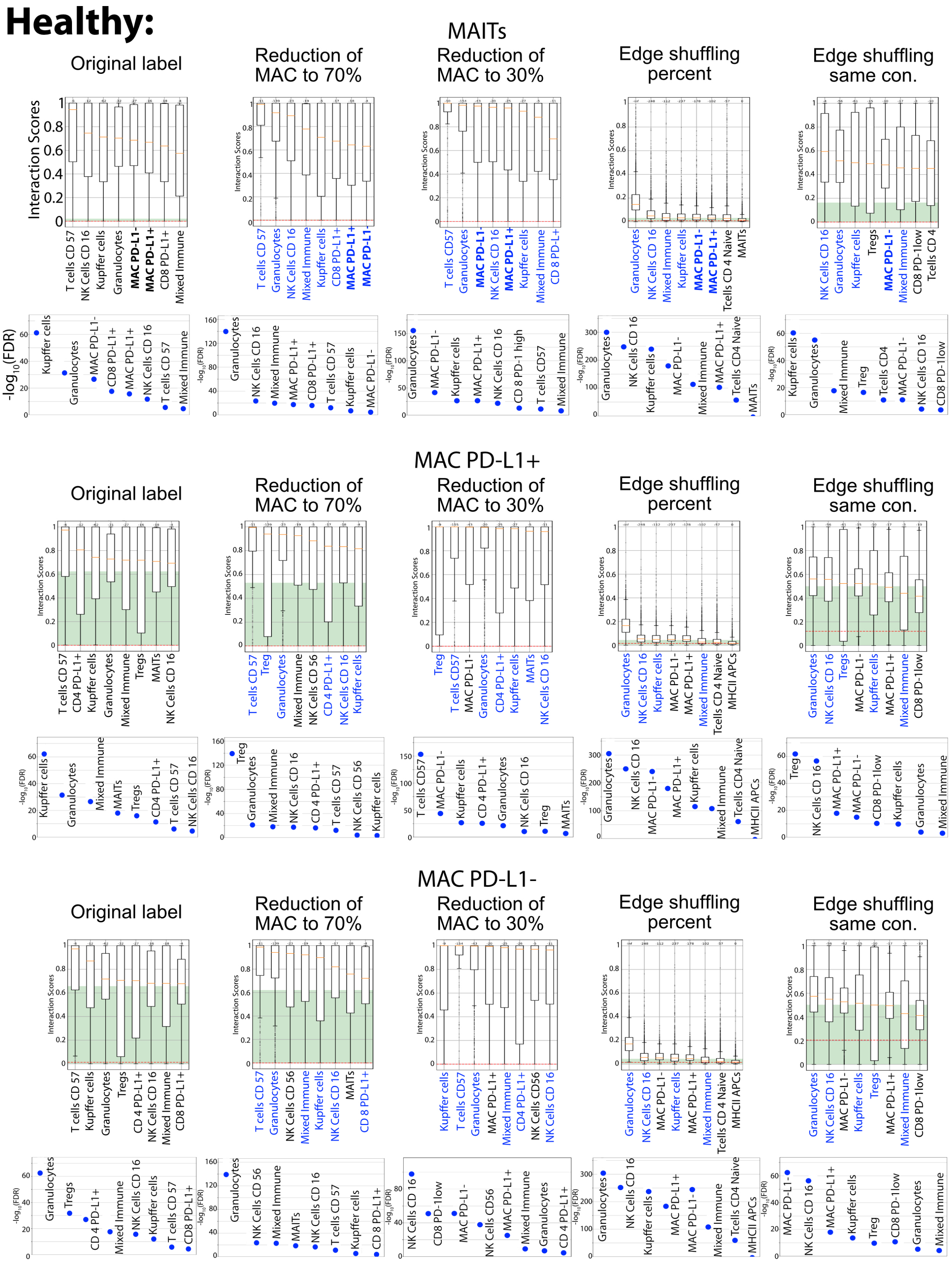


Figure S.7: **Healthy region: The top eight interactions from the original findings and the significant finding in bold, reduced-MAC to 70% and 30% of the original population and the two different edge shuffling schemes.** The overlap with the original overlaps in blue.

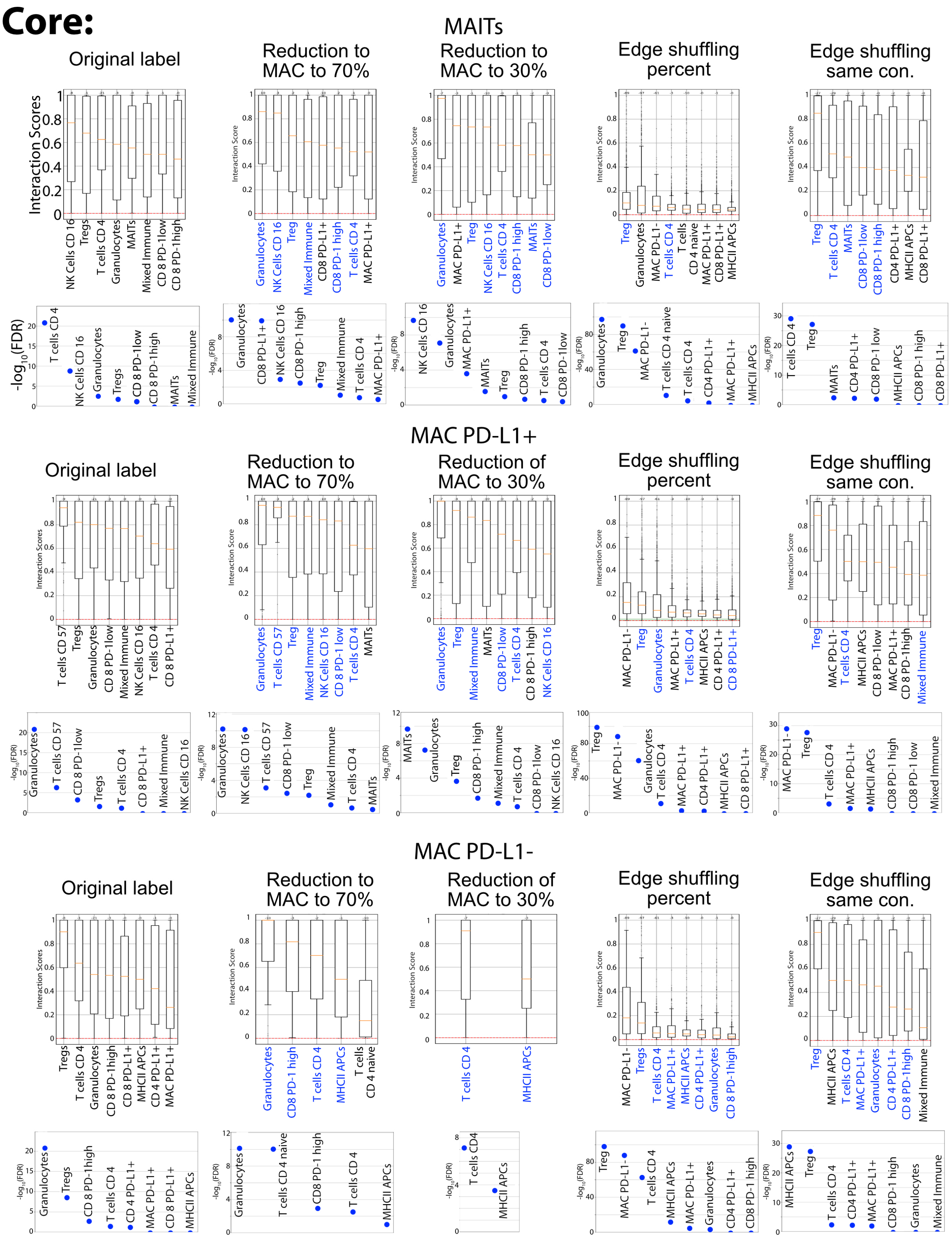


Figure S.8: **Core region: The top eight interactions from the original findings and the significant finding in bold, reduced-MAC to 70% and 30% of the original population and the two different edge shuffling schemes.** The overlap with the original overlaps in blue.

# Rim:

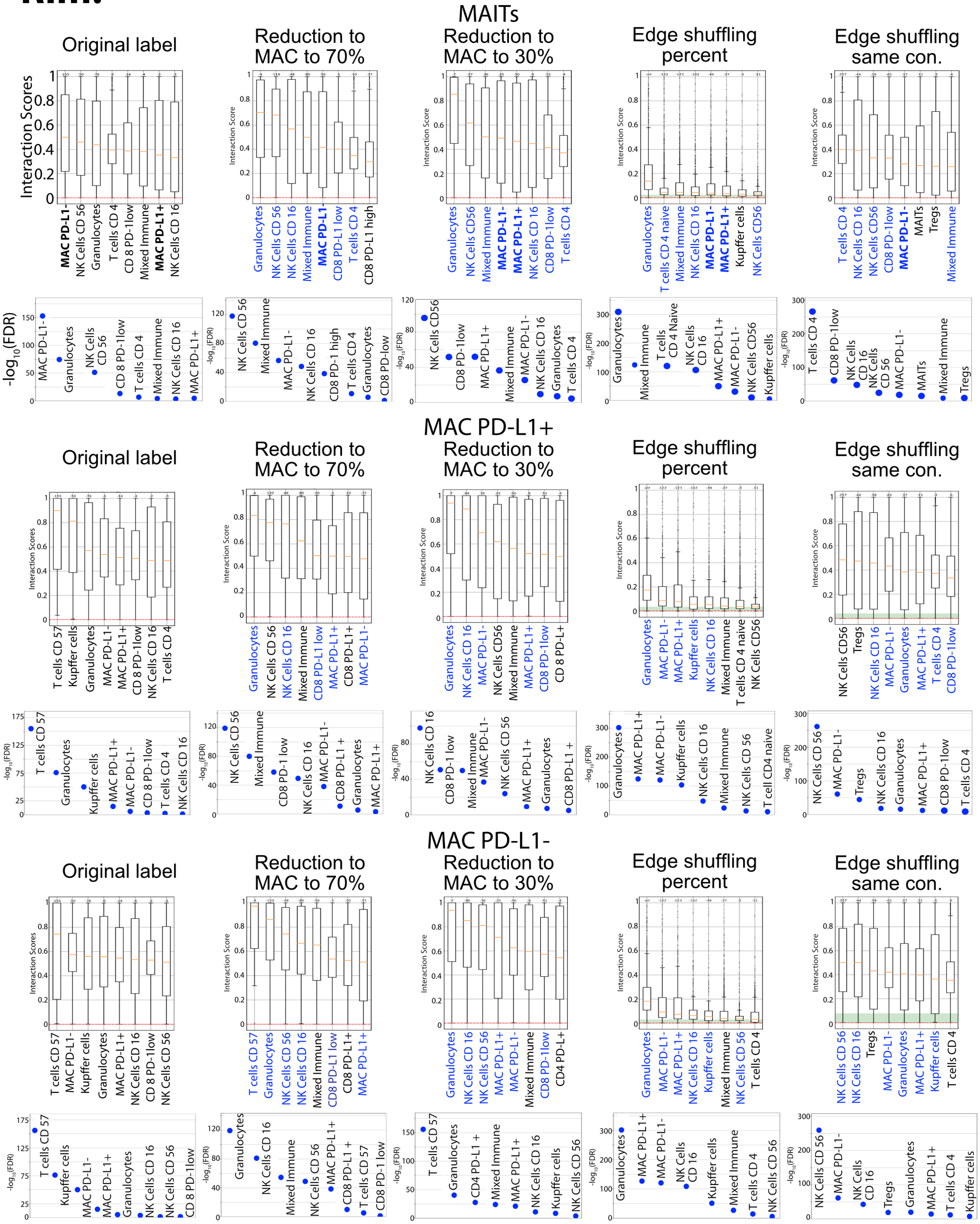

Figure S.9: **Rim region: The top eight interactions from the original findings and the significant finding in bold, reduced-MAC to 70% and 30% of the original population and the two different edge shuffling schemes.** The overlap with the original overlaps in blue.

# Healthy:

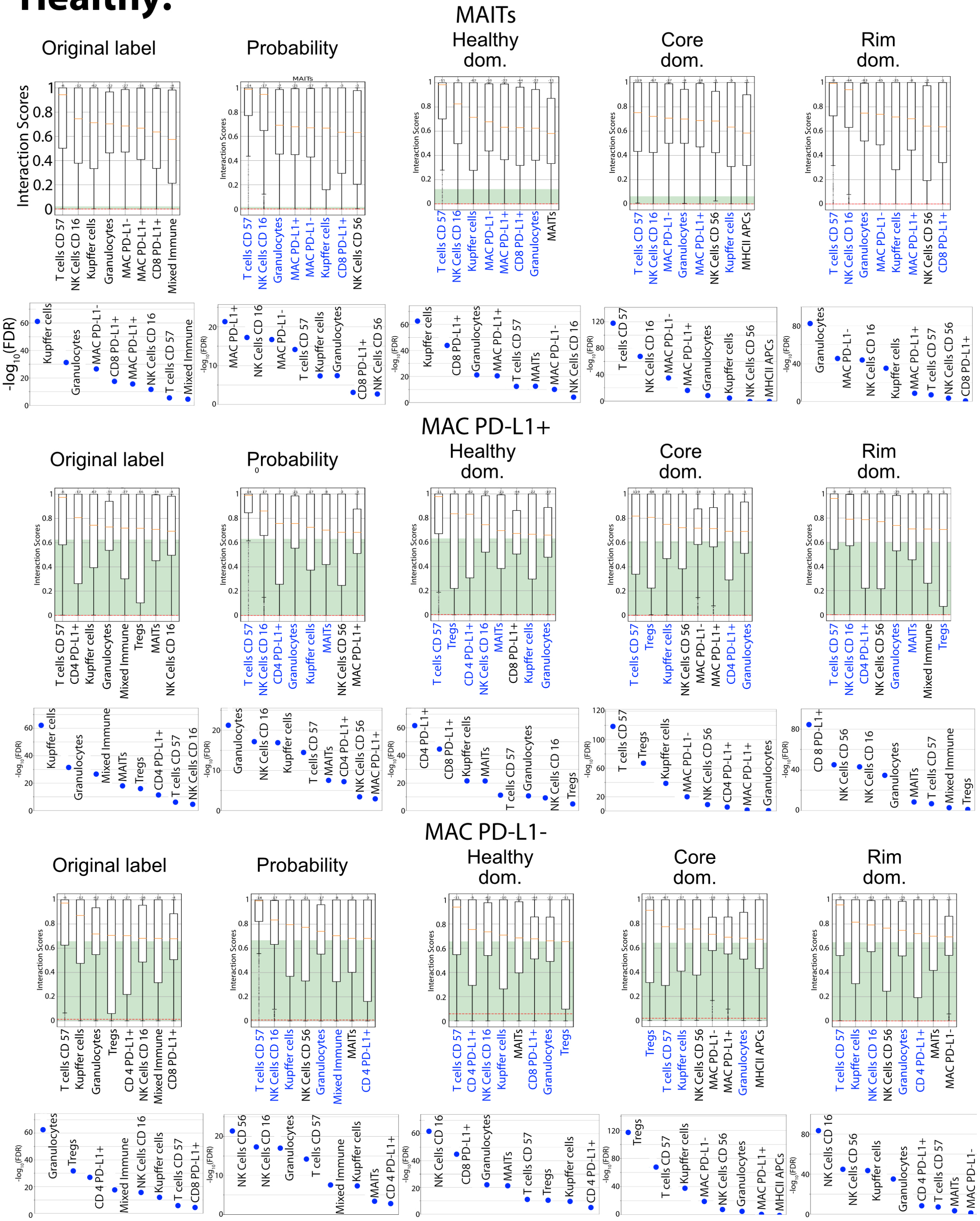


Figure S.10: **Impact of label noise on SHIELD's top 8 interaction scores across the healthy tissue:** For each region, the left column indicate the original top 8 interaction Scores and their corresponding FDR values, while blue highlights the overlap between noisy and original results. Noise schemes include: (i) Probability — label reassignment based on neighborhood composition; (ii) Healthy-dominant, Core-dominant, and Rim-dominant — where presence of a given tissue type within the neighborhood forces label change to that type. The overlap remains high for biologically plausible noise schemes (Probability, Rim-dominant), but drops sharply for the unbiological Core-dominant case.

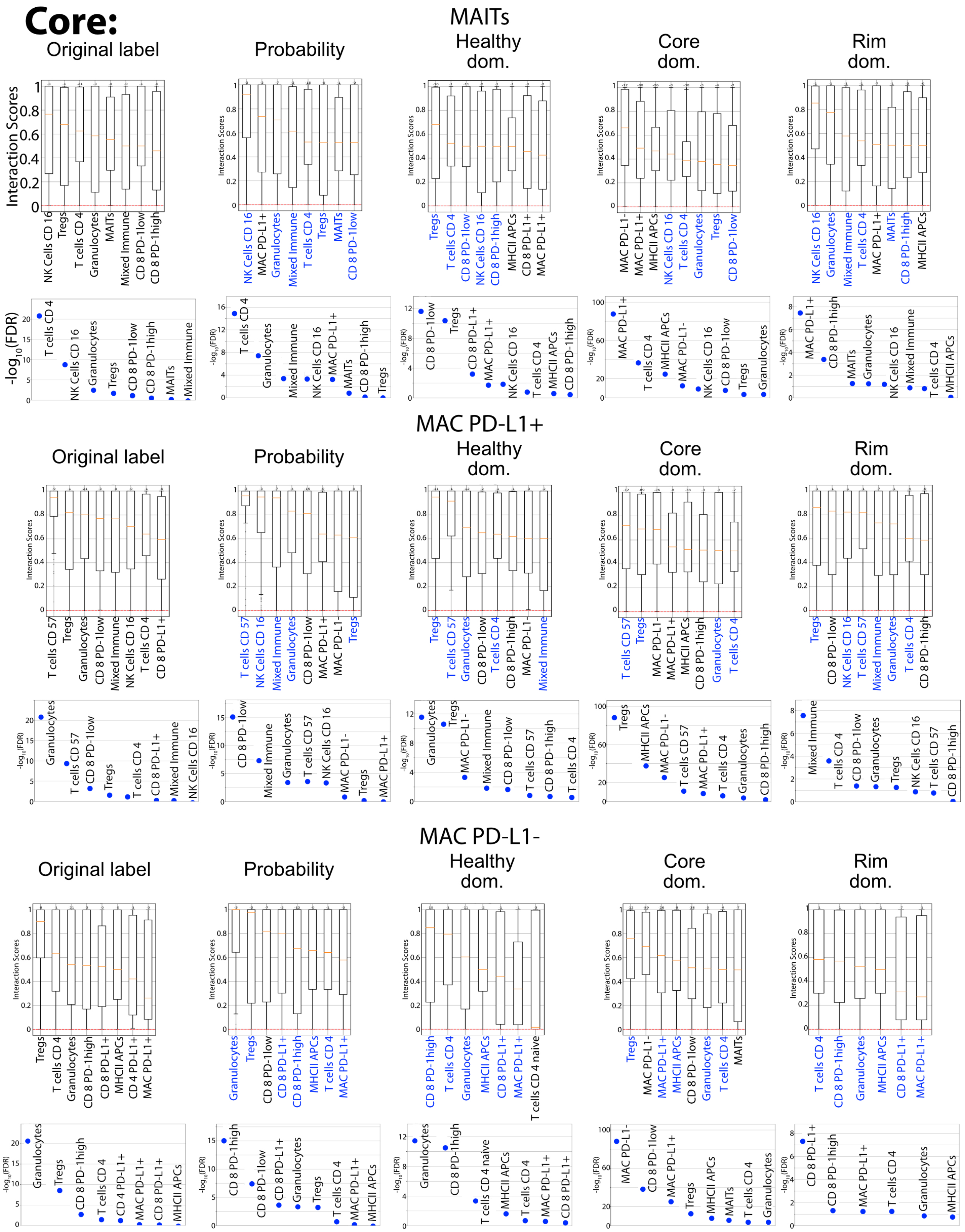


Figure S.11: **Impact of label noise on SHIELD's top 8 interaction scores across the tumor core:** For each region, the left column indicate the original top 8 interaction Scores and their corresponding FDR values, while blue highlights the overlap between noisy and original results. Noise schemes include: (i) Probability — label reassignment based on neighborhood composition; (ii) Healthy-dominant, Core-dominant, and Rim-dominant — where presence of a given tissue type within the neighborhood forces label change to that type. The overlap remains high for biologically plausible noise schemes (Probability, Rim-dominant), but drops sharply for the unbiological Core-dominant case.

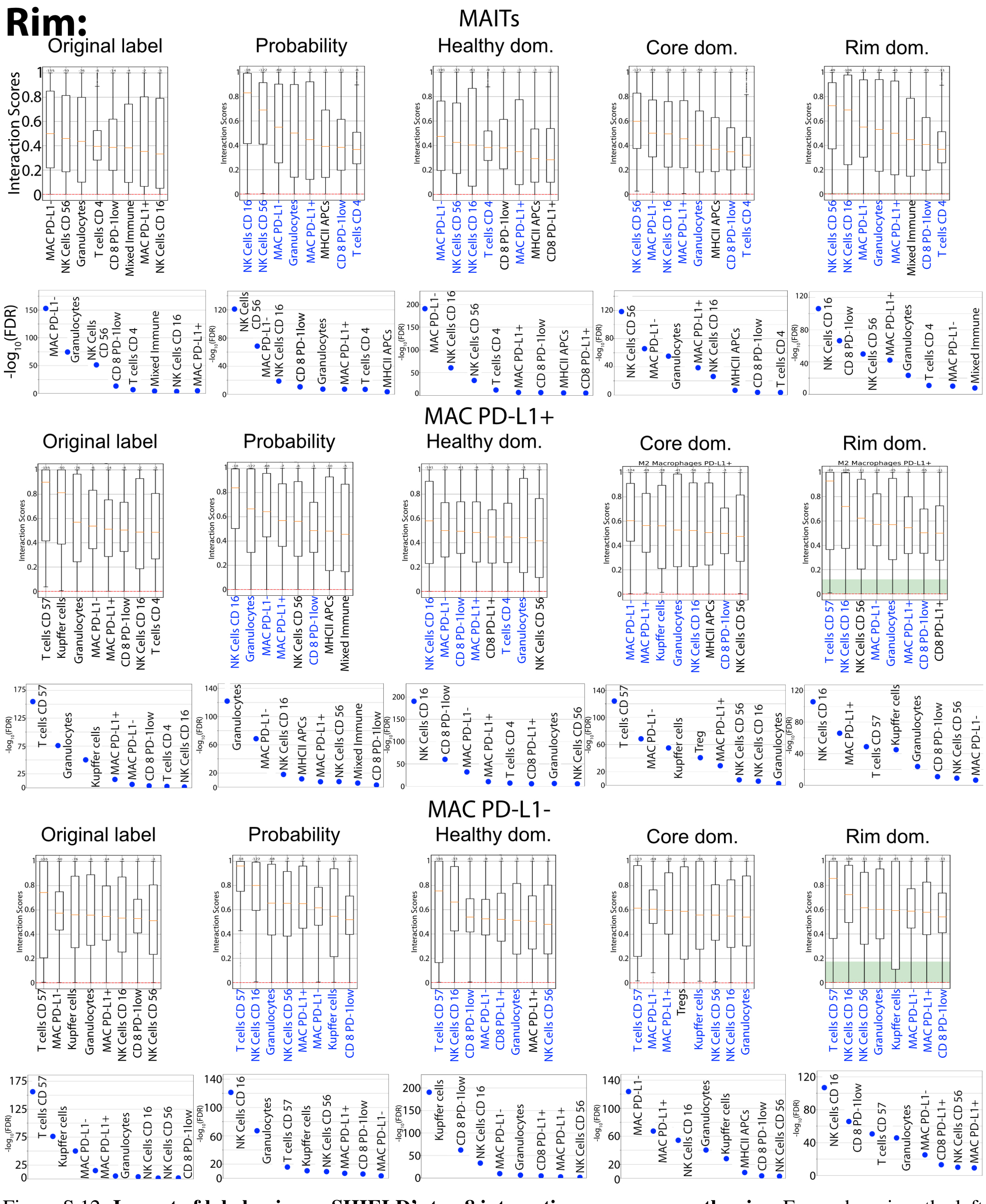


Figure S.12: **Impact of label noise on SHIELD's top 8 interaction scores across the rim:** For each region, the left column indicate the original top 8 interaction Scores and their corresponding FDR values, while blue highlights the overlap between noisy and original results. Noise schemes include: (i) Probability — label reassignment based on neighborhood composition; (ii) Healthy-dominant, Core-dominant, and Rim-dominant — where presence of a given tissue type within the neighborhood forces label change to that type. The overlap remains high for biologically plausible noise schemes (Probability, Rim-dominant), but drops sharply for the unbiological Core-dominant case.

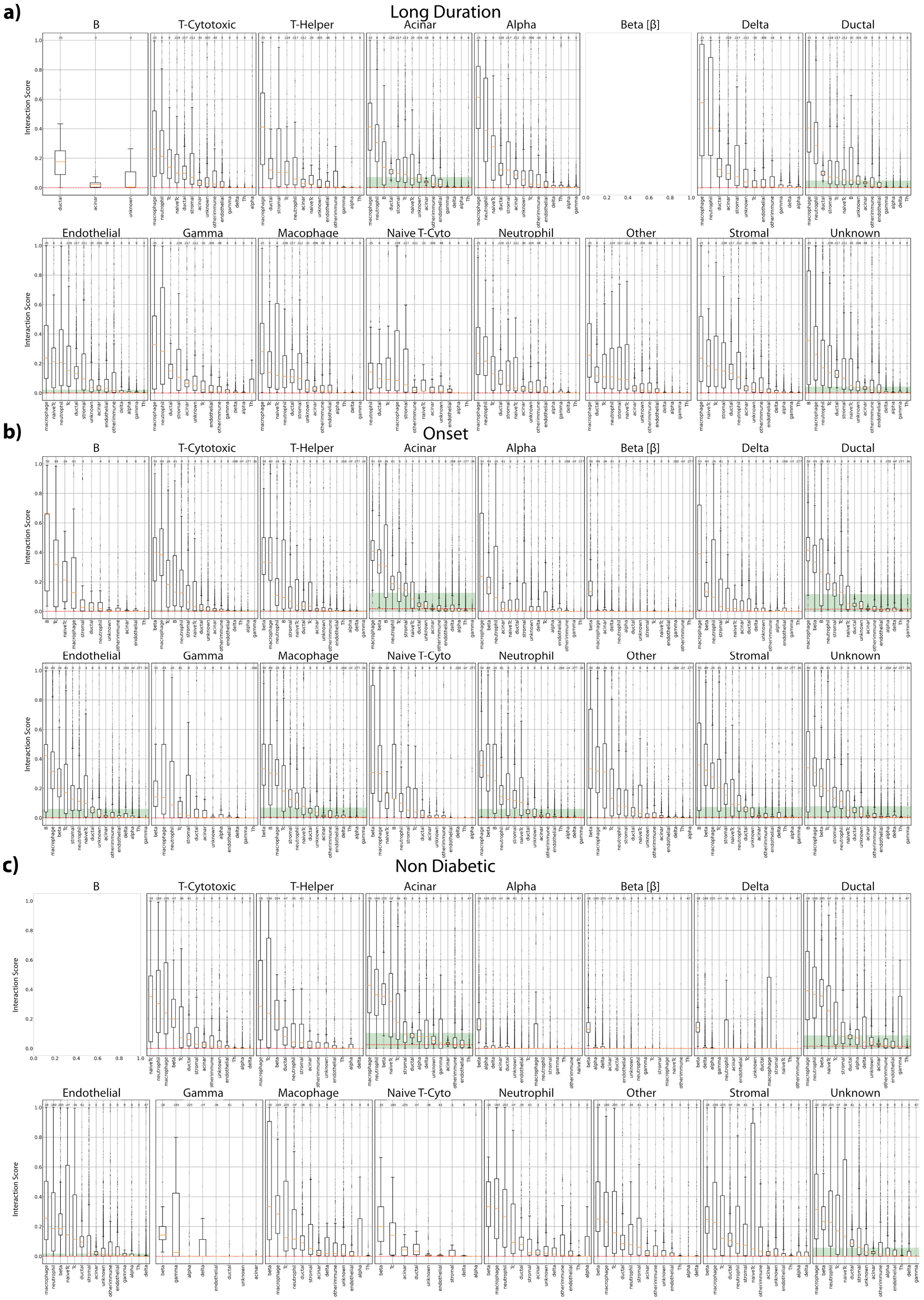


Figure S.13: **All *Interaction Scores* across regions in the HCC dataset for the bucket sampling strategy.** All immune cell type–cell type interactions identified by SHIELD in the HCC cohort are shown for each region of interest: **a)** Healthy liver, **b)** Tumor core, **c)** Rim region. Each boxplot shows the *Interaction Score* for every interaction between source and target cell types, sorted by their median value. The red dashed line indicates the median Interaction Score across all interactions; green bands represent the interquartile range (25th–75th percentile). The value above each boxplot shows the $-\log_{10}$(FDR)-corrected Mann–Whitney U test statistic. This analysis highlights phenotype-specific and spatially distinct immune interactions across tumor progression zones. If the cell type is not shown or fewer than interactions are displayed, this reflects the total number of significant interactions identified.

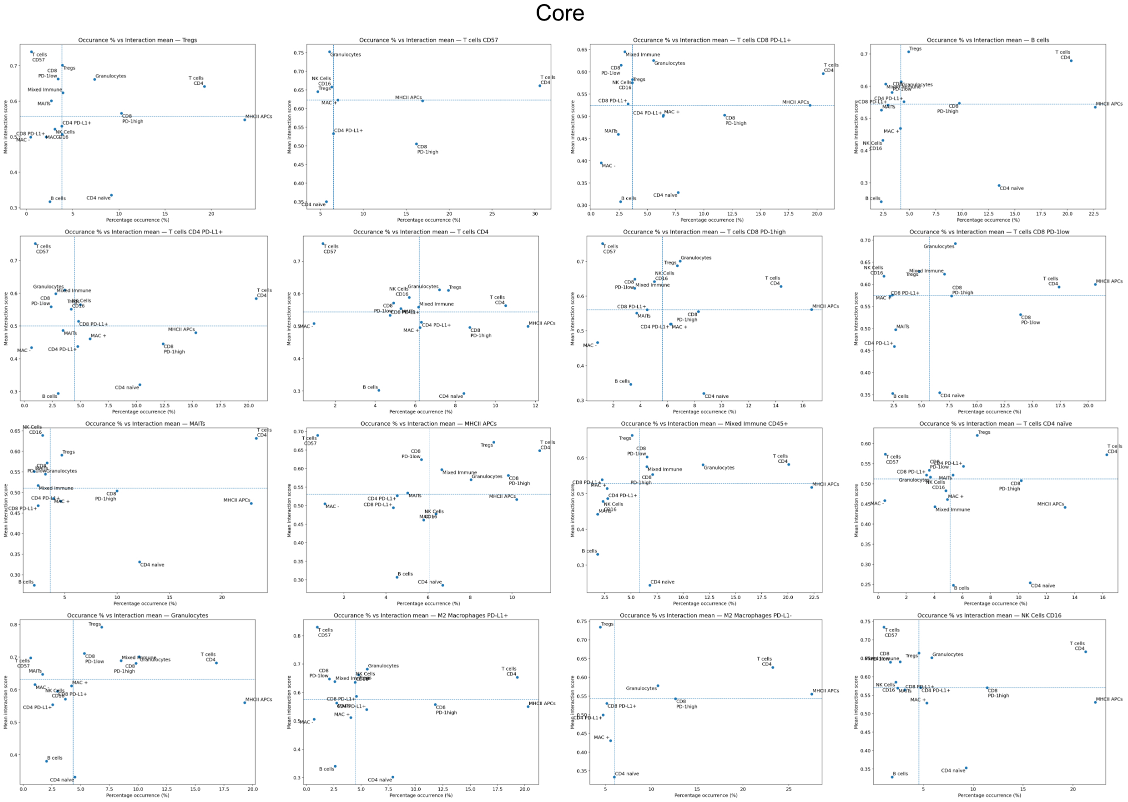


Figure S.14: **Comparison of spatial proximity and interaction relevance in the tumor core of the HCC dataset for the bucket sampling strategy.** SHIELD-derived *Interaction Scores* are plotted against the average nearest-neighbor (NN) co-occurrence percentile for each source–target cell type pair within the tumor core region. The y-axis indicates the mean Interaction Score; the x-axis shows the average percentile of target cells among the spatial neighbors of each source cell. High-scoring but low-frequency interactions in the top-left quadrant represent rare yet phenotype-relevant communication events that would be missed by proximity-based methods alone.

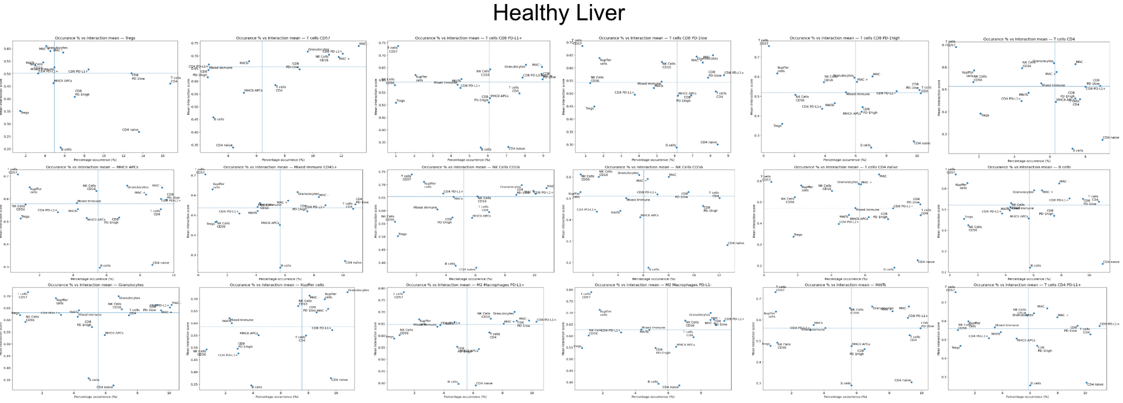


Figure S.15: **Comparison of spatial proximity and interaction relevance in the healthy liver of the HCC dataset for the bucket sampling strategy.** SHIELD-derived *Interaction Scores* are plotted against the average nearest-neighbor (NN) co-occurrence percentile for each source–target cell type pair within the healthy liver region. The y-axis indicates the mean Interaction Score; the x-axis shows the average percentile of target cells among the spatial neighbors of each source cell. High-scoring but low-frequency interactions in the top-left quadrant represent rare yet phenotype-relevant communication events that would be missed by proximity-based methods alone.

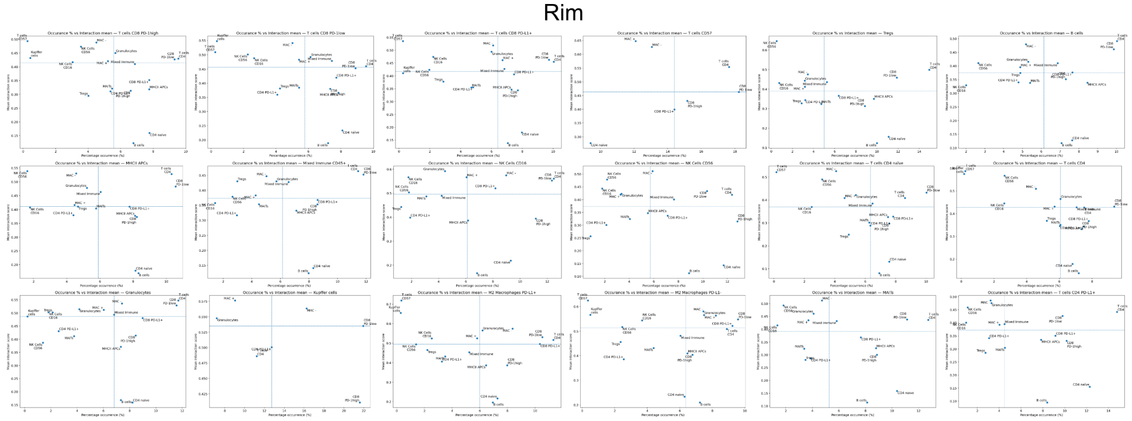


Figure S.16: **Comparison of spatial proximity and interaction relevance in the rim of the HCC dataset for the bucket sampling strategy.** SHIELD-derived *Interaction Scores* are plotted against the average nearest-neighbor (NN) co-occurrence percentile for each source–target cell type pair within the rim region. The y-axis indicates the mean Interaction Score; the x-axis shows the average percentile of target cells among the spatial neighbors of each source cell. High-scoring but low-frequency interactions in the top-left quadrant represent rare yet phenotype-relevant communication events that would be missed by proximity-based methods alone.

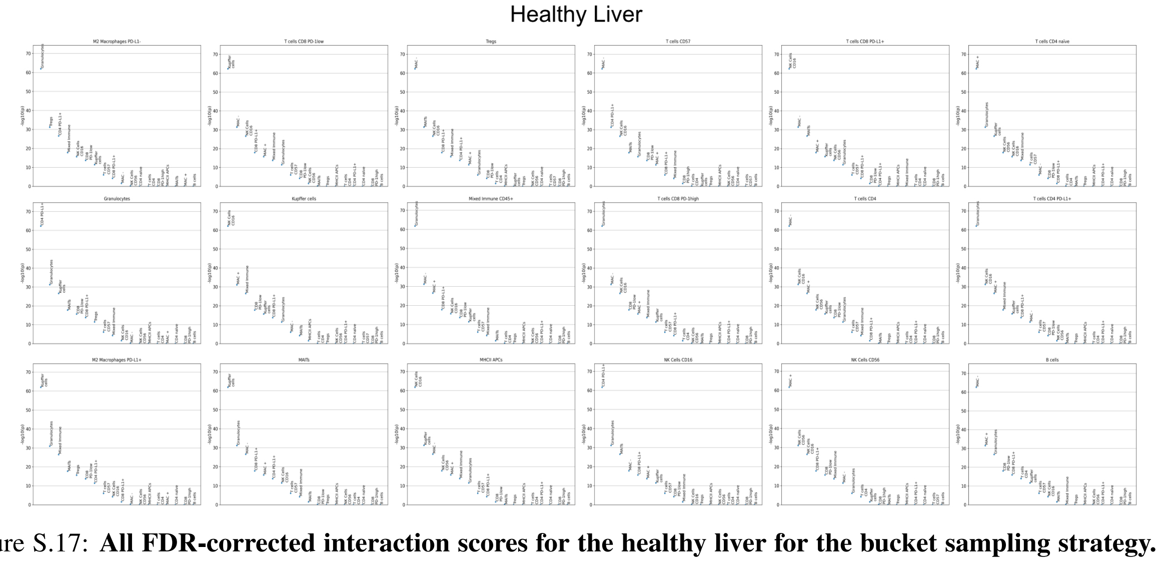


Figure S.17: **All FDR-corrected interaction scores for the healthy liver for the bucket sampling strategy.** All immune interactions within the healthy liver of the HCC cohort using $-\log_{10}$(FDR)-corrected Mann–Whitney U tests.

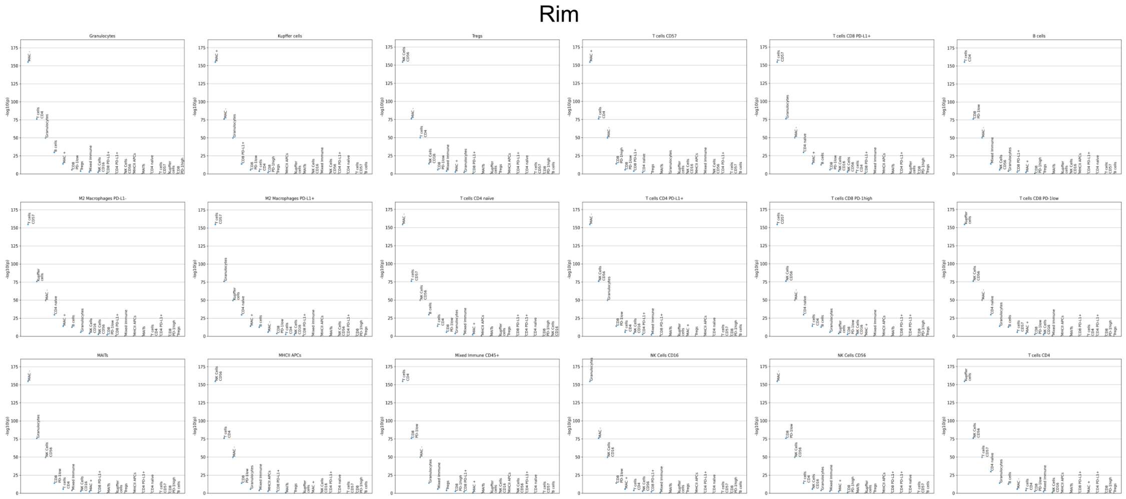


Figure S.18: **All FDR-corrected interaction scores for the rim for the bucket sampling strategy.** All immune interactions within the rim of the HCC cohort using $-\log_{10}$(FDR)-corrected Mann–Whitney U tests.

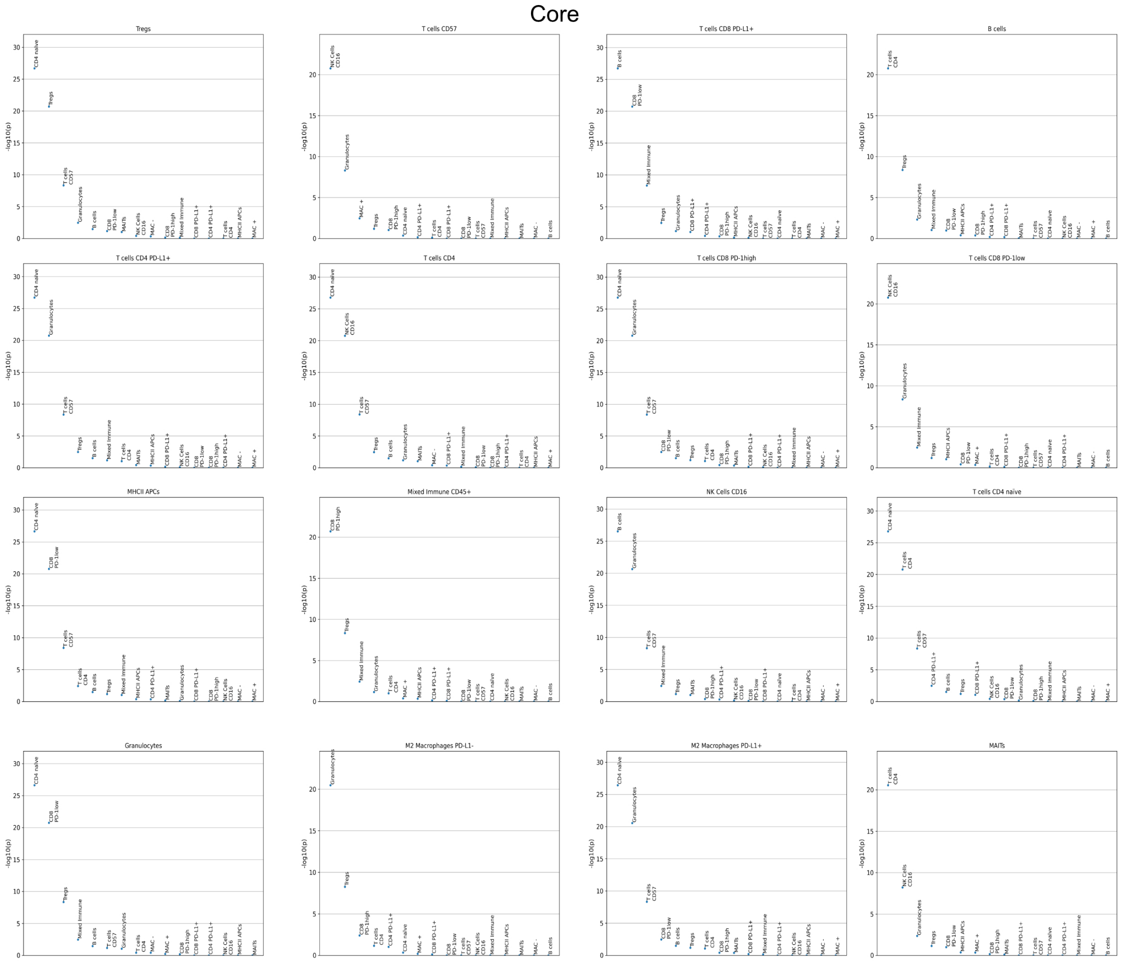


Figure S.19: **All FDR-corrected interaction scores for the tumor core for the bucket sampling strategy.** All immune interactions within the tumor core of the HCC cohort using $-\log_{10}$(FDR)-corrected Mann–Whitney U tests.

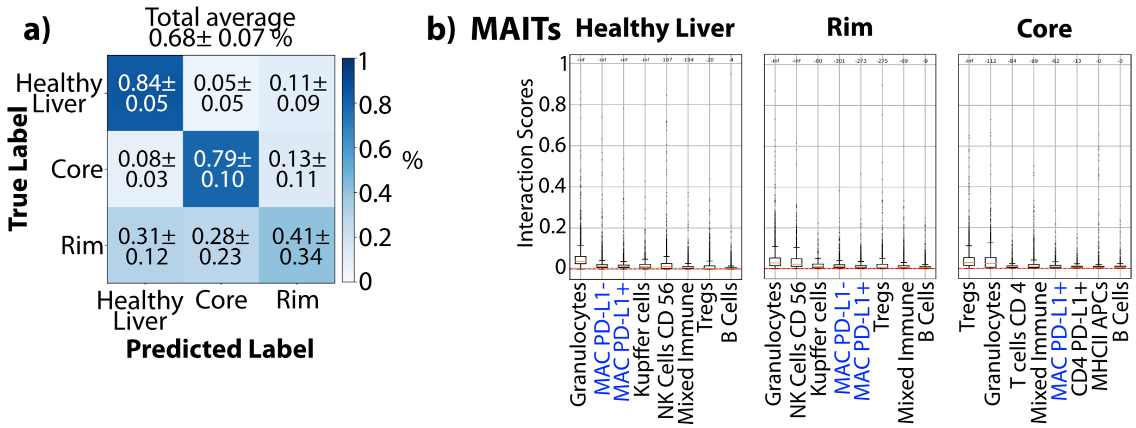


Figure S.20: **Results for the HCC dataset using the Voronoi tessellation sampling strategy.** (**a**) Confusion matrix of test predictions with the balanced total accuracy indicated in the title. (**b**) MAIT-cell Interaction Scores per ROI. The main interaction corresponding to the findings of the original study is highlighted in blue.

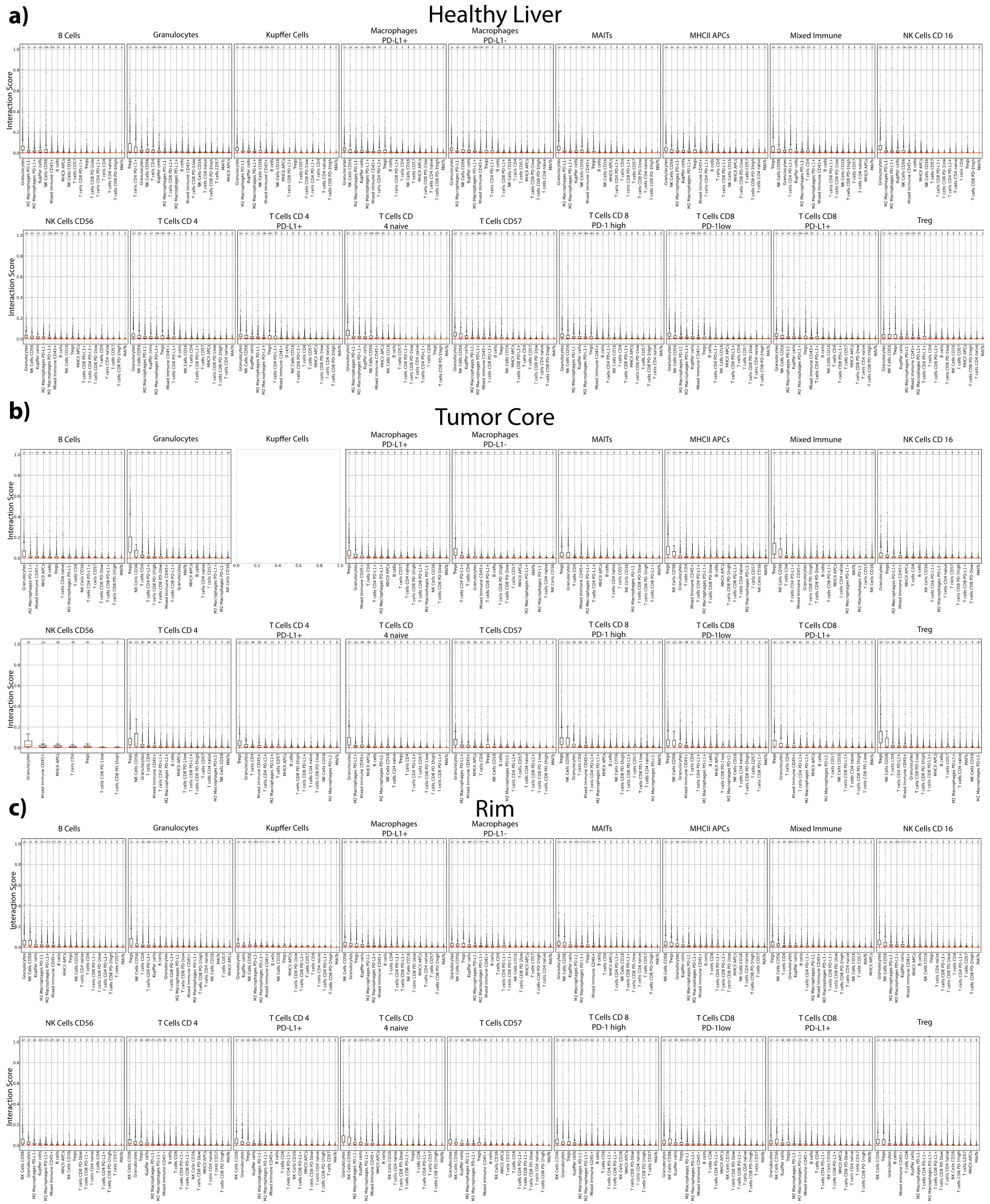


Figure S.21: **All *Interaction Scores* across regions in the HCC dataset for the Voronoi tessellation.** All immune cell type–cell type interactions identified by SHIELD in the HCC cohort are shown for each region of interest: **a)** Healthy liver, **b)** Tumor core, **c)** Rim region. Each boxplot shows the *Interaction Score* for every interaction between source and target cell types, sorted by their median value. The red dashed line indicates the median Interaction Score across all interactions; green bands represent the interquartile range (25th–75th percentile). The value above each boxplot shows the $-\log_{10}$(FDR)-corrected Mann–Whitney U test statistic. This analysis highlights phenotype-specific and spatially distinct immune interactions across tumor progression zones.

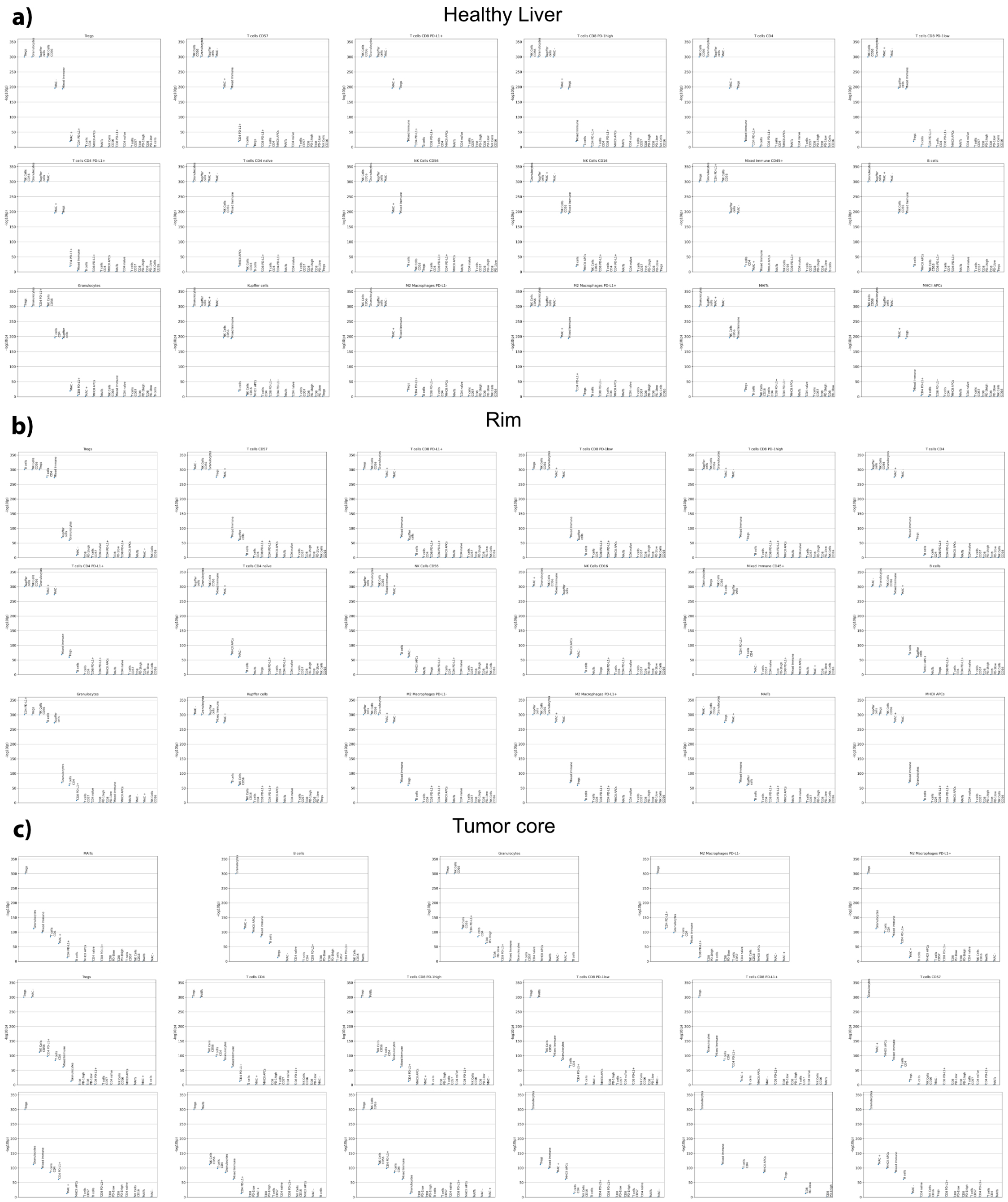


Figure S.22: **All FDR-corrected interaction scores for the Voronoi tessellation.** All immune interactions within the healthy liver of the HCC cohort using $-\log_{10}$(FDR)-corrected Mann–Whitney U tests. **a)** Healthy liver, **b)** Tumor core, **c)** Rim region.

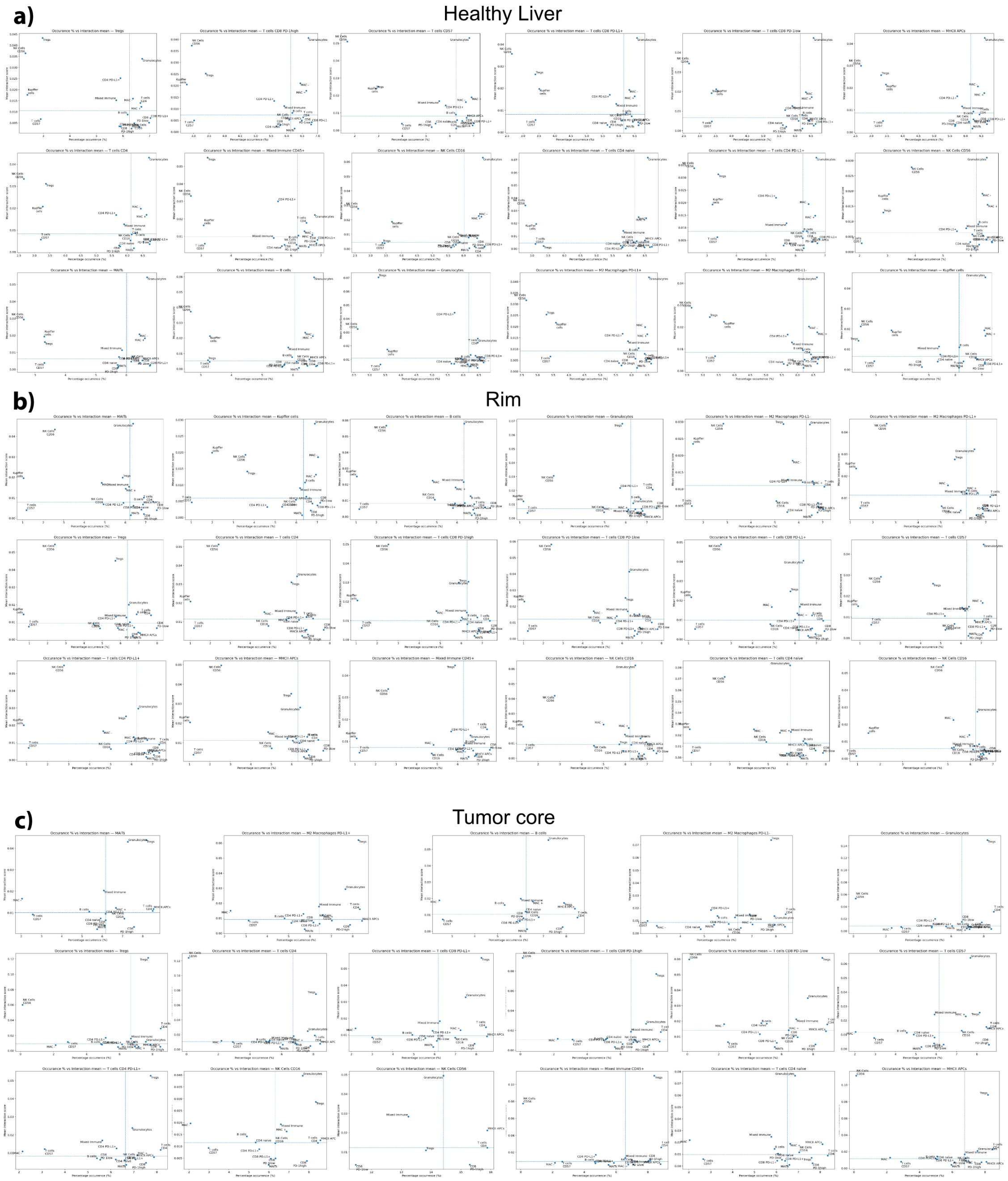


Figure S.23: **Comparison of spatial proximity and interaction relevance for all ROIs of the HCC dataset for the Voronoi tessellation.** SHIELD-derived *Interaction Scores* are plotted against the average nearest-neighbor (NN) co-occurrence percentile for each source–target cell type pair within each ROI. The y-axis indicates the mean Interaction Score; the x-axis shows the average percentile of target cells among the spatial neighbors of each source cell. High-scoring but low-frequency interactions in the top-left quadrant represent rare yet phenotype-relevant communication events that would be missed by proximity-based methods alone. **a)** Healthy liver, **b)** Tumor core, **c)** Rim region.

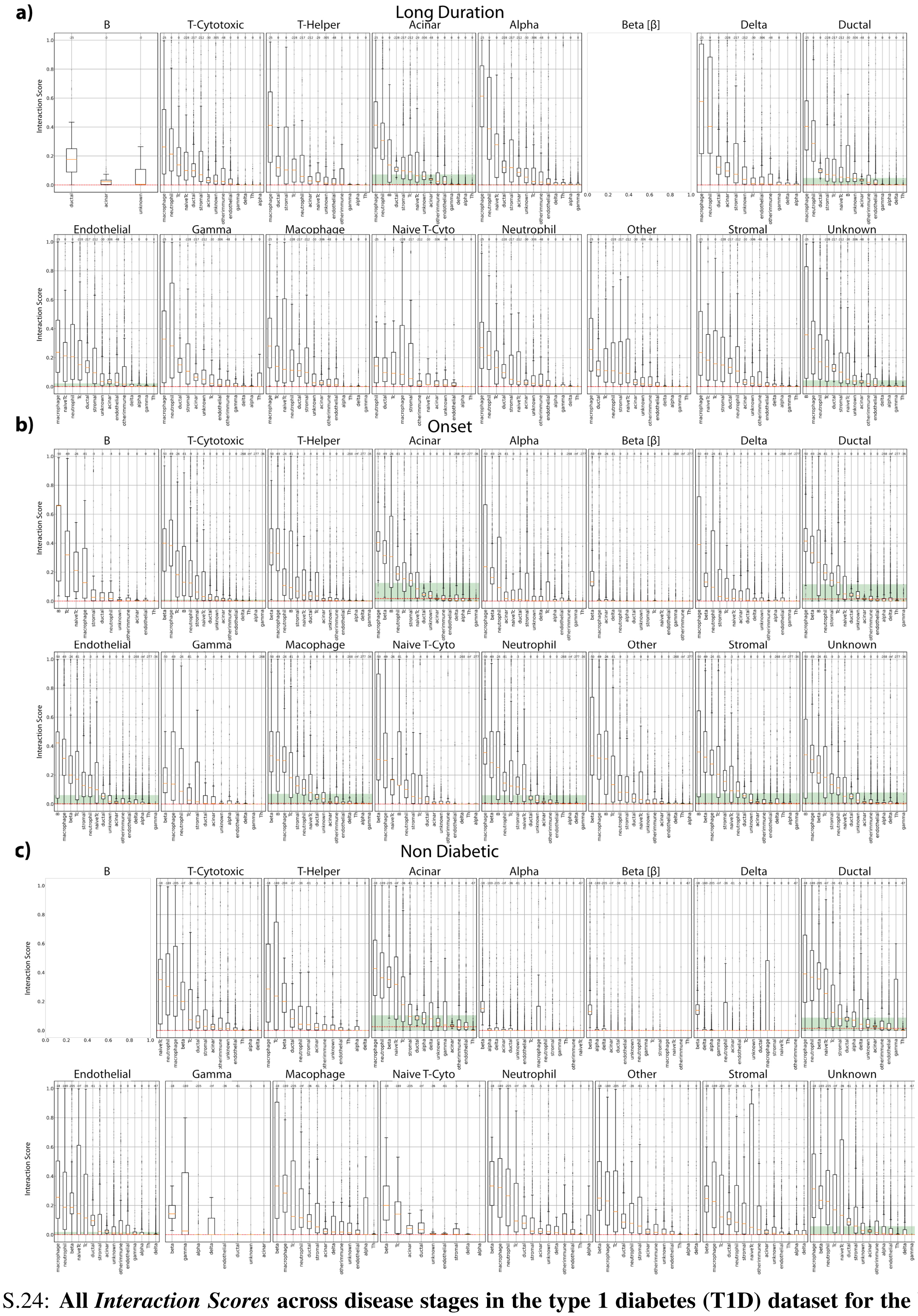


Figure S.24: **All *Interaction Scores* across disease stages in the type 1 diabetes (T1D) dataset for the Voronoi tessellation.** All immune cell type–cell type interactions identified by SHIELD in the T1D cohort are shown for each disease stage: **a)** Long-duration T1D, **b)** Onset T1D, **c)** Non-diabetic. Each boxplot shows the *Interaction Score* for every interaction between source and target cell types, sorted by their median value. The red dashed line indicates the median Interaction Score across all interactions; green bands represent the interquartile range (25th–75th percentile). The value above each boxplot indicates the $-\log_{10}$(FDR)-corrected Mann–Whitney U test statistic. This analysis reveals stage-specific shifts in immune cell–cell communication, highlighting how distinct interactions emerge or disappear during disease progression.

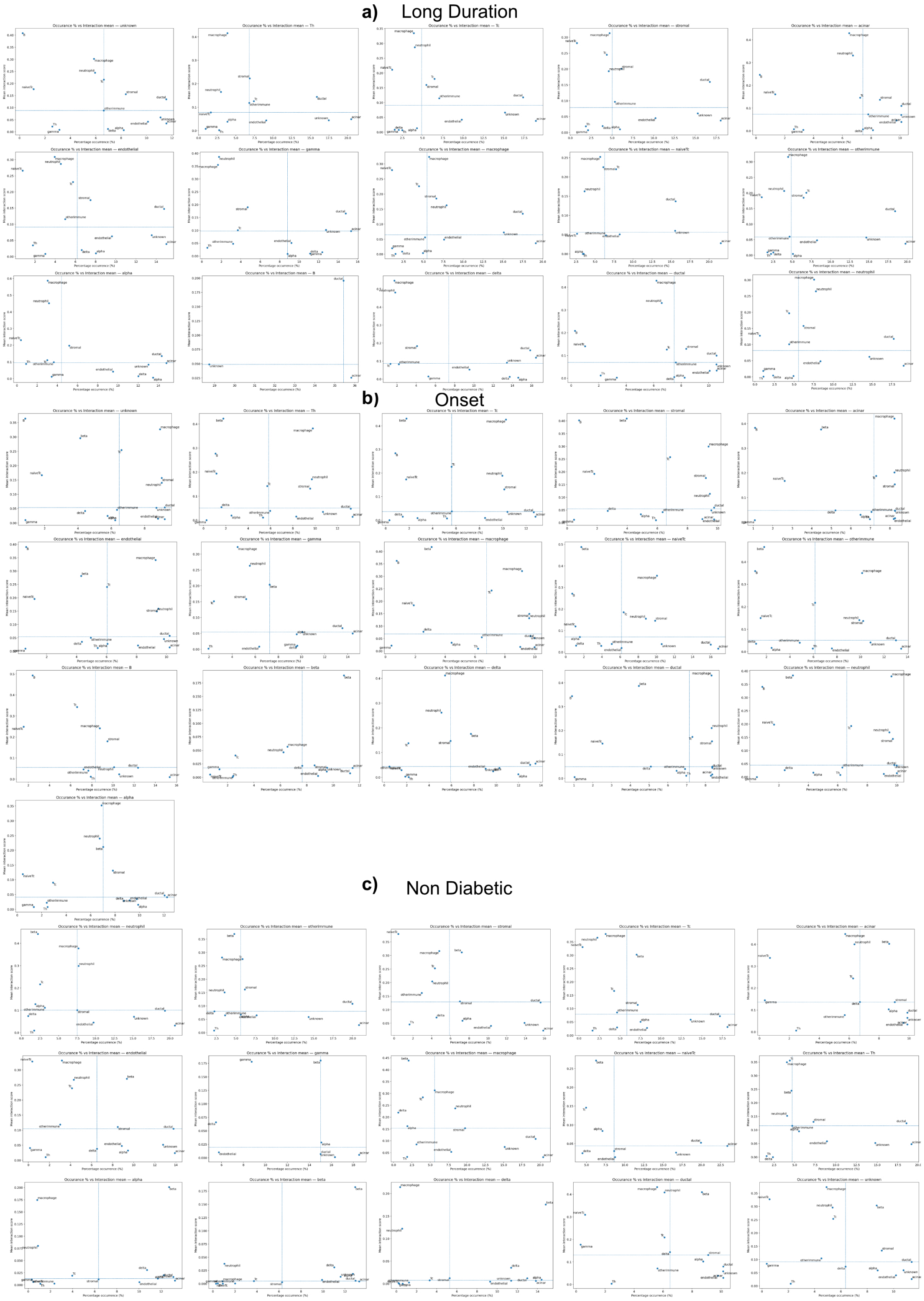


Figure S.25: **Comparison of spatial proximity and interaction relevance across disease stages in the type 1 diabetes (T1D) dataset for the Voronoi tessellation.** SHIELD-derived *Interaction Scores* are plotted against the average nearest-neighbor (NN) co-occurrence percentile for each immune cell-type pair across: **a)** Long-duration T1D, **b)** Onset T1D, **c)** Non-diabetic. The y-axis shows the mean Interaction Score for each source–target pair; the x-axis shows the average percentile of the target cell type among the nearest neighbors of the source cell. Interactions in the top-left quadrant represent rare spatial events that are nonetheless strongly associated with disease phenotype, showcasing SHIELD's ability to capture biologically meaningful, non-local cell-cell communication.

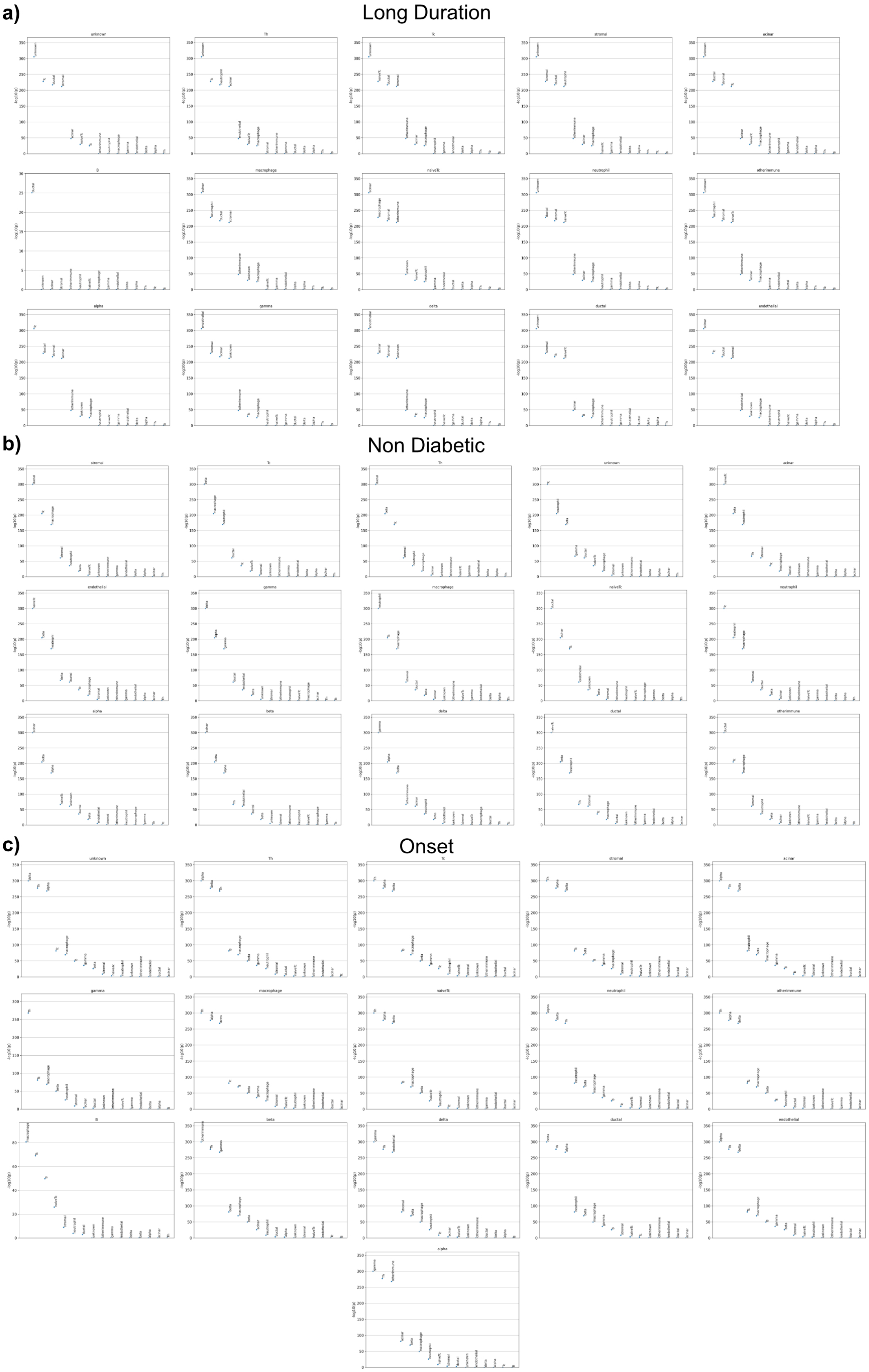


Figure S.26: **All FDR-corrected interaction scores for the diabetes dataset for the Voronoi tessellation.** All immune interactions within the diabetes cohort using $-\log_{10}$(FDR)-corrected Mann–Whitney U tests. **a)** Long Duration,**b)** Non Diabetis,**c)** Onset

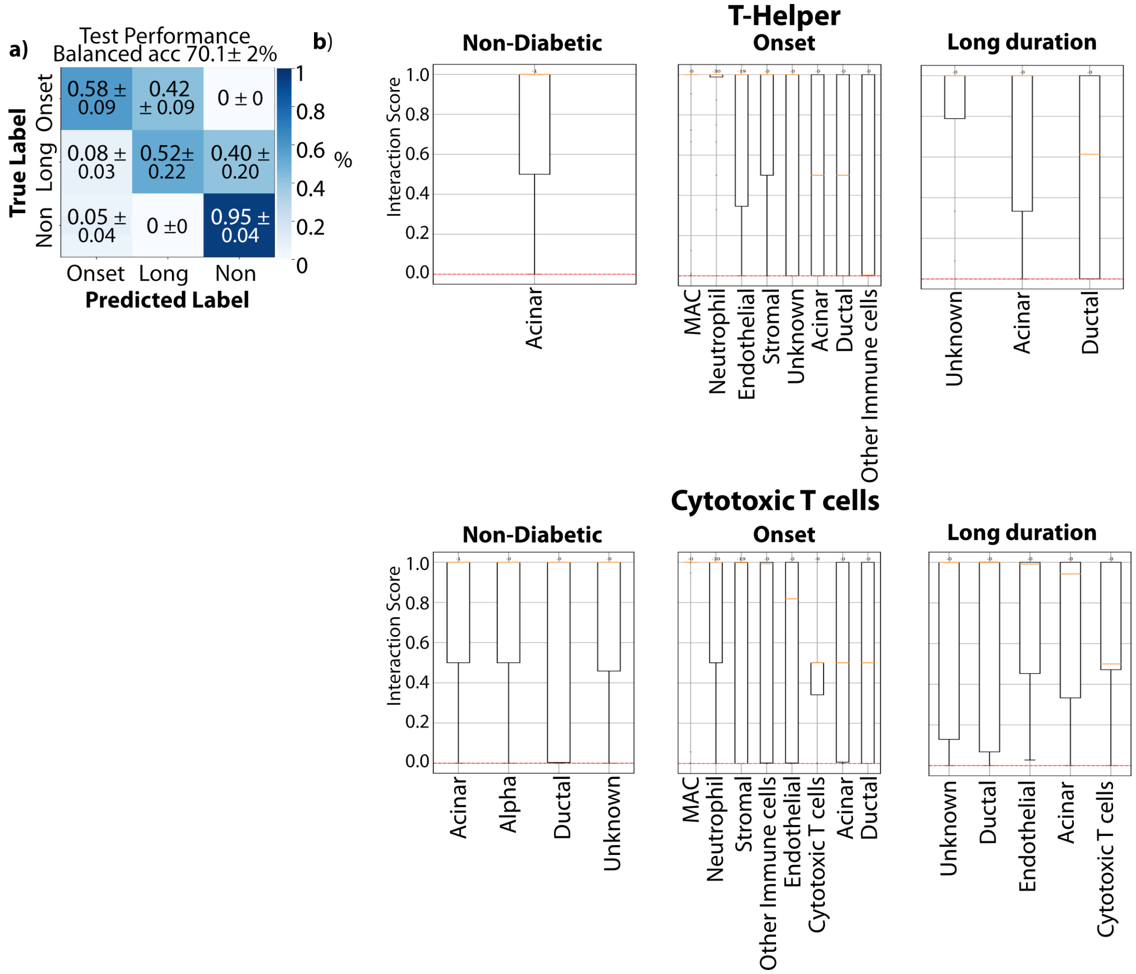


Figure S.27: **Results for the diabetes dataset using the bucket sampling strategy.** (**a**) Confusion matrix of test predictions with the balanced total accuracy indicated in the title. (**b**) The top interactions scores for the T-helper and Cytotoxic T cells

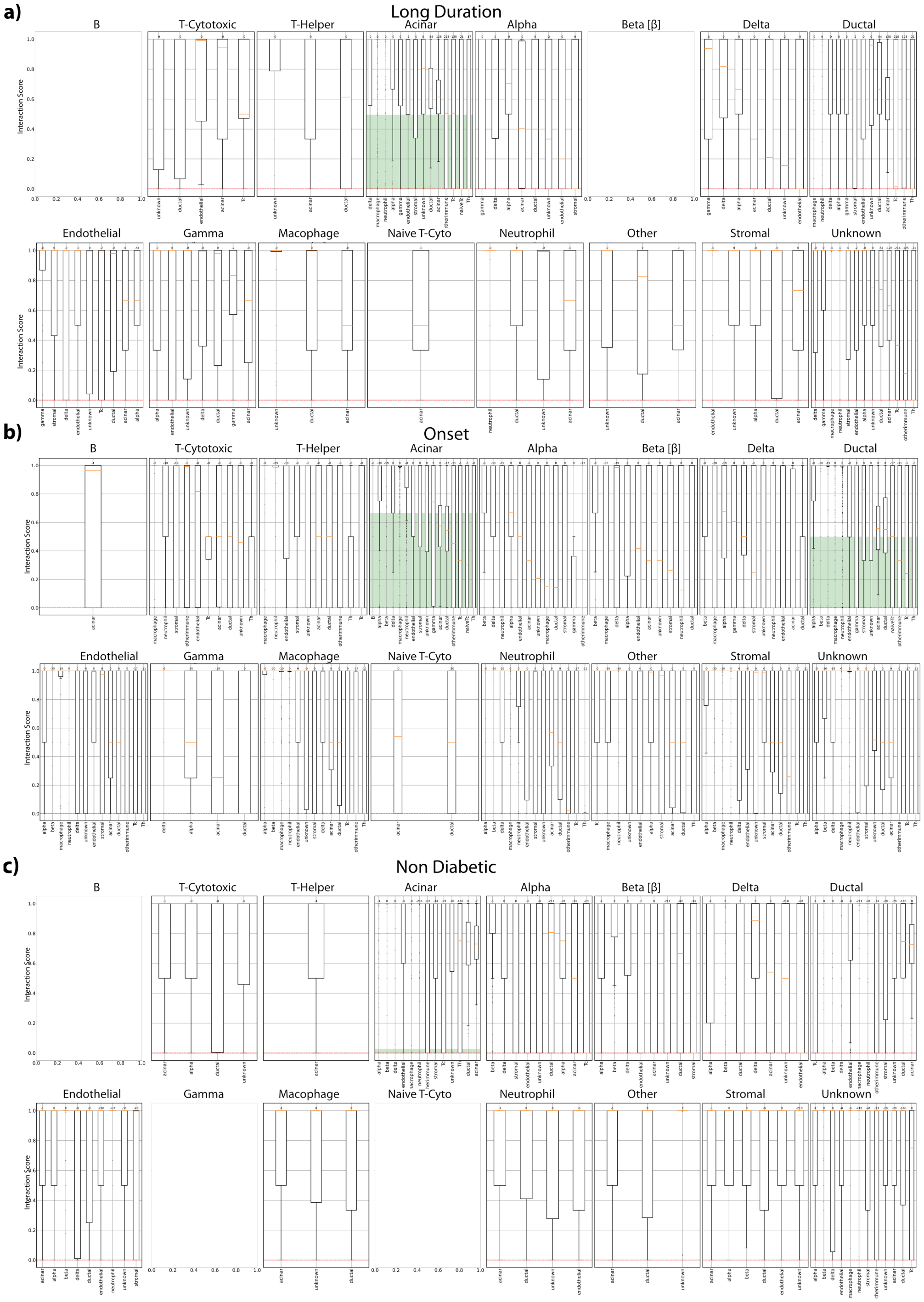


Figure S.28: **All *Interaction Scores* within the diabetes dataset for the bucket sampling.** All immune cell type–cell type interactions identified by SHIELD in the diabetes cohort are shown for each patient status: **a)** Long duration, **b)** Onset, **c)** Non diabetic. Each boxplot shows the *Interaction Score* for every interaction between source and target cell types, sorted by their median value. The red dashed line indicates the median Interaction Score across all interactions; green bands represent the interquartile range (25th–75th percentile). The value above each boxplot shows the $-\log_{10}$(FDR)-corrected Mann–Whitney U test statistic. This analysis highlights phenotype-specific and spatially distinct immune interactions across tumor progression zones.

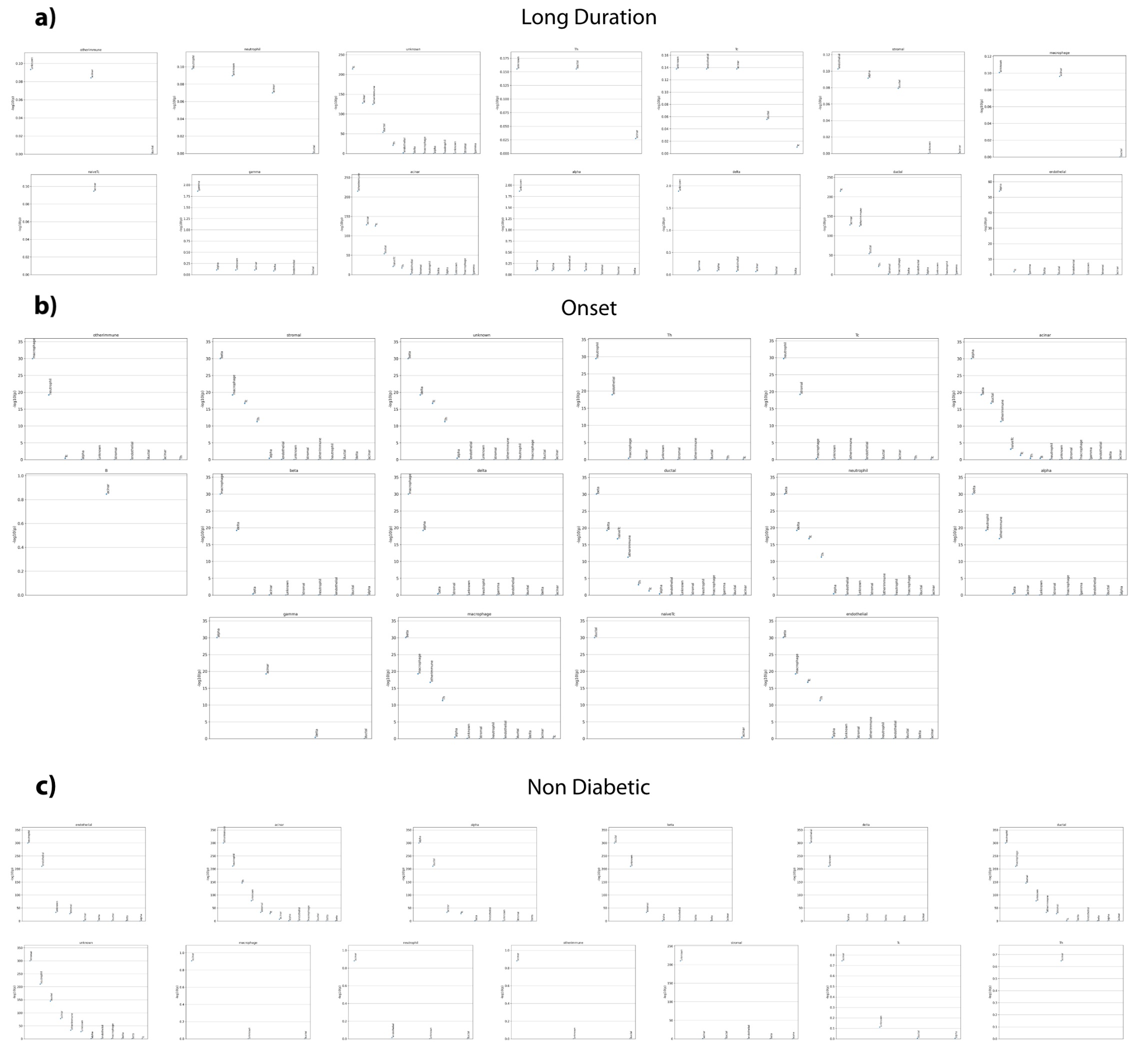


Figure S.29: **All FDR-corrected interaction scores for the bucket sampling.** All immune interactions for all patient diagnostics using the diabetes cohort $-\log_{10}$(FDR)-corrected Mann–Whitney U tests. **a)** Long duration, **b)** Onset, **c)** Non diabetic.

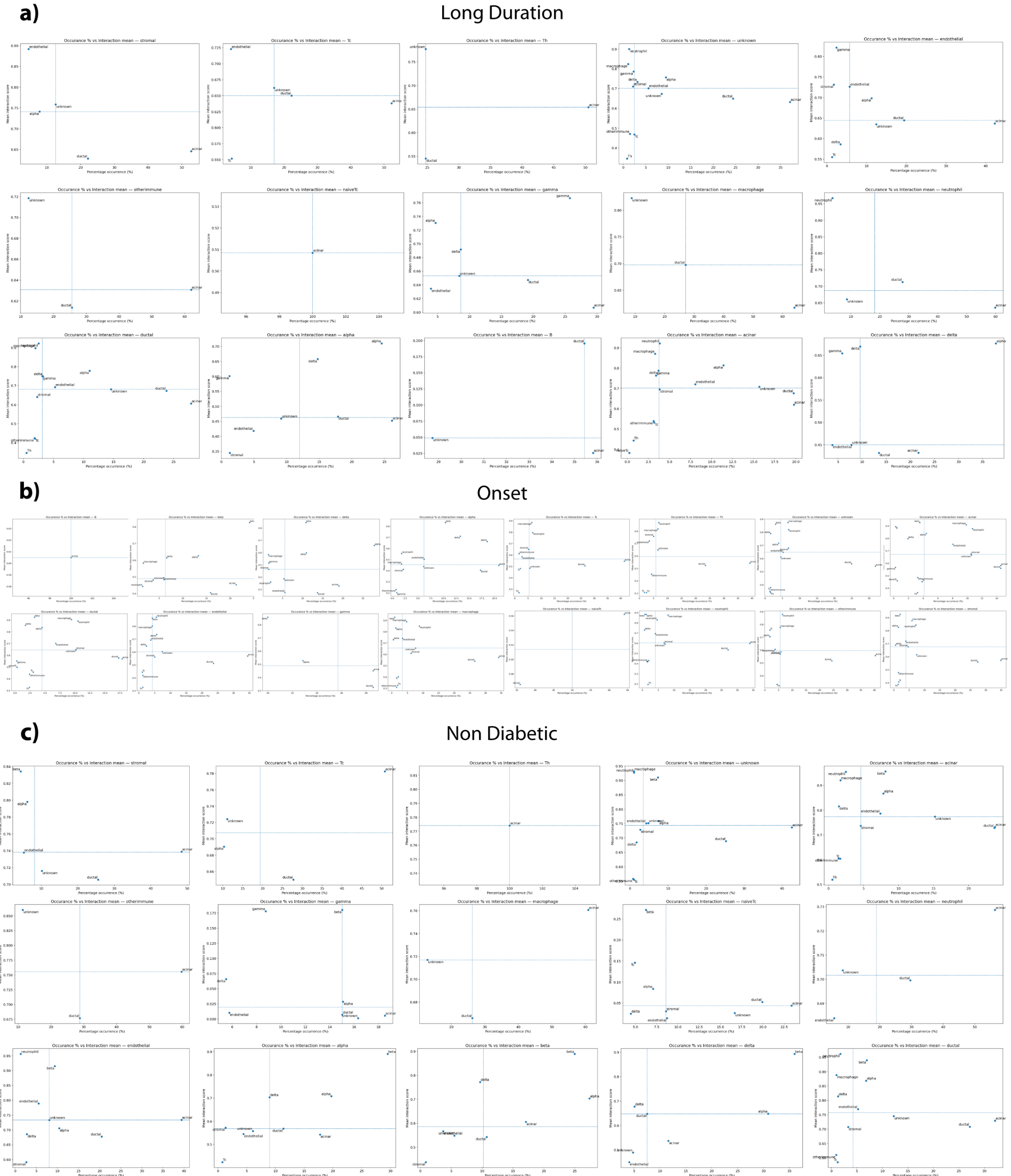


Figure S.30: **Comparison of spatial proximity and interaction relevance for each patient status within the diabetes dataset, for the bucket sampling.** SHIELD-derived *Interaction Scores* are plotted against the average nearest-neighbor (NN) co-occurrence percentile for each source–target cell type pair within each ROI. The y-axis indicates the mean Interaction Score; the x-axis shows the average percentile of target cells among the spatial neighbors of each source cell. High-scoring but low-frequency interactions in the top-left quadrant represent rare yet phenotype-relevant communication events that would be missed by proximity-based methods alone. **a)** Long duration, **b)** Onset, **c)** Non diabetic.

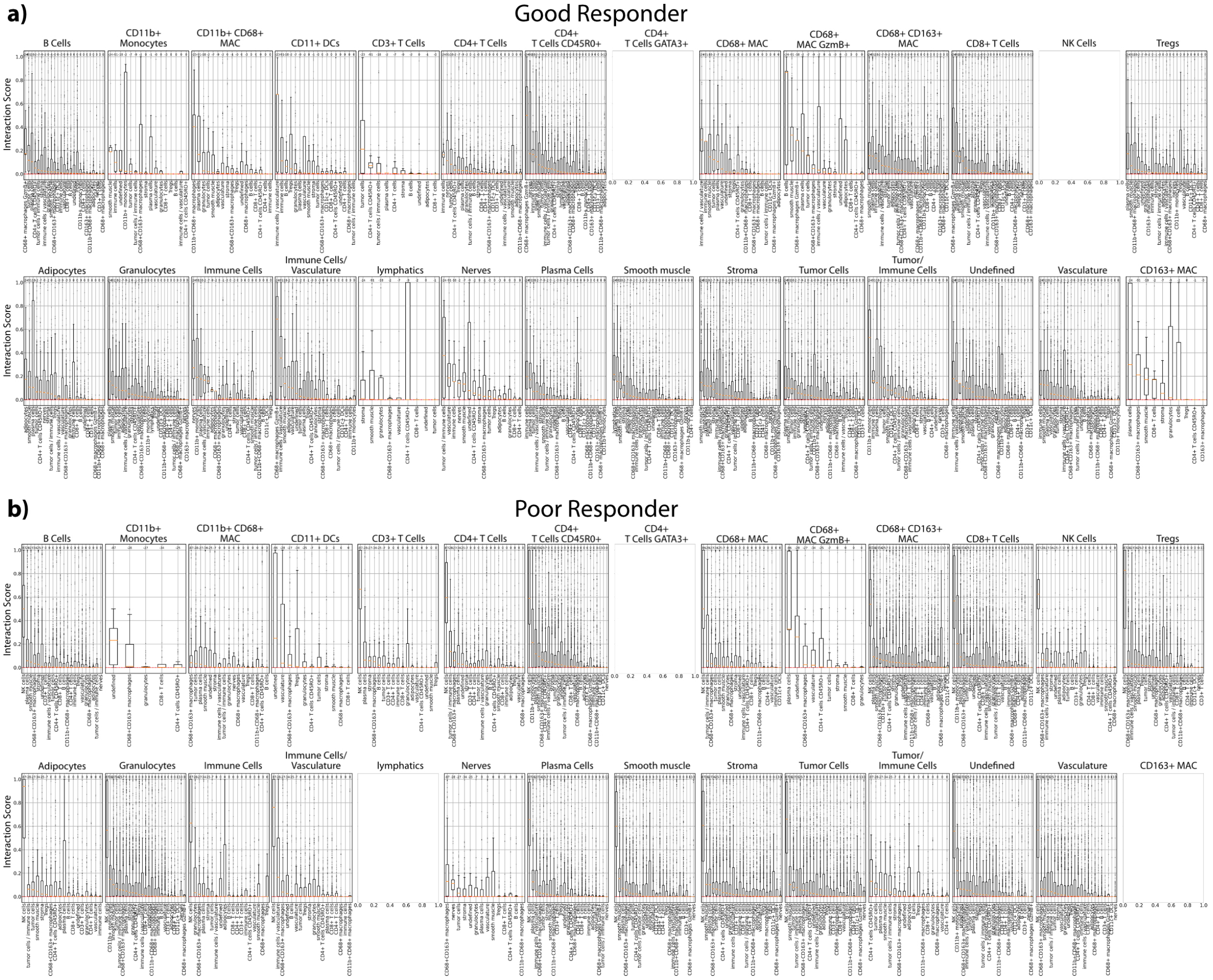


Figure S.31: **All *Interaction Scores* stratified by treatment response in the colorectal cancer (CRC) dataset for the Voronoi tessellation.** All immune cell type–cell type interactions identified by SHIELD in the CRC cohort are shown separately for: **a)** Good responders, **b)** Poor responders. Each boxplot shows the *Interaction Score* for every interaction between source and target cell types, sorted by their median value. The red dashed line marks the median Interaction Score across all interactions; green bands represent the interquartile range (25th–75th percentile). The value above each boxplot indicates the $-\log_{10}$(FDR)-corrected Mann–Whitney U test statistic. This analysis reveals response-specific immune interaction patterns, enabling identification of suppressive or activating immune circuits associated with therapy outcome.

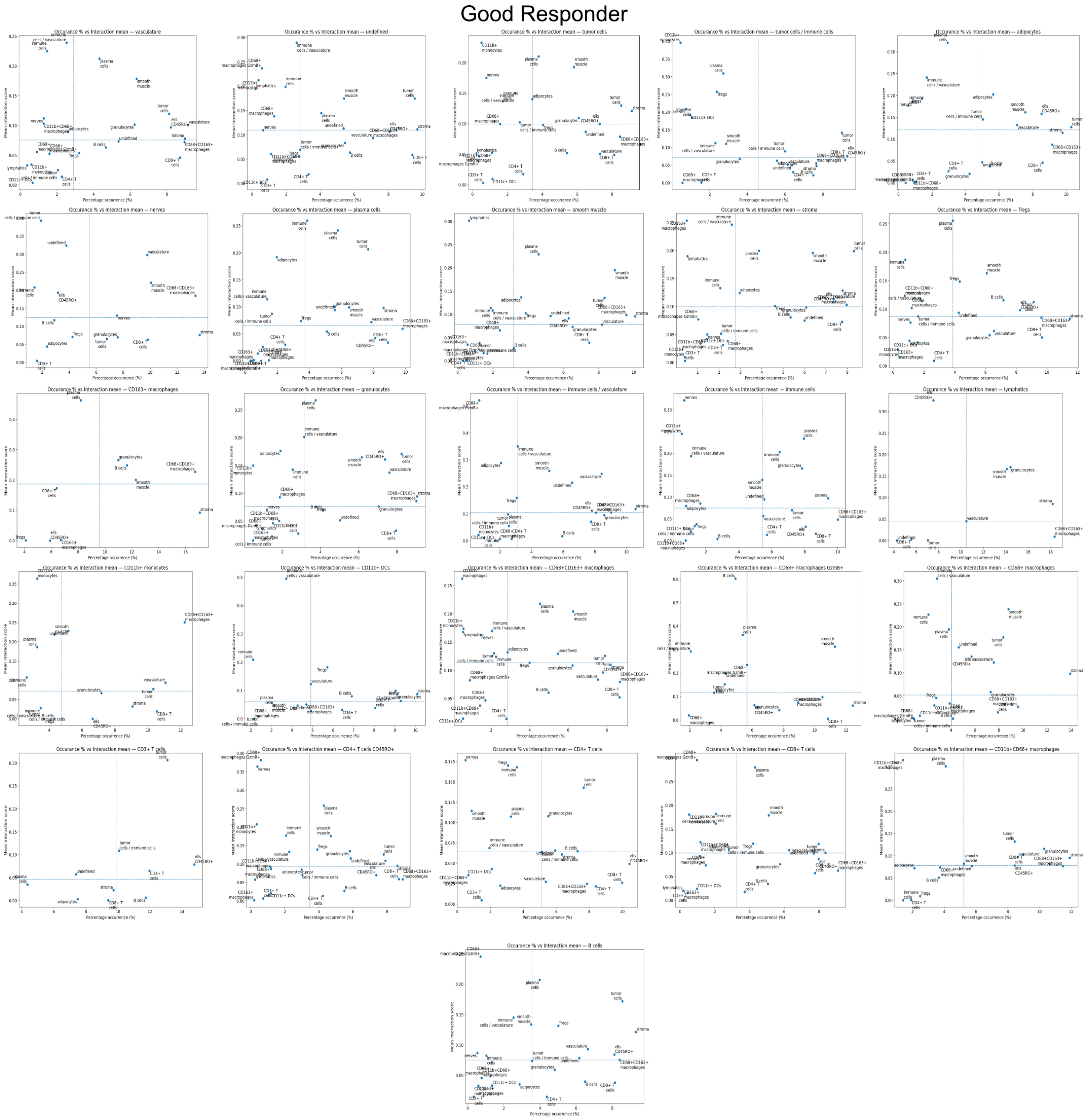


Figure S.32: **Comparison of spatial proximity and interaction relevance for good responders in the colorectal cancer (CRC) dataset for the Voronoi tessellation.** SHIELD-derived *Interaction Scores* are plotted against the average nearest-neighbor (NN) co-occurrence percentile for each immune cell-type pair within the good responder group. The y-axis indicates the mean Interaction Score; the x-axis shows the average percentile of target cells among the spatial neighbors of each source cell. Interactions in the top-left quadrant represent rare but highly phenotype-associated immune interactions, underscoring SHIELD's ability to detect functional signals that proximity-based methods would miss.

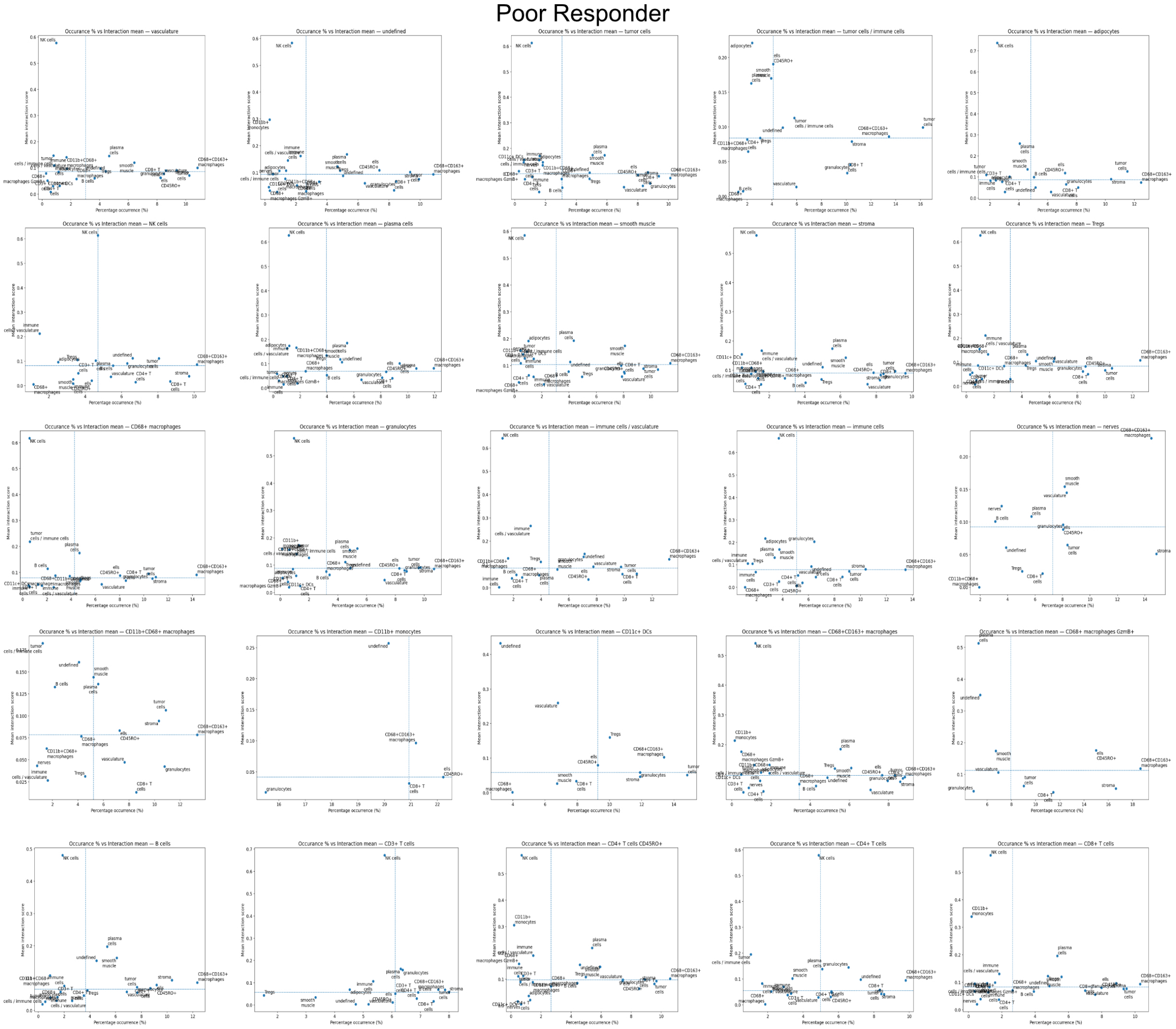


Figure S.33: **Comparison of spatial proximity and interaction relevance for poor responders in the colorectal cancer (CRC) dataset for the Voronoi tessellation.** SHIELD-derived *Interaction Scores* versus average NN co-occurrence percentile for each cell-type pair in the poor responder group. The top-left quadrant highlights rare but response-specific interactions, potentially reflecting suppressive or evasive immune mechanisms not captured by spatial co-localization alone.

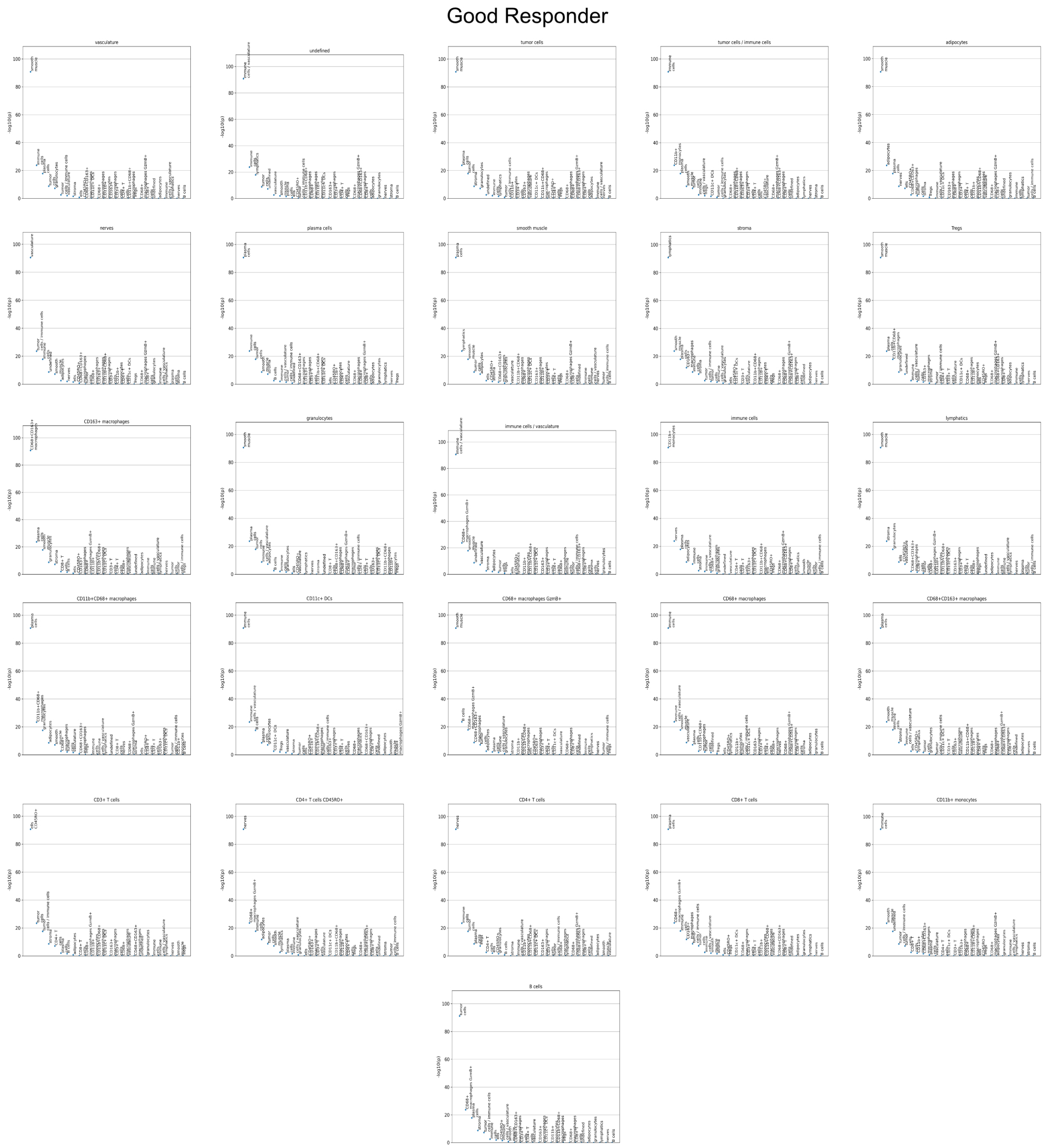


Figure S.34: **All FDR-corrected interaction scores for CRC good responders for the Voronoi tessellation.** All immune interactions within the good responders of the CRC cohort using $-\log_{10}$(FDR)-corrected Mann–Whitney U tests.

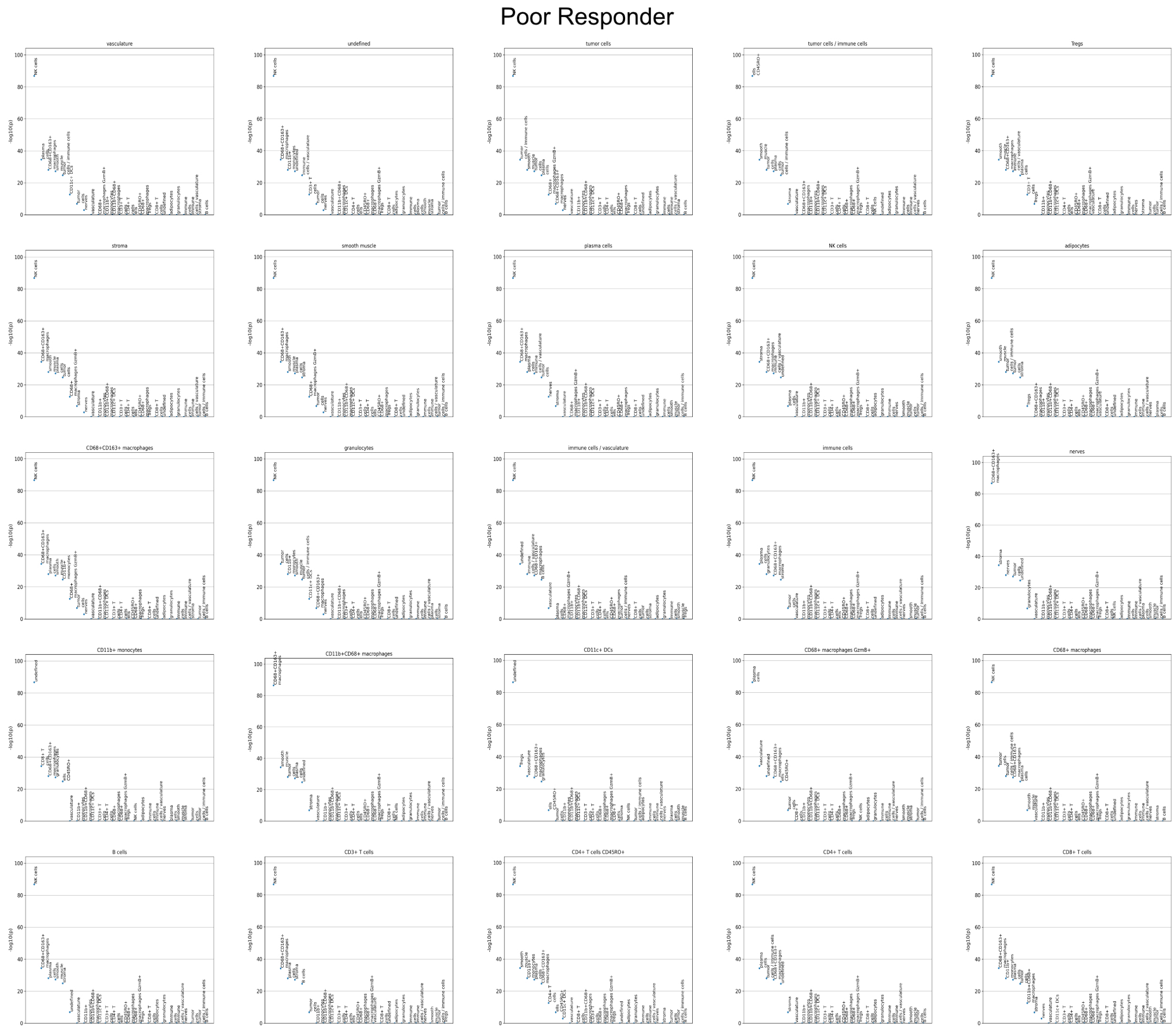


Figure S.35: **All FDR-corrected interaction scores for CRC poor responders for the Voronoi tessellation.** All immune interactions within the good responders of the CRC cohort using $-\log_{10}$(FDR)-corrected Mann–Whitney U tests.

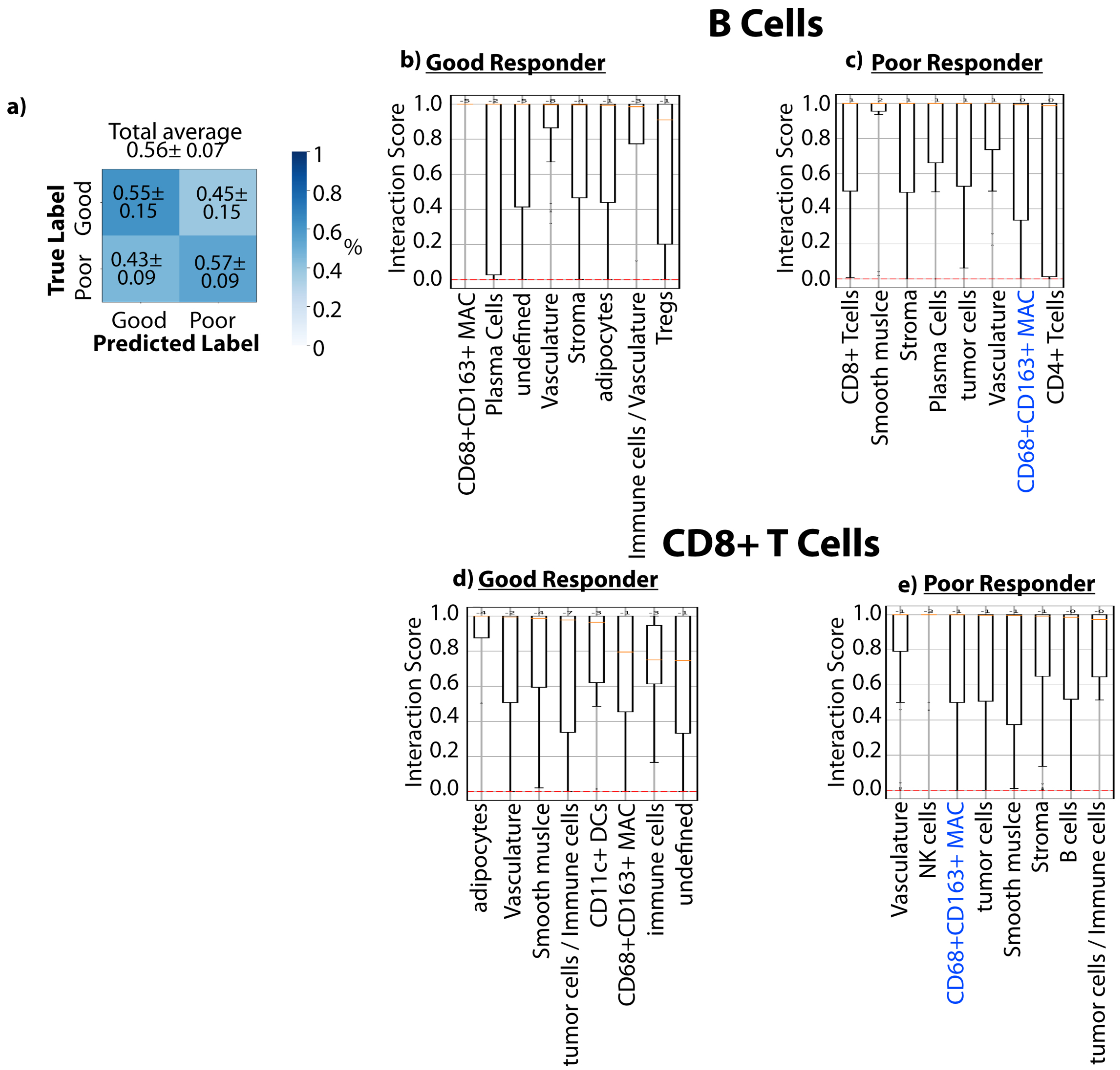


Figure S.36: **Results for the crc dataset using the bucket sampling strategy.** (**a**) Confusion matrix of test predictions with the balanced total accuracy indicated in the title. (**b**) The top interactions scores for the B cells and the CD8+ T cells

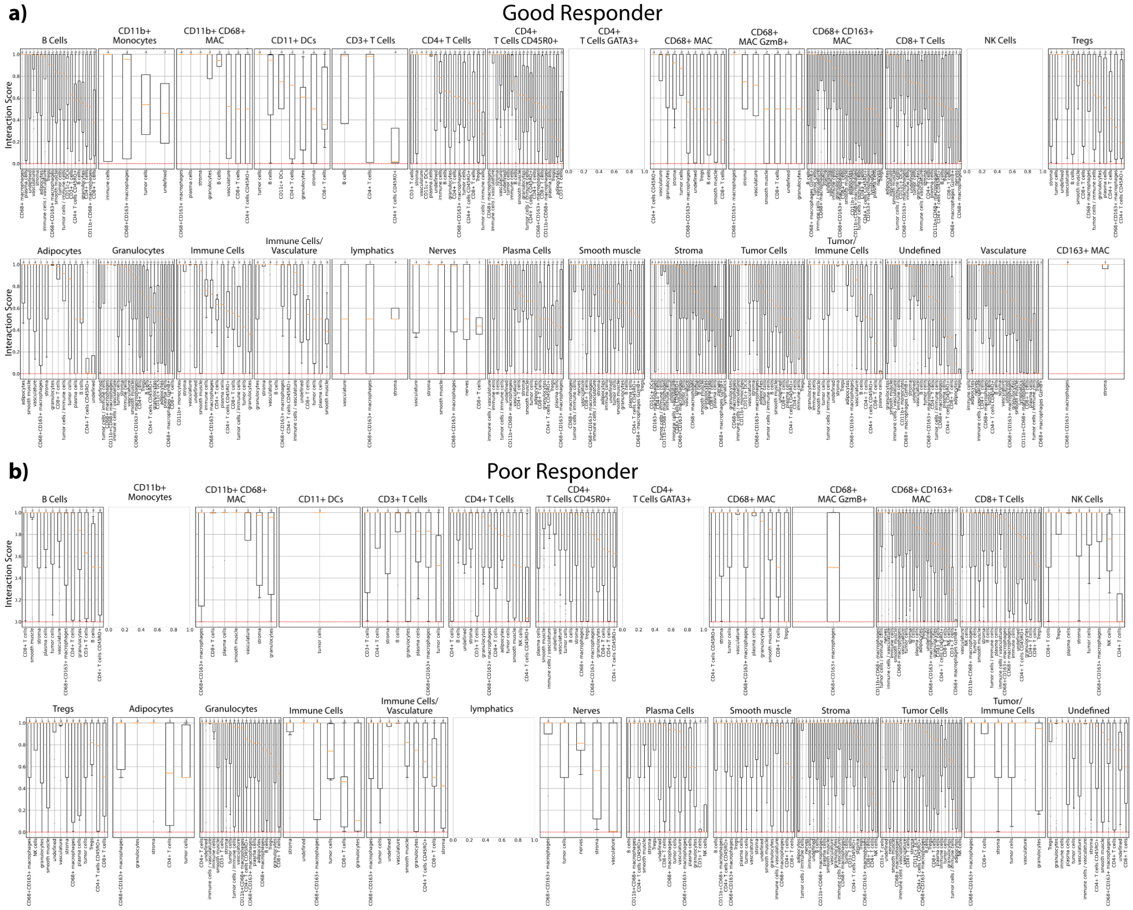


Figure S.37: **All *Interaction Scores* within the crc dataset for the bucket sampling.** All immune cell type–cell type interactions identified by SHIELD in the diabetes cohort are shown for each patient status: **a)** good responder, **b)** poor responder, Each boxplot shows the *Interaction Score* for every interaction between source and target cell types, sorted by their median value. The red dashed line indicates the median Interaction Score across all interactions; green bands represent the interquartile range (25th–75th percentile). The value above each boxplot shows the $-\log_{10}$(FDR)-corrected Mann–Whitney U test statistic.

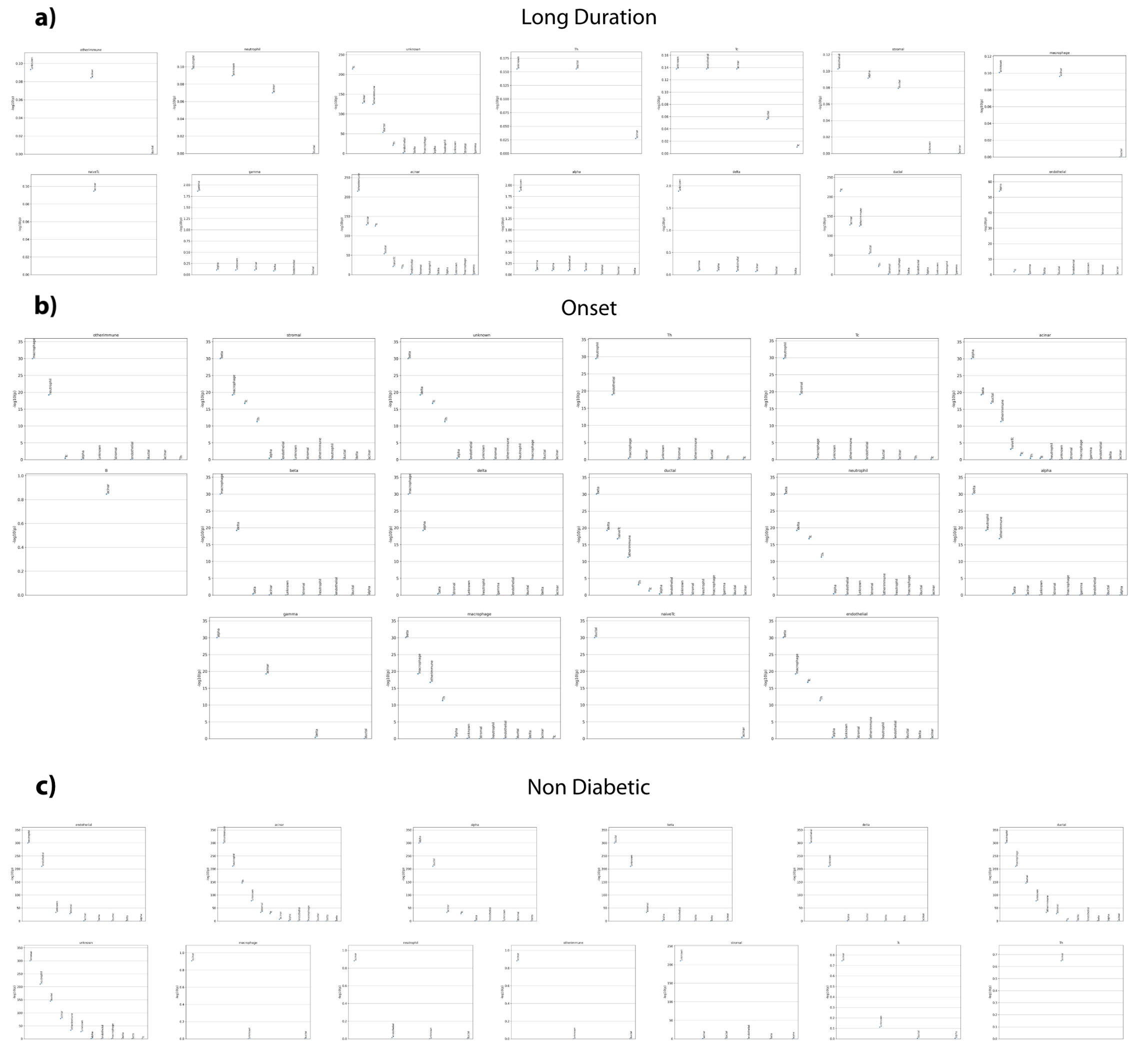


Figure S.38: **All FDR-corrected interaction scores for the bucket sampling.** All immune interactions within all patient diagnostics of the crc cohort using $-\log_{10}$(FDR)-corrected Mann–Whitney U tests. **a)** Good responder, **b)** poor responder,

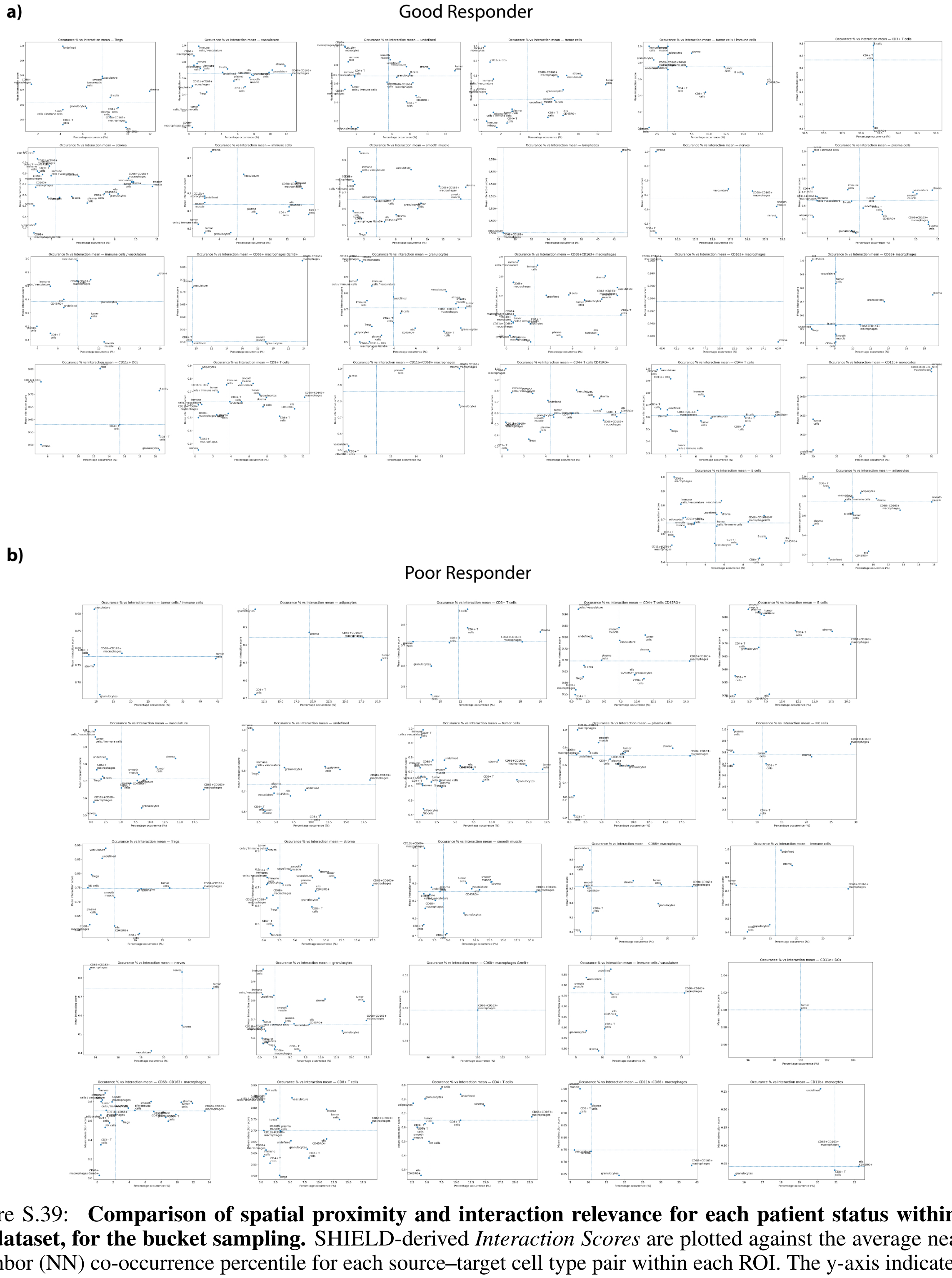


Figure S.39: **Comparison of spatial proximity and interaction relevance for each patient status within the crc dataset, for the bucket sampling.** SHIELD-derived *Interaction Scores* are plotted against the average nearest-neighbor (NN) co-occurrence percentile for each source–target cell type pair within each ROI. The y-axis indicates the mean Interaction Score; the x-axis shows the average percentile of target cells among the spatial neighbors of each source cell. High-scoring but low-frequency interactions in the top-left quadrant represent rare yet phenotype-relevant communication events that would be missed by proximity-based methods alone. **a)** good responder, **b)** poor responder,
